# Evolution and diversification of the O-methyltransferase (OMT) gene family in Solanaceae

**DOI:** 10.1590/1678-4685-GMB-2023-0121

**Published:** 2023-11-10

**Authors:** Pedro Henrique Pezzi, Leonardo Tresoldi Gonçalves, Maríndia Deprá, Loreta Brandão de Freitas

**Affiliations:** 1 Universidade Federal do Rio Grande do Sul, Departamento de Genética, Porto Alegre, RS, Brazil.

**Keywords:** Anthocyanin, flavonoid, functional diversification, secondary metabolites, Solanum

## Abstract

O-methyltransferases (OMTs) are a group of enzymes involved in several fundamental biological processes in plants, including lignin biosynthesis, pigmentation, and aroma production. Despite the intensive investigation of the role of OMTs in plant secondary metabolism, the evolution and diversification of this gene family in Solanaceae remain poorly understood. Here, we conducted a genome-wide survey of OMT genes in six Solanaceae species, reconstructing gene phylogenetic trees, predicting the potential involvement in biological processes, and investigating the exon/intron structure and chromosomal location. We identified 57 caffeoyl-CoA OMTs (CCoAOMTs) and 196 caffeic acid OMTs (COMTs) in the studied species. We observed a conserved gene block on chromosome 2 that consisted of tandem duplicated copies of OMT genes. Our results suggest that the expansion of the OMT gene family in Solanaceae was driven by whole genome duplication, segmental duplication, and tandem duplication, with multiple genes being retained by neofunctionalization and subfunctionalization. This study represents an essential first step in unraveling the evolutionary history of OMTs in Solanaceae. Our findings deepen our understanding of the crucial role of OMTs in several biological processes and highlight their significance as potential biotechnological targets.

## Introduction

S-adenosyl-l-methionine (SAM)-dependent O-methyltransferases (OMTs) are a diverse group of multifunctional enzymes that catalyze the transfer of a methyl group from SAM to multiple acceptor molecules, producing the corresponding methyl ether derivatives (Lam et al., 2007). This gene family can be divided into two subfamilies according to the substrate they methylate, their protein characteristics, and conserved motifs: caffeoyl-CoA O-methyltransferases (CCoAOMTs) are mainly responsible for caffeoyl-CoA methylation, whereas caffeic acid O-methyltransferases (COMTs) uses caffeic acid and a myriad of other molecules as a substrate (Davin and Lewis, 1992; Joshi and Chiang, 1998; Li et al., 2006). 

OMT proteins play significant roles in several fundamental biological processes in plants. These proteins are involved in the biosynthesis of diverse essential metabolites for plant growth, development, and defense-including alkaloids, flavonoids, lignin, and phenylpropanoids (Lam et al., 2007). Notably, OMT proteins are crucial for lignin biosynthesis, where CCoAOMT and COMT proteins contribute to the process (Bonawitz and Chapple, 2010). However, the scope of OMT protein functionality extends far beyond lignin biosynthesis, as OMT proteins are also involved in flower pigmentation (Akita et al., 2011) and aroma production (Wu et al., 2003), stress response (Hafeez et al., 2021), and melatonin biosynthesis (Byeon et al., 2014). These findings indicate that OMT proteins could be involved in various biological processes, many of which remain unknown. The broad range of protein functions makes them potentially crucial targets for genetic manipulation to enhance plant productivity, defense, and adaptation to environmental stresses (Struck et al., 2012).

Solanaceae, known as the nightshade family, is one of the largest and most diverse plant families, comprising over 90 genera and 3000 species (Särkinen et al., 2013; Gebhardt, 2016). Many species in this family have great economic significance, including tomato, potato, eggplant, tobacco, and chili peppers. With the recent advances in genomics and the availability of entire genomes, the evolutionary history of the OMT gene family has been explored in various crops, such as *Citrus sinensis* (Liu et al., 2016), *Vitis vinifera* (Lu et al., 2022), and *Gossypium* spp. (Hafeez et al., 2021). The function and substrate of OMT enzymes have been extensively studied in *Arabidopsis thaliana* and *Oryza sativa*, resulting in 12 and 11 functionally annotated and curated entries in UniProt (2023), primarily associated with lignin production. However, the evolution, diversification, and potential functions of OMT genes are still unknown in Solanaceae.

In this study, we conducted a comprehensive survey of the OMT gene family in Solanaceae genomes to gain insights into their evolutionary history. Specifically, we focused on six species representing the most significant genera in Solanaceae, to achieve four main objectives: 1) reconstructing the phylogeny of CCoAOMT and COMT subfamilies; 2) predicting their potential involvement in biological and molecular processes; 3) identifying their potential substrates; and 4) investigating their exon/intron structure and chromosomal location. This study is the first step toward unraveling the evolutionary history of the OMT gene family in Solanaceae. Our findings pave the way for further research into the crucial role of this gene family in various essential biological processes.

## Material and Methods

### Identification and filtering of OMT genes

We downloaded the predicted protein sequences of 23 Solanaceae annotated genomes (Table S1) available on the Sol Genomics Network (2023) (Fernandez-Pozo et al., 2015) and the online database National Center for Biotechnology Information (NCBI, 2023). To retrieve the candidate OMT genes, we used the Pfam (2023) domains PF00891 (COMT subfamily) and PF01596 (CCoAOMT subfamily) as queries in HMMER v.3.2.1 using the *hmmsearch* tool with a cutoff value of 0.01. In the final dataset we kept only the sequences longer than 200 and 180 amino acid residues (aa) for the COMT and CMCoAOMT subfamilies, respectively. 

After observing the initial results, which revealed that congeneric sequences overall clustered together on the phylogenetic tree (see Results section for further details), we conducted downstream analyses using a single representative of each genus. These representatives included *Capsicum annuum, Datura stramonium*, *Iochroma cyaneum*, *Nicotiana attenuata*, *Petunia axillaris*, and *Solanum lycopersicum* (Table 1). When we selected the representatives of genera with multiple species, we prioritized diploid species and those with a genomic assembly at the chromosomal level to capture the segmental evolutionary process instead of whole duplication processes.

**Table 1-:** Gene count and reference for each species in the filtered dataset.

Species	Code	Chr number (n)	CCoAOMT	COMT	Total	Reference	Source
*Capsicum annuum*	Can	12	8	39	47	Kim et al. (2014)	Sol Genomics Network (2023)
*Datura stramonium*	Dst	12	7	33	40	Rajewski et al. (2021)	GenBank SAMN14375310
*Iochroma cyaneum*	Icy	12	14	41	55	Powell et al. (2022)	Sol Genomics Network (2023)
*Nicotiana attenuatta*	Nat	12	8	27	36	Xu et al. (2017)	Sol Genomics Network (2023)
*Petunia axillaris*	Pax	7	8	33	41	Bombarely et al. (2016)	Sol Genomics Network (2023)
*Solanum lycopersicum*	Sly	12	11	23	34	The Tomato Genome Consortium (2012)	Sol Genomics Network (2023)
*Arabidopsis thaliana*	Ath	5	7	17	24	The Arabidopsis Information Resource (2023)	https://www.arabidopsis.org/
*Citrus sinensis*	Csi	19	6	52	58	Xu et al. (2013)	Citrus Pan-genome to Breeding Database
*Populus trichocarpa*	Ptr	9	6	29	35	Tuskan et al. (2006)	ENSEMBL Plants
*Vitis vinifera*	Vvi	19	10	37	47	The French-Italian Public Consortium for Grapevine Genome Characterization (2007)	ENSEMBL Plants

### Phylogeny reconstruction

We selected four outgroup species for which the OMT gene family has been well-characterized to include in the phylogeny: *Arabidopsis thaliana* (Hafeez et al., 2021), *Citrus sinensis* (Liu et al., 2016), *Populus trichocarpa* (Barakat et al., 2011; Lu et al., 2022), and *Vitis vinifera* (Lu *et al.*, 2022). References for each dataset are listed in Table S1. When genes showed splicing variation, we kept only the longest sequence. The dataset was aligned using MAFFT v.7 (Katoh et al., 2019) and trimmed with trimAL (Capella-Gutiérrez et al., 2009) using the *-gt* parameter of 0.25. We used ModelFinder (Kalyaanamoorthy et al., 2017) and the Bayesian information criterion (BIC) score to select the best-fit substitution model as implemented in the IQ-TREE webserver (Trifinopoulos et al., 2016). We used the IQ-TREE webserver to construct a maximum likelihood phylogenetic tree with 1000 ultrafast bootstrap replications (Hoang et al., 2018) for a dataset encompassing sequences from both subfamilies. Considering the reciprocal monophyly of each subfamily (Figure S1), we proceeded to construct separate trees for each subfamily.

### Function and substrate prediction 

To investigate the OMT proteins’ putative functions and substrates, we used the principle that phylogenetically related genes often share similar functions and act on similar substrates (Lu et al., 2022). For this, we downloaded plant sequences for 35 functionally characterized CCoAOMTs and 75 COMTs (Table S2) from UniProt (2023). We only included sequences that have been manually reviewed by Swiss-Prot. Based on these 110 sequences, we constructed phylogenetic trees using IQ-TREE to predict their functions and substrates through phylogenetic clustering.

### Gene structure and chromosomal location

We calculated proteins’ molecular weight and isoelectric point with the ProtParam tool of Expasy (2023). To understand the intron-exon organization of OMT genes, we created a neighbor-joining tree from the six Solanaceae genera with MEGA11 (Tamura et al., 2021) and 1000 bootstrap replicates. To visualize the gene structure, we retrieved the GFF file of each species and used the online Gene Structure Display Server (Guo et al., 2007; Hu et al., 2015). We searched for conserved motifs with MEME Suite (Bailey et al., 2015) up to a maximum number of six motifs for each subfamily. After successfully identifying the motifs, we used the PROSITE tool (2023) to predict the features of these motifs. We conducted a search with motif widths ranging from six to 60. We visualized the chromosomal location of identified OMT genes using TBtools (Chen et al., 2020), except for *D. stramonium* and *P. axillaris*, for which a chromosomal-level assembly is unavailable.

## Results

### Identification of OMT genes and preliminary phylogenetic clustering 

Our study focused on 23 species of Solanaceae, and we used HMMER to identify CCoAOMT and COMT proteins, of which we found 360 and 1001 sequences, respectively. We then applied stringent filters to exclude sequences that did not meet our criteria and included outgroup sequences, resulting in a final dataset of 273 CCoAOMT sequences and 974 COMT sequences (see Table S1 for a detailed view of the number of genes per species). Phylogenetic trees based on the CCoAOMT (Figure S2) and COMT (Figure S3) proteins revealed that sequences from the same genus tended to cluster together. Thus, to better visualize the evolutionary patterns of OMTs in Solanaceae, we selected only one representative species per genus.

### Evolutionary patterns in representative species of Solanaceae

The HMMER (2023) search identified 340 OMT protein sequences belonging to the two subfamilies through the six Solanaceae genera (Table 1). Subsequently, we filtered out sequences that lacked a Pfam domain or were too short (Table S3), keeping 57 CCoAOMT and 196 COMT sequences. Upon addition of outgroup sequences, the CCoAOMT final dataset comprised 86 CCoAOMT and 331 COMT protein sequences. The number of filtered sequences varied between 34 to 55 per representative species, with *I. cyaneum* exhibiting the highest count. 

### Phylogenetic relationships and function prediction

**Figure 1-:**
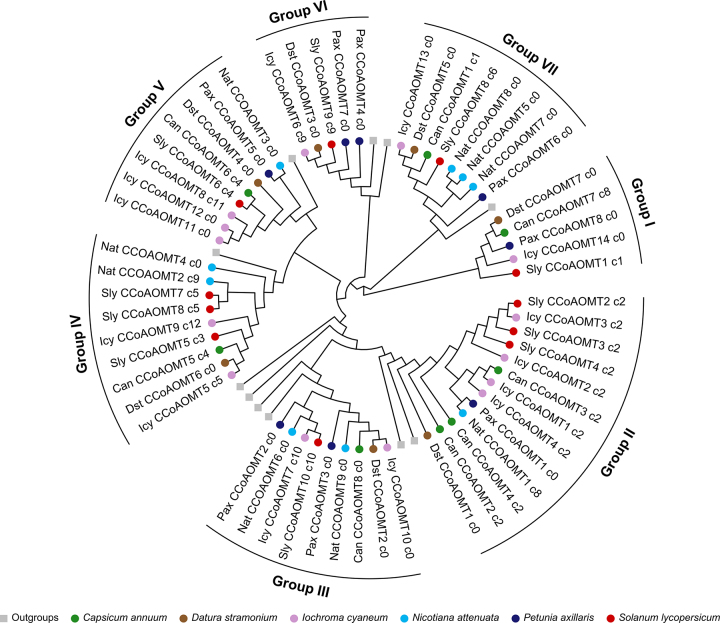
Maximum likelihood genealogies of CCoAOMT proteins from selected Solanaceae species. Outer curves indicate the identified groups. To improve visualization, clades including only outgroup sequences were collapsed. Branch tips are color-coded according to species. The tree is midpoint rooted. Gray squares represent collapsed nodes of the outgroups *Arabidopsis thaliana* (Ath), *Citrus sinensis* (Csi), *Populus trichocarpa* (Ptr), and *Vitis vinifera* (Vvi).

To analyze the evolutionary relationships and duplication/loss patterns of the OMT genes, we constructed a maximum likelihood tree using protein sequences for each subfamily and divided the tree into monophyletic subgroups for results’ better visualization. The CCoAOMT subfamily tree (Figure 1) revealed that each group presented orthologs of all six species, except group IV, which lacks *P. axillaris* genes, and groups I and VI, which lack *N. attenuata* genes. Such absence of the sequences must be taken with caution as it could be the result of issues during genome assembly, gene annotation, or failure to meet our screening criteria (e.g., minimum sequence length and motif presence). Notably, our analysis also highlights gene duplication in particular clades. For instance, in group II, we observe several CCoAOMT copies in *C. annuum*, *I. cyaneum, and S. lycopersicum*, with all copies located in chromosome (chr) 2 in these three species.

**Figure 2-:**
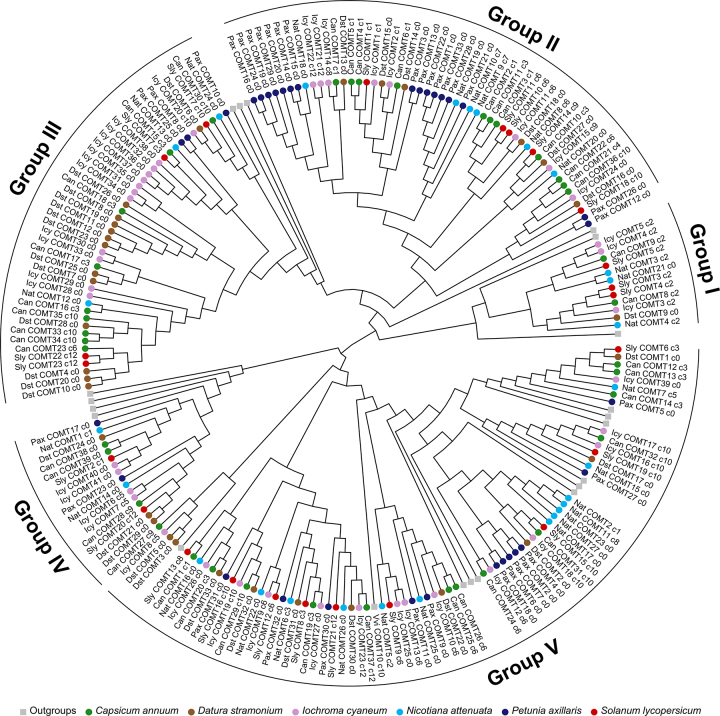
Maximum likelihood genealogies of COMT proteins from selected Solanaceae species. Outer curves indicate the identified groups. To improve visualization, clades including only outgroup sequences were collapsed. Branch tips are color-coded according to species. The tree is midpoint rooted. Gray squares represent collapsed nodes of the outgroups *Arabidopsis thaliana* (Ath), *Citrus sinensis* (Csi), *Populus trichocarpa* (Ptr), and *Vitis vinifera* (Vvi).

The COMT subfamily tree (Figure 2) showed a similar pattern to that observed in the CCoAOMT subfamily tree. Most groups had at least one representative of all genera, indicating relatively few loss events throughout evolutionary history. However, we also observed evidence of numerous recent gene duplications in species of different groups, such as *P. axillaris* in groups II and V, *I. cyaneum* in group III, and *N. attenuata* in group V. 

The phylogenetic tree containing the Solanaceae CCoAOMT proteins and the functionally characterized proteins (Figure S4) revealed that all identified groups clustered with proteins involved in methylation and lignin synthesis (Table S4). The clades showed that these proteins are involved in metal ion binding and SAM-dependent methyltransferase activity in addition to the caffeoyl-CoA O-methyltransferase activity, as the name of the subfamily suggests. Regarding the biological function of CCoAOMTs in Solanaceae, groups II and III would be potentially involved in circadian rhythm, leaf volatile biosynthesis, and phenylpropanoid metabolism. In turn, group VI encompassed the proteins associated with the highest diversity of biological processes, including seed development, pigmentation, and biosynthesis of cyanidin, delphinidin, and spermidine hydroxycinnamate.

The COMT subfamily is expected to have a broader range of biological and molecular functions and act on a greater variety of substrates due to its larger number of genes (Figure S5; Table S4). The phylogenetic clustering with functionally characterized proteins showed that all COMT groups of Solanaceae would be involved in methylation and alkaloid metabolism and biosynthesis. However, lignin biosynthesis-one of the most extensively studied processes of the OMT family-was only associated with groups IV and V of the COMT subfamily. Moreover, our findings showed that, in Solanaceae, these proteins might respond to adverse conditions such as wounds, cold, and high light intensity, as well as metabolic processes involving phenylpropanoids and isoflavonoids. Group V, which can act on several substrates, had the highest number of biological functions, including response to phytohormones such as salicylic acid, ethylene, and jasmonic acid.

### Gene structure and chromosomal location 

After removing the short sequences, the CCoAOMT length of the six Solanaceae species varied from 185 to 685 aa, whereas COMT length ranged from 204 to 888 aa (Table S3). The number of introns also varied greatly within the subfamilies: zero to 16 in CCoAOMT (Figure S6) and zero to 12 in COMT (Figure S7). The MEME (2023) search for conserved motifs revealed that most CCoAOMT genes have the six conserved motifs (Figure S6), except for some proteins that seem to have undergone duplication (i.e., motifs are present twice in the protein) and four sequences that have low or no probability of having a motif. The motifs in the COMT subfamily follow a similar pattern (Figure S7), where most motifs were conserved in all proteins, except for a clade where these motifs were not found. In the CCoAOMT proteins, PROSITE tool (2023) identified binding sites for SAM ligands in motifs 1, 2, and 4. Additionally, motif 2 exhibited binding sites for divalent metal cations. Similarly, the COMT proteins displayed a similar pattern, with motifs 1, 2, and 5 showing binding sites for SAM, and motif 1 exhibiting one active site as a proton acceptor.

**Figure 3-:**
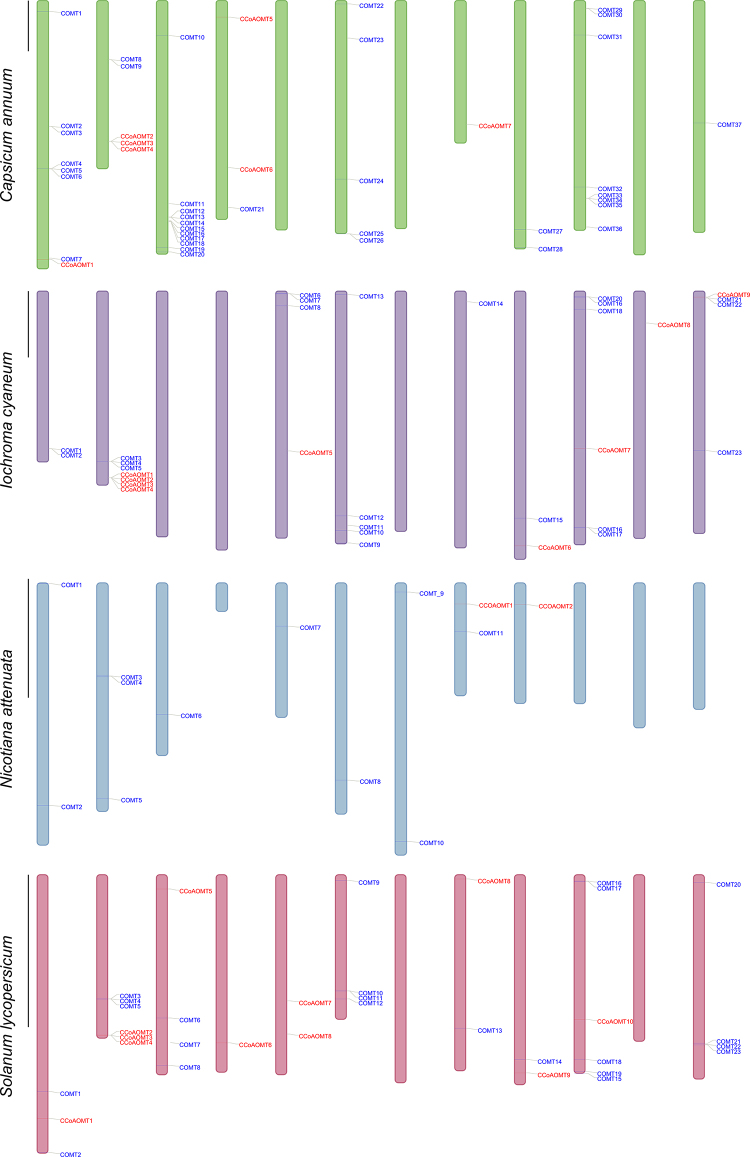
Chromosomal distribution of OMT genes in *Capsicum annuum*, *Iochroma cyaneum*, *Nicotiana attenuata*, and *Solanum lycopersicum*. Chromosomes are ordered by their corresponding number in ascending order from left to right. Scale bar = 50 megabases (Mb).

To gain insights into duplication events, we examined the chromosomal location of CCoAOMT and COMT genes in *I. cyaneum, C. annuum, N. attenuata, and S. lycopersicum* (Figure 3), in which genome assemblies at the chromosomal level are available. However, even with highly advanced genome assembly techniques, some genomic regions may not be confidently assigned to a specific chromosome and instead are placed in scaffolds. As a result, we could not assign a chromosomal location for 23 genes from *I. cyaneum*, three genes from *C. annuum*, and 23 genes from *N. attenuata*. The CCoAOMT genes of *C. annuum* were distributed among four chromosomes, with chr2 containing the highest copy number. On the other hand, COMT genes were found more widely distributed throughout the genome, and chr3 seems to have undergone multiple duplications, with 10 COMT genes located near each other. In *S. lycopersicum*, CCoAOMT and COMT were both present in eight of the 12 chromosomes. Notably, chr2 has duplicated in the CCoAOMT and COMT genes. Inferences about the chromosomal location of *I. cyaneum* and *N. attenuata* should be made cautiously as 23 genes were not assembled at the chromosome level for both species. However, we observed that chr2 of *I. cyaneum* underwent tandem duplication for CCoAOMT and COMT genes.

## Discussion

The OMT gene family plays a crucial role in plants, as it is involved in the biosynthesis of lignin and other secondary metabolites important for many biological processes (Ibrahim et al., 1998; Lam et al., 2007; Balasubramani et al., 2021). In this study, we investigated the evolution and diversification of this gene family in Solanaceae. Based on a preliminary phylogenetic analysis of OMT proteins for 23 Solanaceae species (Figure S2 and S3), we found that sequences from congeneric species overall clustered together, suggesting that the phylogenetic signal is generally constrained at the genus level. Recent studies analyzing the evolutive stories of other gene families in Solanaceae found a similar pattern (Pereira et al., 2022; Thompson et al., 2023). Thus, we only kept one representative species of each genus to better visualize the evolutionary history of the OMT gene family in Solanaceae.

OMT gene count showed wide variation among Solanaceae species, with no clear trend of higher gene counts in a particular genus. Considering genera with multiple sampled species, *Nicotiana* displayed 36 to 58 genes, whereas *Solanum* exhibited 27 to 71 genes, indicating a notable fluctuation in gene count per plant species in the same genus. Additionally, the number of COMT genes exceeded that of CCoAOMT genes in all Solanaceae studied species, similar to what was found in other plant species (Barakat et al., 2011; Liu et al., 2016; Hafeez et al., 2021; Lu et al., 2022). We did not observe any association between the number of genes and polyploidy, as exemplified by *N. tabacum*, an allotetraploid plant resulting from hybridization between *N. sylvestris* and *N. tomentosiformis* (Leitch et al., 2008). Despite having a larger genome than its parental species (Edwards et al., 2017), the number of OMT genes in *N. tabacum* is higher but not equal to the sum of its parental species (Table S1). The higher copy number can be explained by the relaxation of purifying selection and the putative functional redundancy of these genes on duplicated genomes, which can lead to gene loss (Langham et al., 2004; Cheng et al., 2018). 

Whereas the number of introns varied within both subfamilies, the genes’ structure exhibited a remarkable degree of motif conservation, except for one cluster in each subfamily that lacked these conserved motifs. This loss suggests that the proteins encoded by these clusters may have been selected to perform a different function or changed the original function via pseudogenization (Garewal et al., 2021). Three conserved motifs of CCoAOMT exhibited affinity towards SAM and one specific motif contained a binding site dedicated to metal cations. The affinity to metal ions is characteristic of CCoAOMT, because their role as a cofactor in transferring the methyl group to substrates (e.g., Hugueney et al., 2009; Xu et al., 2015). Similarly, three COMT motifs showed affinity toward SAM, and one motif was predicted as a proton acceptor. The proton acceptor plays a critical role in COMT-mediated methylation by deprotonating the target substrate and enhancing its reactivity toward the methyl group of SAM (Abdelraheem et al., 2022). The molecular weights of the proteins in each subfamily were broadly consistent with their expected ranges, which were reported as 26 to 30 kDa for CCoAOMT and 40 to 43 kDa for COMT (Kim et al., 2010), with some exceptions to this general trend.

The diversification of OMT genes in plants can be traced back to the ancestor of all land plants in which an early duplication event occurred, giving rise to two major OMT groups characterized by their distinct substrate affinities (Lam et al., 2007; Barakat et al., 2011). Subsequent duplications have contributed to the expansion and diversification of OMTs in plants, involving three primary duplication mechanisms: whole genome duplication, segmental duplication, and tandem duplication.

Whole genome duplication with subsequent genome rearrangement is essential in gene family diversification (Clark and Donoghue, 2018). In Solanaceae, two whole genome triplications might be involved in expanding the OMT gene family: the first is shared with all eudicots, and the second is more recent and occurred in the ancestor of Solanaceae (The Tomato Genome Consortium, 2012; Huang et al., 2022). Besides whole genome duplication, segmental duplicantion - in which gene copies are created across the genome because regions of the genome are duplicated to the same or a different chromosome (Bailey et al., 2002; Panchy et al., 2016) - is another main mechanism of OMT gene family expansion, as seen here in Solanaceae, *Citrus* (Liu et al., 2016), and *Vitis* (Lu et al., 2022). Segmental duplications are usually associated with repetitive sequences and transposable elements (Panchy *et al*., 2016), but the underlying mechanisms of such processes remain poorly understood. Further research is needed to elucidate these mechanisms and their potential functional implications.

Another important mechanism involved in gene family expansion is tandem duplications - when the resulting copies are adjacent to each other on the same chromosome (Jander and Barth, 2007). Here, we observed that the tandem duplication magnitude varied among species (Figure 3), with *N. attenuata* showing the lowest level of tandem duplications. Still, we should look at this result carefully as many genes are not assigned to a specific chromosome and thus are not displayed in Figure 3. Three species showed a tandem-duplicated CCoAOMT region on chr2 and, due to the species phylogenetic relatedness and the chr2 synteny (The Tomato Genome Consortium, 2012), such duplication could represent an ancestral event that has been maintained in these species. The number of tandem duplication events in Solanaceae seems to be ancient, not as abundant as in other genera [e.g., *Populus* (Barakat et al., 2011), *Arabidopsis* (Barakat *et al*., 2011), and *Citrus* (Liu et al., 2016)], and agrees with previous ideas that Solanaceae tend to have a lower number of tandem duplicated regions than species that have not gone through multiple and recent whole genome duplications (Huang et al., 2022). The observed expansion and diversification of the OMT gene family might be a combination of different processes at different time points that is a typical pattern in Solanaceae (The Tomato Genome Consortium, 2012), as well as in distantly-related species (e.g., Hofberger et al., 2015; Kuo et al., 2019; Zafar et al., 2022).

Subfunctionalization and neofunctionalization play critical roles in the retention and diversification of genes that have undergone duplication, leading to functional divergence between copies (Pichersky and Gang, 2000; Han et al., 2007). This evolutionary phenomenon can be illustrated with OMTs regarding anthocyanin biosynthesis, a well-studied metabolic pathway involving these genes. Anthocyanins are pigments that confer red and purple colors to flowers, fruits, and leaves. OMT enzymes catalyze the transfer of a methyl group to the hydroxyl group of anthocyanidins, resulting in the formation of methylated anthocyanins such as petunidin and peonidin, which are stable and water-soluble pigments (Ma et al., 2021). Our functional phylogenetic clustering showed that CCoAOMT group VI proteins are associated with anthocyanin biosynthesis, and their phylogenetic position implies neofunctionalization from an ancestral gene duplication event. *Nicotiana attenuata* and *C. annuum* are absent in group VI, indicating gene loss in those species. Moreover, the independent origin of floral volatile production in CCoAOMT groups II and III underscores the high plasticity of this gene family. Such plasticity illustrates how OMT genes may affect pollinator interaction and contribute to rapid speciation events. 

There are more genes in the COMT than in the CCoAOMT subfamily. This larger gene set is expected to translate into a broader range of substrates that COMT proteins can methylate and, consequently, into a more diverse array of biological and molecular processes in which they could be involved, highlighting the versatility and adaptability of the COMT subfamily (Lam et al., 2007). Notably, some of these genes were identified as putatively involved in stress response mechanisms, including those triggered by cold (groups I, II, and III), wounding (groups I and V), and high light intensity (groups I, II, and III). In other plants, these genes have been reported to be involved in response to abiotic and biotic factors (e.g., Hafeez et al., 2021; Zhao et al., 2022). Whereas these findings do not definitively establish the function and substrate of these proteins, we emphasize that they offer valuable insights into the evolutionary mechanisms underlying the diverse functions observed in Solanaceae. Further functional verifications are necessary to confirm the precise role of each protein, although our results serve as an initial step toward unraveling the potential of COMT proteins in adaptation to stressful conditions in Solanaceae.

In our study, we thoroughly examined the evolution and diversification of OMT genes in Solanaceae. Our findings shed light on the intricate mechanisms that drive the evolutionary process of this gene family. The results also corroborate the significant impact of OMT genes on plant interaction with biotic and abiotic factors, making them ecologically significant and potential targets for genetic engineering to improve agronomic traits. Finally, further research is necessary to expand our knowledge about OMT functions and potential applications in agriculture and biotechnology.

## Supplementary material

The following online material is available for this article:

Table S1 - Gene count of raw and filtered datasets, as well as the reference for all 23 Solanaceae species.

Table S2 - Functionally characterized OMT proteins downloaded from UniProt.

Table S3 - Structural and molecular characteristics of OMT proteins of six Solanaceae representative species.

Table S4 - Putative substrate, biological and molecular processes associated with the phylogenetic groups of the OMT family of Solanaceae species.

Figure S1 - Maximum likelihood genealogy of the complete dataset shows the reciprocal monophyly of both CCoAOMT and COMT subfamilies.

Figure S2 - Maximum likelihood genealogy of CCoAOMT proteins from a comprehensive dataset including 23 Solanaceae species.

Figure S3 - Maximum likelihood genealogy of CCoAOMT proteins from a comprehensive dataset including 23 Solanaceae species.

Figure S4 - Maximum likelihood genealogy of CCoAOMT proteins from the function dataset, including the six Solanaceae species and the 35 functionally characterized proteins from UniProt.

Figure S5 - Maximum likelihood genealogy of COMT proteins from the function dataset, including the six Solanaceae species and the 75 functionally characterized proteins from UniProt.

Figure S6 - Gene structure and protein motif composition of CCoAOMT proteins from selected Solanaceae species. Introns were rescaled to the same length.

Figure S7 - Gene structure and protein motif composition of COMT proteins from selected Solanaceae species.
