# Supplementary material for: Evolution and diversification of the O-methyltransferase (OMT) gene family in Solanaceae
Source: Genet Mol Biol. 2023 Nov 10;46(3 Suppl 1):e20230121. doi: 10.1590/1678-4685-GMB-2023-0121 (PMC10637433; doi:10.1590/1678-4685-GMB-2023-0121)
Supplement: Table S1 - [file 1415-4757-GMB-46-3-s1-e20230121-s1.pdf]

Genetics and Molecular Biology
https://doi.org/10.1590/1678-4685-GMB-2023-0121

Supplementary Material to "Evolution and diversification of the O-methyltransferase (OMT) gene family in Solanaceae"

Table S1 - Gene count of raw and filtered datasets, as well as the reference for all 23 Solanaceae species.

	Raw Data			Filtered Data			
**Species**	**CCoAOMT**	**COMT**	**Total**	**CCoAOMT**	**COMT**	**Total**	**Reference**
Capsicum annuum	11	64	75	8	39	47	SolGenomics
Capsicum baccatum	14	74	88	8	56	64	SAMN04417068
Capsicum chinense	12	78	90	10	52	62	SAMN05435899
Datura stramonium	12	41	53	7	33	40	SAMN14375310
Iochroma cyaneum	16	46	62	14	41	55	SolGenomics
Nicotiana attenuata	10	29	39	9	27	36	SolGenomics
Nicotiana benthamiana	13	37	50	9	35	44	SolGenomics
Nicotiana sylvestris	13	33	46	11	30	41	SAMN02981529
Nicotiana tabacum	22	58	80	10	48	58	SolGenomics
Nicotiana tomentosiformis	12	30	42	12	28	40	SAMN02981530
Petunia axillaris	11	39	50	8	33	41	SolGenomics
Petunia inflata	16	48	64	13	43	56	SolGenomics
Solanum chilense	11	23	34	8	19	27	SolGenomics
Solanum commersonii	14	51	65	12	46	58	SAMN15755581
Solanum lycopersicoides	14	48	62	13	47	60	SolGenomics
Solanum lycopersicum	29	32	61	11	23	34	SolGenomics
Solanum lycopersicum var. *cerasiforme*	33	26	59	12	22	34	SolGenomics
Solanum melongena	11	32	43	8	27	35	SolGenomics
Solanum pennellii	12	31	43	9	28	37	SolGenomics
Solanum pimpinellifolium	22	32	54	9	27	36	SolGenomics
Solanum stenotomum	18	53	71	18	53	71	SAMN14480720
Solanum tuberosum	17	60	77	8	46	54	SolGenomics
Solanum verrucosum	17	36	53	17	36	53	SAMEA104077226
Arabidopsis thaliana	-	-	-	7	17	24	ENSEMBL Plants
Vitis vinifera	-	-	-	10	37	47	ENSEMBL Plants
Citrus sinensis	-	-	-	6	52	58	Citrus Pan-genome to Breeding Database
Populus trichocarpa	-	-	-	6	29	35	ENSEMBL Plants
**Total**	**360**	**1001**	**1361**	**273**	**974**	**1083**	-
