# Supplementary material for: Evolution and diversification of the O-methyltransferase (OMT) gene family in Solanaceae
Source: Genet Mol Biol. 2023 Nov 10;46(3 Suppl 1):e20230121. doi: 10.1590/1678-4685-GMB-2023-0121 (PMC10637433; doi:10.1590/1678-4685-GMB-2023-0121)
Supplement: Table S2 - [file 1415-4757-GMB-46-3-s1-e20230121-s2.pdf]

Genetics and Molecular Biology
https://doi.org/10.1590/1678-4685-GMB-2023-0121

Supplementary Material to "Evolution and diversification of the O-methyltransferase (OMT) gene family in Solanaceae"

Table S2 - Functionally characterized OMT proteins downloaded from UniProt.

Group	Protein families	Entry	Organism	Catalytic activity	Pathway	Tissue specificity	Gene Ontology (biological process)	Gene Ontology (molecular function)	Function
II	CCoAMT	A0A0S2UWT1	*Petunia hybrida* (Petunia)	(E)-caffeoyl-CoA + S-adenosyl-L-methionine = (E)-feruloyl-CoA + H(+) + S-adenosyl-L-homocysteine	Aromatic compound metabolism; Phenylpropanoid biosynthesis.	Mostly expressed in petal limbs and tubes, and, at low levels, in stems, roots and leaves.	circadian rhythm [GO:0007623]; green leaf volatile biosynthetic process [GO:0010597]; lignin biosynthetic process [GO:0009809]; methylation [GO:0032259]; phenylpropanoid metabolic process [GO:0009698]	caffeoyl CoA:S-adenosyl-L-methionine O-methyltransferase activity [GO:0080076]; caffeoyl-CoA O-methyltransferase activity [GO:0042409]; metal ion binding [GO:0046872]	Involved in the production of floral volatile phenylpropanoids in flowers of fragrant cultivars (e.g. cv. Mitchell and cv. V26) from cinnamic acid, a common precursor with the anthocyanin biosynthesis pathway involved in flower pigmentation (By similarity). Methylates caffeoyl-CoA to feruloyl-CoA, also able to methylate 5-hydroxyferuloyl-CoA.
II	CCoAMT	O04854	*Eucalyptus gunnii* (Cider gum)	(E)-caffeoyl-CoA + S-adenosyl-L-methionine = (E)-feruloyl-CoA + H(+) + S-adenosyl-L-homocysteine	Aromatic compound metabolism; Phenylpropanoid biosynthesis.		lignin biosynthetic process [GO:0009809]; methylation [GO:0032259]	caffeoyl-CoA O-methyltransferase activity [GO:0042409]; metal ion binding [GO:0046872]	Methylates caffeoyl-CoA to feruloyl-CoA and 5-hydroxyferuloyl-CoA to sinapoyl-CoA. Plays a role in the synthesis of feruloylated polysaccharides. Involved in the reinforcement of the plant cell wall. Also involved in the responding to wounding or pathogen challenge by the increased formation of cell wall-bound ferulic acid polymers.
II	CCoAMT	O24144	*Nicotiana tabacum* (Common tobacco)	(E)-caffeoyl-CoA + S-adenosyl-L-methionine = (E)-feruloyl-CoA + H(+) + S-adenosyl-L-homocysteine	Aromatic compound metabolism; Phenylpropanoid biosynthesis.	Mostly expressed in the bottom and middle parts of the stems.	lignin biosynthetic process [GO:0009809]; methylation [GO:0032259]	caffeoyl-CoA O-methyltransferase activity [GO:0042409]; metal ion binding [GO:0046872]; S-adenosylmethionine-dependent methyltransferase activity [GO:0008757]	Methylates caffeoyl-CoA to feruloyl-CoA and 5-hydroxyferuloyl-CoA to sinapoyl-CoA. Plays a role in the synthesis of feruloylated polysaccharides. Involved in the reinforcement of the plant cell wall. Also involved in the responding to wounding or pathogen challenge by the increased formation of cell wall-bound ferulic acid polymers.
II	CCoAMT	O24149	*Nicotiana tabacum* (Common tobacco)	(E)-caffeoyl-CoA + S-adenosyl-L-methionine = (E)-feruloyl-CoA +	Aromatic compound metabolism;	Mostly expressed in the bottom and middle	lignin biosynthetic process [GO:0009809];	caffeoyl-CoA O-methyltransferase activity [GO:0042409]; metal	Methylates caffeoyl-CoA to feruloyl-CoA and 5-hydroxyferuloyl-CoA to sinapoyl-CoA. Plays a role in the synthesis of feruloylated

Group	Protein families	Entry	Organism	Catalytic activity	Pathway	Tissue specificity	Gene Ontology (biological process)	Gene Ontology (molecular function)	Function
				H(+) + S-adenosyl-L-homocysteine	Phenylpropanoid biosynthesis.	parts of the stems.	methylation [GO:0032259]	ion binding [GO:0046872]; S-adenosylmethionine-dependent methyltransferase activity [GO:0008757]	polysaccharides. Involved in the reinforcement of the plant cell wall. Also involved in the responding to wounding or pathogen challenge by the increased formation of cell wall-bound ferulic acid polymers. Methylates 5-hydroxyferulolyl-CoA more efficiently than caffeoyl-CoA.
II	CCoAMT	O24150	*Nicotiana tabacum* (Common tobacco)	(E)-caffeoyl-CoA + S-adenosyl-L-methionine = (E)-feruloyl-CoA + H(+) + S-adenosyl-L-homocysteine	Aromatic compound metabolism; Phenylpropanoid biosynthesis.	Mostly expressed in the bottom and middle parts of the stems.	lignin biosynthetic process [GO:0009809]; methylation [GO:0032259]	caffeoyl-CoA O-methyltransferase activity [GO:0042409]; metal ion binding [GO:0046872]; S-adenosylmethionine-dependent methyltransferase activity [GO:0008757]	Methylates caffeoyl-CoA to feruloyl-CoA and 5-hydroxyferuloyl-CoA to sinapoyl-CoA. Plays a role in the synthesis of feruloylated polysaccharides. Involved in the reinforcement of the plant cell wall. Also involved in the responding to wounding or pathogen challenge by the increased formation of cell wall-bound ferulic acid polymers. Methylates also free caffeic and 5-hydroxyferulic acids.
II	CCoAMT	O24151	*Nicotiana tabacum* (Common tobacco)	(E)-caffeoyl-CoA + S-adenosyl-L-methionine = (E)-feruloyl-CoA + H(+) + S-adenosyl-L-homocysteine	Aromatic compound metabolism; Phenylpropanoid biosynthesis.	Mostly expressed in the bottom and middle parts of the stems.	lignin biosynthetic process [GO:0009809]; methylation [GO:0032259]	caffeoyl-CoA O-methyltransferase activity [GO:0042409]; metal ion binding [GO:0046872]; S-adenosylmethionine-dependent methyltransferase activity [GO:0008757]	Methylates caffeoyl-CoA to feruloyl-CoA and 5-hydroxyferuloyl-CoA to sinapoyl-CoA. Plays a role in the synthesis of feruloylated polysaccharides. Involved in the reinforcement of the plant cell wall. Also involved in the responding to wounding or pathogen challenge by the increased formation of cell wall-bound ferulic acid polymers.
II	CCoAMT	O49499	*Arabidopsis thaliana* (Mouse-ear cress)	(E)-caffeoyl-CoA + S-adenosyl-L-methionine = (E)-feruloyl-CoA + H(+) + S-adenosyl-L-homocysteine	Aromatic compound metabolism; Phenylpropanoid biosynthesis.	Expressed in stems and roots. Detected in leaves, siliques, flower buds, flowers. Expressed in the tapetum, but not in the endothecium. Detected in the vascular system of leaves and all flower organs, including stigma, stamens, petals and sepals.	coumarin biosynthetic process [GO:0009805]; lignin biosynthetic process [GO:0009809]; methylation [GO:0032259]	caffeoyl-CoA O-methyltransferase activity [GO:0042409]; metal ion binding [GO:0046872]; S-adenosylmethionine-dependent methyltransferase activity [GO:0008757]	Methylates caffeoyl-CoA to feruloyl-CoA. Has a very low activity with caffeic acid and esculetin. Involved in scopoletin biosynthesis in roots.

Group	Protein families	Entry	Organism	Catalytic activity	Pathway	Tissue specificity	Gene Ontology (biological process)	Gene Ontology (molecular function)	Function
II	CCoAMT	O65862	*Populus trichocarpa* (Western balsam poplar) (*Populus balsamifera* subsp. *trichocarpa*)	(E)-caffeoyl-CoA + S-adenosyl-L-methionine = (E)-feruloyl-CoA + H(+) + S-adenosyl-L-homocysteine	Aromatic compound metabolism; Phenylpropanoid biosynthesis.		lignin biosynthetic process [GO:0009809]; methylation [GO:0032259]	caffeoyl-CoA O-methyltransferase activity [GO:0042409]; metal ion binding [GO:0046872]	Methylates caffeoyl-CoA to feruloyl-CoA and 5-hydroxyferuloyl-CoA to sinapoyl-CoA. Plays a role in the synthesis of feruloylated polysaccharides. Involved in the reinforcement of the plant cell wall. Also involved in the responding to wounding or pathogen challenge by the increased formation of cell wall-bound ferulic acid polymers.
II	CCoAMT	O65922	*Populus trichocarpa* (Western balsam poplar) (*Populus balsamifera* subsp. *trichocarpa*)	(E)-caffeoyl-CoA + S-adenosyl-L-methionine = (E)-feruloyl-CoA + H(+) + S-adenosyl-L-homocysteine	Aromatic compound metabolism; Phenylpropanoid biosynthesis.		lignin biosynthetic process [GO:0009809]; methylation [GO:0032259]	caffeoyl-CoA O-methyltransferase activity [GO:0042409]; metal ion binding [GO:0046872]	Methylates caffeoyl-CoA to feruloyl-CoA and 5-hydroxyferuloyl-CoA to sinapoyl-CoA. Plays a role in the synthesis of feruloylated polysaccharides. Involved in the reinforcement of the plant cell wall. Also involved in the responding to wounding or pathogen challenge by the increased formation of cell wall-bound ferulic acid polymers.
II	CCoAMT	O81185	*Eucalyptus globulus* (Tasmanian blue gum)	(E)-caffeoyl-CoA + S-adenosyl-L-methionine = (E)-feruloyl-CoA + H(+) + S-adenosyl-L-homocysteine	Aromatic compound metabolism; Phenylpropanoid biosynthesis.		lignin biosynthetic process [GO:0009809]; methylation [GO:0032259]	caffeoyl-CoA O-methyltransferase activity [GO:0042409]; metal ion binding [GO:0046872]	Methylates caffeoyl-CoA to feruloyl-CoA and 5-hydroxyferuloyl-CoA to sinapoyl-CoA. Plays a role in the synthesis of feruloylated polysaccharides. Involved in the reinforcement of the plant cell wall. Also involved in the responding to wounding or pathogen challenge by the increased formation of cell wall-bound ferulic acid polymers.
II	CCoAMT	P28034	*Petroselinum crispum* (Parsley) (*Petroselinum hortense*)	(E)-caffeoyl-CoA + S-adenosyl-L-methionine = (E)-feruloyl-CoA + H(+) + S-adenosyl-L-homocysteine	Aromatic compound metabolism; Phenylpropanoid biosynthesis.	Roots and leaves.	lignin biosynthetic process [GO:0009809]; methylation [GO:0032259]	caffeoyl-CoA O-methyltransferase activity [GO:0042409]; metal ion binding [GO:0046872]	Methylates caffeoyl-CoA to feruloyl-CoA and 5-hydroxyferuloyl-CoA to sinapoyl-CoA. Plays a role in the synthesis of feruloylated polysaccharides. Involved in the reinforcement of the plant cell wall. Also involved in the responding to wounding or pathogen challenge by the increased formation of cell wall-bound ferulic acid polymers.
II	CCoAMT	Q40313	*Medicago sativa* (Alfalfa)	(E)-caffeoyl-CoA + S-adenosyl-L-methionine = (E)-feruloyl-CoA + H(+) + S-adenosyl-L-homocysteine	Aromatic compound metabolism; Phenylpropanoid biosynthesis.		lignin biosynthetic process [GO:0009809]; methylation [GO:0032259]	caffeoyl-CoA O-methyltransferase activity [GO:0042409]; metal ion binding [GO:0046872]	Methylates caffeoyl-CoA to feruloyl-CoA and 5-hydroxyferuloyl-CoA to sinapoyl-CoA. Plays a role in the synthesis of feruloylated polysaccharides. Involved in the reinforcement of the plant cell wall. Also involved in the responding to wounding or pathogen challenge by the increased formation of cell wall-bound ferulic acid polymers.

Group	Protein families	Entry	Organism	Catalytic activity	Pathway	Tissue specificity	Gene Ontology (biological process)	Gene Ontology (molecular function)	Function
II	CCoAMT	Q43095	*Populus tremuloides* (Quaking aspen)	(E)-caffeoyl-CoA + S-adenosyl-L-methionine = (E)-feruloyl-CoA + H(+) + S-adenosyl-L-homocysteine	Aromatic compound metabolism; Phenylpropanoid biosynthesis.		lignin biosynthetic process [GO:0009809]; methylation [GO:0032259]	caffeoyl-CoA O-methyltransferase activity [GO:0042409]; metal ion binding [GO:0046872]	Methylates caffeoyl-CoA to feruloyl-CoA and 5-hydroxyferuloyl-CoA to sinapoyl-CoA. Plays a role in the synthesis of feruloylated polysaccharides. Involved in the reinforcement of the plant cell wall. Also involved in the responding to wounding or pathogen challenge by the increased formation of cell wall-bound ferulic acid polymers.
II	CCoAMT	Q43237	*Vitis vinifera* (Grape)	(E)-caffeoyl-CoA + S-adenosyl-L-methionine = (E)-feruloyl-CoA + H(+) + S-adenosyl-L-homocysteine	Aromatic compound metabolism; Phenylpropanoid biosynthesis.		lignin biosynthetic process [GO:0009809]; methylation [GO:0032259]	caffeoyl-CoA O-methyltransferase activity [GO:0042409]; metal ion binding [GO:0046872]	Methylates caffeoyl-CoA to feruloyl-CoA and 5-hydroxyferuloyl-CoA to sinapoyl-CoA. Plays a role in the synthesis of feruloylated polysaccharides. Involved in the reinforcement of the plant cell wall. Also involved in the responding to wounding or pathogen challenge by the increased formation of cell wall-bound ferulic acid polymers.
II	CCoAMT	Q8H9B6	*Solanum tuberosum* (Potato)	(E)-caffeoyl-CoA + S-adenosyl-L-methionine = (E)-feruloyl-CoA + H(+) + S-adenosyl-L-homocysteine	Aromatic compound metabolism; Phenylpropanoid biosynthesis.		lignin biosynthetic process [GO:0009809]; methylation [GO:0032259]	caffeoyl-CoA O-methyltransferase activity [GO:0042409]; metal ion binding [GO:0046872]; S-adenosylmethionine-dependent methyltransferase activity [GO:0008757]	Methylates caffeoyl-CoA to feruloyl-CoA and 5-hydroxyferuloyl-CoA to sinapoyl-CoA. Plays a role in the synthesis of feruloylated polysaccharides. Involved in the reinforcement of the plant cell wall. Also involved in the responding to wounding or pathogen challenge by the increased formation of cell wall-bound ferulic acid polymers.
III	CCoAMT	A0A0S2UWA5	*Petunia hybrida* (Petunia)	(E)-caffeoyl-CoA + S-adenosyl-L-methionine = (E)-feruloyl-CoA + H(+) + S-adenosyl-L-homocysteine	Aromatic compound metabolism; Phenylpropanoid biosynthesis.	Mostly expressed in petal limbs and tubes, and, at low levels, in stems, roots and leaves.	circadian rhythm [GO:0007623]; lignin biosynthetic process [GO:0009809]; methylation [GO:0032259]; phenylpropanoid metabolic process [GO:0009698]	caffeoyl CoA:S-adenosyl-L-methionine O-methyltransferase activity [GO:0080076]; caffeoyl-CoA O-methyltransferase activity [GO:0042409]; metal ion binding [GO:0046872]	Involved in the production of floral volatile phenylpropanoids in flowers of fragrant cultivars (e.g. cv. Mitchell and cv. V26) from cinnamic acid, a common precursor with the anthocyanin biosynthesis pathway involved in flower pigmentation (By similarity). Methylates caffeoyl-CoA to feruloyl-CoA, also able to methylate 5-hydroxyferuloyl-CoA.
III	CCoAMT	A0A0S2UWC9	*Petunia hybrida* (Petunia)	(E)-caffeoyl-CoA + S-adenosyl-L-methionine = (E)-feruloyl-CoA + H(+) + S-adenosyl-L-homocysteine	Aromatic compound metabolism; Phenylpropanoid biosynthesis.	Mostly expressed in petal limbs and tubes, and, at low levels, in flower buds, stamens, pistils, stems,	circadian rhythm [GO:0007623]; green leaf volatile biosynthetic process [GO:0010597]; lignin biosynthetic process [GO:0009809]; methylation [GO:0032259];	caffeoyl CoA:S-adenosyl-L-methionine O-methyltransferase activity [GO:0080076]; caffeoyl-CoA O-methyltransferase activity	Involved in the production of floral volatile phenylpropanoids in flowers of fragrant cultivars (e.g. cv. Mitchell and cv. V26) from cinnamic acid, a common precursor with the anthocyanin biosynthesis pathway involved in flower pigmentation. Methylates caffeoyl-CoA to feruloyl-CoA, also able to methylate 5-hydroxyferuloyl-CoA .

Group	Protein families	Entry	Organism	Catalytic activity	Pathway	Tissue specificity	Gene Ontology (biological process)	Gene Ontology (molecular function)	Function
						roots and leaves.	phenylpropanoid metabolic process [GO:0009698]	[GO:0042409]; metal ion binding [GO:0046872]	
III	CCoAMT	O04899	*Nicotiana tabacum* (Common tobacco)	(E)-caffeoyl-CoA + S-adenosyl-L-methionine = (E)-feruloyl-CoA + H(+) + S-adenosyl-L-homocysteine	Aromatic compound metabolism; Phenylpropanoid biosynthesis.	Expression steadily increases from the bottom to the top of the plant.	lignin biosynthetic process [GO:0009809]; methylation [GO:0032259]	caffeoyl-CoA O-methyltransferase activity [GO:0042409]; metal ion binding [GO:0046872]; S-adenosylmethionine-dependent methyltransferase activity [GO:0008757]	Methylates caffeoyl-CoA to feruloyl-CoA and 5-hydroxyferuloyl-CoA to sinapoyl-CoA. Plays a role in the synthesis of feruloylated polysaccharides. Involved in the reinforcement of the plant cell wall. Also involved in the responding to wounding or pathogen challenge by the increased formation of cell wall-bound ferulic acid polymers. Methylates 5-hydroxyferulolyl-CoA more efficiently than caffeoyl-CoA.
III	CCoAMT	Q42945	*Nicotiana tabacum* (Common tobacco)	(E)-caffeoyl-CoA + S-adenosyl-L-methionine = (E)-feruloyl-CoA + H(+) + S-adenosyl-L-homocysteine	Aromatic compound metabolism; Phenylpropanoid biosynthesis.	Expression increases from the bottom to the top of the plant, with a pic in the middle part of the stem.	lignin biosynthetic process [GO:0009809]; methylation [GO:0032259]	caffeoyl-CoA O-methyltransferase activity [GO:0042409]; metal ion binding [GO:0046872]; S-adenosylmethionine-dependent methyltransferase activity [GO:0008757]	Methylates caffeoyl-CoA to feruloyl-CoA and 5-hydroxyferuloyl-CoA to sinapoyl-CoA. Plays a role in the synthesis of feruloylated polysaccharides. Involved in the reinforcement of the plant cell wall. Also involved in the responding to wounding or pathogen challenge by the increased formation of cell wall-bound ferulic acid polymers. Methylates almost exclusively caffeoyl-CoA.
III	CCoAMT	Q9SLP8	*Citrus natsudaidai* (Natsudaidai orange) (Japanese summer orange)	(E)-caffeoyl-CoA + S-adenosyl-L-methionine = (E)-feruloyl-CoA + H(+) + S-adenosyl-L-homocysteine	Aromatic compound metabolism; Phenylpropanoid biosynthesis.		lignin biosynthetic process [GO:0009809]; methylation [GO:0032259]	caffeoyl-CoA O-methyltransferase activity [GO:0042409]; metal ion binding [GO:0046872]	Methylates caffeoyl-CoA to feruloyl-CoA and 5-hydroxyferuloyl-CoA to sinapoyl-CoA. Plays a role in the synthesis of feruloylated polysaccharides. Involved in the reinforcement of the plant cell wall. Also involved in the responding to wounding or pathogen challenge by the increased formation of cell wall-bound ferulic acid polymers.
VI	CCoAMT	C7AE94	*Vitis vinifera* (Grape)	S-adenosyl-L-methionine + a 3'-hydroxyflavonoid = S-adenosyl-L-homocysteine + a 3'-methoxyflavonoid	Pigment biosynthesis; Anthocyanin biosynthesis.		anthocyanin-containing compound biosynthetic process [GO:0009718]; cyanidin 3-O-glucoside biosynthetic process [GO:0033485]; delphinidin 3-O-glucoside biosynthetic process [GO:0033486]; methylation [GO:0032259];	laricitrin 5'-O-methyltransferase activity [GO:0070448]; metal ion binding [GO:0046872]; myricetin 3'-O-methyltransferase activity [GO:0033799]; O-methyltransferase activity [GO:0008171]	Mediates O-methylation of anthocyanins. Anthocyanins are major pigments in grapes: at ripening initiation in red grapevine berries, the exocarp turns color from green to red and then to purple due to the accumulation and extent of methylation of anthocyanins. Catalyzes both 3' and 5' O-methylation of anthocyanins, with a preference for glycosylated substrates. Active on both anthocyanins and flavonols in vitro. Most active with delphinidin 3-glucoside but also acts on cyanidin 3-glucoside, cyanidin, myricetin, quercetin and quercetin 3-glucoside.

Group	Protein families	Entry	Organism	Catalytic activity	Pathway	Tissue specificity	Gene Ontology (biological process)	Gene Ontology (molecular function)	Function
							pigmentation [GO:0043473]		Not able to methylate flavan type skeletons with chiral centers, such as catechins or dihydroquercetin.
VI	CCoAMT	P93711	*Populus kitakamiensis* (Aspen) (*Populus sieboldii* x *Populus grandidentata*)	(E)-caffeoyl-CoA + S-adenosyl-L-methionine = (E)-feruloyl-CoA + H(+) + S-adenosyl-L-homocysteine	Aromatic compound metabolism; Phenylpropanoid biosynthesis.		lignin biosynthetic process [GO:0009809]; methylation [GO:0032259]	caffeoyl-CoA O-methyltransferase activity [GO:0042409]; metal ion binding [GO:0046872]	Methylates caffeoyl-CoA to feruloyl-CoA and 5-hydroxyferuloyl-CoA to sinapoyl-CoA. Plays a role in the synthesis of feruloylated polysaccharides. Involved in the reinforcement of the plant cell wall. Also involved in the responding to wounding or pathogen challenge by the increased formation of cell wall-bound ferulic acid polymers.
VI	CCoAMT	Q43161	*Stellaria longipes* (Longstalk starwort) (*Alsine longipes*)	(E)-caffeoyl-CoA + S-adenosyl-L-methionine = (E)-feruloyl-CoA + H(+) + S-adenosyl-L-homocysteine	Aromatic compound metabolism; Phenylpropanoid biosynthesis.		lignin biosynthetic process [GO:0009809]; methylation [GO:0032259]	caffeoyl-CoA O-methyltransferase activity [GO:0042409]; metal ion binding [GO:0046872]	Methylates caffeoyl-CoA to feruloyl-CoA and 5-hydroxyferuloyl-CoA to sinapoyl-CoA. Plays a role in the synthesis of feruloylated polysaccharides. Involved in the reinforcement of the plant cell wall. Also involved in the responding to wounding or pathogen challenge by the increased formation of cell wall-bound ferulic acid polymers.
VI	CCoAMT	Q9C9W3	*Arabidopsis thaliana* (Mouse-ear cress)	(E)-caffeoyl-CoA + S-adenosyl-L-methionine = (E)-feruloyl-CoA + H(+) + S-adenosyl-L-homocysteine	Aromatic compound metabolism; Phenylpropanoid biosynthesis.		lignin biosynthetic process [GO:0009809]; methylation [GO:0032259]	caffeoyl-CoA O-methyltransferase activity [GO:0042409]; metal ion binding [GO:0046872]; S-adenosylmethionine-dependent methyltransferase activity [GO:0008757]	Methylates caffeoyl-CoA to feruloyl-CoA and 5-hydroxyferuloyl-CoA to sinapoyl-CoA. Plays a role in the synthesis of feruloylated polysaccharides. Involved in the reinforcement of the plant cell wall. Also involved in the responding to wounding or pathogen challenge by the increased formation of cell wall-bound ferulic acid polymers (By similarity).
VI	CCoAMT	Q9C9W4	*Arabidopsis thaliana* (Mouse-ear cress)		Aromatic compound metabolism; Phenylpropanoid biosynthesis.	Expressed in inflorescences and flower buds. Not detected in roots, leaves or stems. Located exclusively in the tapetum of developing stamen.	lignin biosynthetic process [GO:0009809]; methylation [GO:0032259]; seed development [GO:0048316]; spermidine hydroxycinnamate conjugate biosynthetic process [GO:0080088]	caffeoyl CoA:S-adenosyl-L-methionine O-methyltransferase activity [GO:0080076]; caffeoyl-CoA O-methyltransferase activity [GO:0042409]; metal ion binding [GO:0046872]; S-adenosylmethionine-dependent methyltransferase activity [GO:0008757]; tricaffeoyl	Methyltransferase involved in phenylpropanoid polyamine conjugate biosynthesis. In vivo, methylates only one of the 5-hydroxyferuloyl moieties of N1,N5,N10-tri-(hydroxyferuloyl)-spermidine, while is able in vitro to convert all three 5-hydroxyferuloyl residues to the corresponding sinapoyl moieties and to methylate caffeoyl CoA and tricaffeoyl spermidine.

Group	Protein families	Entry	Organism	Catalytic activity	Pathway	Tissue specificity	Gene Ontology (biological process)	Gene Ontology (molecular function)	Function
								spermidine:S-adenosyl-L-methionine O-methyltransferase activity [GO:0080078]; trihydroxyferuloyl spermidine O-methyltransferase activity [GO:0080012]; trihydroxyferuloyl spermidine:S-adenosyl-L-methionine O-methyltransferase activity [GO:0080077]	
Outgroup II + III	CCoAMT	O65162	*Mesembryanthemum crystallinum* (Common ice plant) (*Cryophytum crystallinum*)	(E)-caffeoyl-CoA + S-adenosyl-L-methionine = (E)-feruloyl-CoA + H(+) + S-adenosyl-L-homocysteine	Aromatic compound metabolism; Phenylpropanoid biosynthesis.		lignin biosynthetic process [GO:0009809]; methylation [GO:0032259]	caffeoyl-CoA O-methyltransferase activity [GO:0042409]; metal ion binding [GO:0046872]	Methylates caffeoyl-CoA to feruloyl-CoA and 5-hydroxyferuloyl-CoA to sinapoyl-CoA. Plays a role in the synthesis of feruloylated polysaccharides. Involved in the reinforcement of the plant cell wall. Also involved in the responding to wounding or pathogen challenge by the increased formation of cell wall-bound ferulic acid polymers.
Outgroup II + III	CCoAMT	Q41720	*Zinnia violacea* (Garden zinnia) (*Zinnia elegans*)	(E)-caffeoyl-CoA + S-adenosyl-L-methionine = (E)-feruloyl-CoA + H(+) + S-adenosyl-L-homocysteine	Aromatic compound metabolism; Phenylpropanoid biosynthesis.		lignin biosynthetic process [GO:0009809]; methylation [GO:0032259]	caffeoyl-CoA O-methyltransferase activity [GO:0042409]; metal ion binding [GO:0046872]	Methylates caffeoyl-CoA to feruloyl-CoA and 5-hydroxyferuloyl-CoA to sinapoyl-CoA. Plays a role in the synthesis of feruloylated polysaccharides. Involved in the reinforcement of the plant cell wall. Also involved in the responding to wounding or pathogen challenge by the increased formation of cell wall-bound ferulic acid polymers.
Outgroup II + III	CCoAMT	Q9SWB8	*Eucalyptus globulus* (Tasmanian blue gum)	(E)-caffeoyl-CoA + S-adenosyl-L-methionine = (E)-feruloyl-CoA + H(+) + S-adenosyl-L-homocysteine	Aromatic compound metabolism; Phenylpropanoid biosynthesis.		lignin biosynthetic process [GO:0009809]; methylation [GO:0032259]	caffeoyl-CoA O-methyltransferase activity [GO:0042409]; metal ion binding [GO:0046872]	Methylates caffeoyl-CoA to feruloyl-CoA and 5-hydroxyferuloyl-CoA to sinapoyl-CoA. Plays a role in the synthesis of feruloylated polysaccharides. Involved in the reinforcement of the plant cell wall. Also involved in the responding to wounding or pathogen challenge by the increased formation of cell wall-bound ferulic acid polymers.
Outgroup II + III	CCoAMT	Q9XGD5	*Zea mays* (Maize)	(E)-caffeoyl-CoA + S-adenosyl-L-methionine = (E)-feruloyl-CoA +	Aromatic compound metabolism;		lignin biosynthetic process [GO:0009809];	caffeoyl-CoA O-methyltransferase activity [GO:0042409]; metal	Methylates caffeoyl-CoA to feruloyl-CoA and 5-hydroxyferuloyl-CoA to sinapoyl-CoA. Plays a role in the synthesis of feruloylated

Group	Protein families	Entry	Organism	Catalytic activity	Pathway	Tissue specificity	Gene Ontology (biological process)	Gene Ontology (molecular function)	Function
				H(+) + S-adenosyl-L-homocysteine	Phenylpropanoid biosynthesis.		methylation [GO:0032259]	ion binding [GO:0046872]; S-adenosylmethionine-dependent methyltransferase activity [GO:0008757]	polysaccharides. Involved in the reinforcement of the plant cell wall. Also involved in the responding to wounding or pathogen challenge by the increased formation of cell wall-bound ferulic acid polymers.
Outgroup II + III	CCoAMT	Q9XGD6	*Zea mays* (Maize)	(E)-caffeoyl-CoA + S-adenosyl-L-methionine = (E)-feruloyl-CoA + H(+) + S-adenosyl-L-homocysteine	Aromatic compound metabolism; Phenylpropanoid biosynthesis.		lignin biosynthetic process [GO:0009809]; methylation [GO:0032259]	caffeoyl-CoA O-methyltransferase activity [GO:0042409]; metal ion binding [GO:0046872]; S-adenosylmethionine-dependent methyltransferase activity [GO:0008757]	Methylates caffeoyl-CoA to feruloyl-CoA and 5-hydroxyferuloyl-CoA to sinapoyl-CoA. Plays a role in the synthesis of feruloylated polysaccharides. Involved in the reinforcement of the plant cell wall. Also involved in the responding to wounding or pathogen challenge by the increased formation of cell wall-bound ferulic acid polymers.
Outgroup II + III	CCoAMT	Q9ZTT5	*Pinus taeda* (Loblolly pine)	(E)-caffeoyl-CoA + S-adenosyl-L-methionine = (E)-feruloyl-CoA + H(+) + S-adenosyl-L-homocysteine	Aromatic compound metabolism; Phenylpropanoid biosynthesis.		lignin biosynthetic process [GO:0009809]; methylation [GO:0032259]	caffeoyl-CoA O-methyltransferase activity [GO:0042409]; metal ion binding [GO:0046872]	Methylates caffeoyl-CoA to feruloyl-CoA and 5-hydroxyferuloyl-CoA to sinapoyl-CoA. Plays a role in the synthesis of feruloylated polysaccharides. Involved in the reinforcement of the plant cell wall. Also involved in the responding to wounding or pathogen challenge by the increased formation of cell wall-bound ferulic acid polymers.
Outgroup IV + V	CCoAMT	Q9C5D7	*Arabidopsis thaliana* (Mouse-ear cress)	(E)-caffeoyl-CoA + S-adenosyl-L-methionine = (E)-feruloyl-CoA + H(+) + S-adenosyl-L-homocysteine	Aromatic compound metabolism; Phenylpropanoid biosynthesis.		lignin biosynthetic process [GO:0009809]; methylation [GO:0032259]	caffeoyl-CoA O-methyltransferase activity [GO:0042409]; metal ion binding [GO:0046872]; S-adenosylmethionine-dependent methyltransferase activity [GO:0008757]	Methylates caffeoyl-CoA to feruloyl-CoA and 5-hydroxyferuloyl-CoA to sinapoyl-CoA. Plays a role in the synthesis of feruloylated polysaccharides. Involved in the reinforcement of the plant cell wall. Also involved in the responding to wounding or pathogen challenge by the increased formation of cell wall-bound ferulic acid polymers (By similarity).
-	CCoAMT	Q7F8T6	*Oryza sativa* subsp. *japonica* (Rice)	2 S-adenosyl-L-methionine + tricetin = 3',5'-di-O-methyltricetin + 2 H(+) + 2 S-adenosyl-L-homocysteine		Expressed in stems only.	methylation [GO:0032259]	metal ion binding [GO:0046872]; O-methyltransferase activity [GO:0008171]; S-adenosylmethionine-dependent methyltransferase activity [GO:0008757]	Catalyzes the stepwise methylation of tricetin to its 3'-mono- and 3',5'-dimethyl ethers. No 3',4',5'-trimethylated ester derivatives are produced. Can use caffeoyl CoA, 5-hydroxyferulic acid, luteolin, tricetin, quercetin, myrcetin and 7,8-dihydroxyflavone as substrates, but not naringenin, apigenin or kaempferol. The 2,3-double bond and the O-dihydroxyl group of the substrate are both required for catalytic activity of the enzyme..
-	CCoAMT	Q9XGP7	*Oryza sativa* subsp. *japonica* (Rice)	2 S-adenosyl-L-methionine + tricetin = 3',5'-di-O-methyltricetin		Ubiquitous. Highest expression in	methylation [GO:0032259]	metal ion binding [GO:0046872]; O-methyltransferase	Catalyzes the stepwise methylation of tricetin to its 3'-mono- and 3',5'-dimethyl ethers. No 3',4',5'-

Group	Protein families	Entry	Organism	Catalytic activity	Pathway	Tissue specificity	Gene Ontology (biological process)	Gene Ontology (molecular function)	Function
				+ 2 H(+) + 2 S-adenosyl-L-homocysteine		stems and roots.		activity [GO:0008171]; S-adenosylmethionine-dependent methyltransferase activity [GO:0008757]	trimethylated ester derivatives are produced. Can use caffeoyl-CoA, 5-hydroxyferulic acid, luteolin, tricetin, quercetin, myrcetin and 7,8-dihydroxyflavone as substrates, but not naringenin, apigenin or kaempferol. The 2,3-double bond and the O-dihydroxyl group of the substrate are both required for catalytic activity of the enzyme.
I	COMT	A0A0N9HMN6	*Sinopodophyllum hexandrum* (Himalayan may apple) (*Podophyllum hexandrum*)	(-)-pluviatolide + S-adenosyl-L-methionine = (-)-bursehernin + H(+) + S-adenosyl-L-homocysteine	Aromatic compound metabolism; Phenylpropanoid biosynthesis.	Mostly expressed in stems, and, to a lower extent, in leaves.	aromatic compound biosynthetic process [GO:0019438]; methylation [GO:0032259]; phenylpropanoid biosynthetic process [GO:0009699]; response to wounding [GO:0009611]	O-methyltransferase activity [GO:0008171]; protein dimerization activity [GO:0046983]; S-adenosylmethionine-dependent methyltransferase activity [GO:0008757]	O-methyltransferase involved in the biosynthesis of etoposide, a chemotherapeutic compound of the topoisomerase inhibitor family. Catalyzes the methylation of (-)-pluviatolide to produce (-)-bursehernin.
I	COMT	C6TAY1	*Glycine max* (Soybean) (*Glycine hispida*)	a 4'-hydroxyflavanone + S-adenosyl-L-methionine = a 4'-methoxyflavanone + H(+) + S-adenosyl-L-homocysteine			aromatic compound biosynthetic process [GO:0019438]; methylation [GO:0032259]	flavanone 4'-O-methyltransferase activity [GO:0102767]; O-methyltransferase activity [GO:0008171]; protein dimerization activity [GO:0046983]; S-adenosylmethionine-dependent methyltransferase activity [GO:0008757]	S-adenosyl-L-methionine-dependent methyltransferase that catalyzes the 4'-methylation of naringenin (4',5,7-trihydroxyflavanone) into ponciretin (4'-methoxy-5,7-dihydroxyflavanone). In vitro, also able to convert apigenin, daidzein, genistein and quercetin into the 4'-O-methylated compounds acacetin, formononetin, biochanine A and 4'-methylated quercetin, respectively.
I	COMT	O22308	*Medicago sativa* (Alfalfa)	a 7-hydroxyisoflavone + S-adenosyl-L-methionine = a 7-methoxyisoflavone + H(+) + S-adenosyl-L-homocysteine	Phytoalexin biosynthesis; Medicarpin biosynthesis.		methylation [GO:0032259]	isoflavone 7-O-methyltransferase activity [GO:0033800]; protein dimerization activity [GO:0046983]	Transfers a methyl group to 7-hydroxyls of the isoflavones daidzein, genistein and 6,7,4'-trihydroxyisoflavone. Can also methylate (+)6a-hydroxymaackiain with lower efficiency.
I	COMT	O22309	*Medicago sativa* (Alfalfa)	a 7-hydroxyisoflavone + S-adenosyl-L-methionine = a 7-methoxyisoflavone + H(+) + S-adenosyl-L-homocysteine	Phytoalexin biosynthesis; Medicarpin biosynthesis.		methylation [GO:0032259]	isoflavone 7-O-methyltransferase activity [GO:0033800]; protein dimerization activity [GO:0046983]	Transfers a methyl group to 7-hydroxyls of the isoflavones daidzein, genistein and 6,7,4'-trihydroxyisoflavone. Can also methylate (+)6a-hydroxymaackiain with lower efficiency.
I	COMT	O24305	*Pisum sativum* (Garden pea)	(+)-6a-hydroxymaackiain + S-adenosyl-L-methionine = (+)-pisatin + H(+) + S-adenosyl-L-homocysteine			methylation [GO:0032259]	6a-hydroxymaackiain-3-O-methyltransferase activity [GO:0102671]; isoflavone 4'-O-methyltransferase	Methyltransferase involved in the phytoalexin pisatin biosynthesis. Has both 3- and 4'-O-methyltransferase activities. Can use (+)-6a-hydroxymaackiain, 2,7,4'-trihydroxyisoflavanone and with much

Group	Protein families	Entry	Organism	Catalytic activity	Pathway	Tissue specificity	Gene Ontology (biological process)	Gene Ontology (molecular function)	Function
								activity [GO:0030746]; O-methyltransferase activity [GO:0008171]; protein dimerization activity [GO:0046983]	less activity (+)-medicarpin as substrates, but not (-)-6a-hydroxymaackiain, daidzein, formononetin or isoliquiritigenin. May be involved in formononetin biosynthesis.
I	COMT	O24529	*Medicago sativa* (Alfalfa)	a 7-hydroxyisoflavone + S-adenosyl-L-methionine = a 7-methoxyisoflavone + H(+) + S-adenosyl-L-homocysteine	Phytoalexin biosynthesis; Medicarpin biosynthesis.		methylation [GO:0032259]	isoflavone 7-O-methyltransferase activity [GO:0033800]; protein dimerization activity [GO:0046983]	Transfers a methyl group to 7-hydroxyls of the isoflavones daidzein, genistein and 6,7,4'-trihydroxyisoflavone. Can also methylate (+)6a-hydroxymaackiain with lower efficiency.
I	COMT	P0DH60	*Pisum sativum* (Garden pea)	(+)-6a-hydroxymaackiain + S-adenosyl-L-methionine = (+)-pisatin + H(+) + S-adenosyl-L-homocysteine			methylation [GO:0032259]	6a-hydroxymaackiain-3-O-methyltransferase activity [GO:0102671]; O-methyltransferase activity [GO:0008171]; protein dimerization activity [GO:0046983]; S-adenosylmethionine-dependent methyltransferase activity [GO:0008757]	3-O-methyltransferase involved in the phytoalexin pisatin biosynthesis. Can use (+)-6a-hydroxymaackiain, (+)-maackiain and with a lower activity (+)-medicarpin and 2,7,4'-trihydroxyisoflavanone as substrates, but not (-)-6a-hydroxymaackiain, daidzein, formononetin or isoliquiritigenin.
I	COMT	Q29U70	*Medicago truncatula* (Barrel medic) (*Medicago tribuloides*)	a 4'-hydroxyisoflavone + S-adenosyl-L-methionine = a 4'-methoxyisoflavone + H(+) + S-adenosyl-L-homocysteine			methylation [GO:0032259]	2,7,4'-trihydroxyisoflavanone-4'-O-methyltransferase activity [GO:0102670]; isoflavone 4'-O-methyltransferase activity [GO:0030746]; O-methyltransferase activity [GO:0008171]; protein dimerization activity [GO:0046983]	2-hydroxyisoflavanone 4'-O-methyltransferase involved in the biosynthesis of the phytoalexin medicarpin. Has also an in vitro (+)-6a-hydroxymaackiain-3-0-methyltransferase activity, converting the pterocarpan 6a-hydroxymaackiain into pisatin. No activity with di- or trihydroxylated isoflavones, including daidzein and genistein, or with (-)-medicarpin and maackiain. The dual activity for either 3- or 4'-O-methylation depends upon substrate availability.
I	COMT	Q84KK4	*Lotus japonicus* (*Lotus corniculatus* var. *japonicus*)	a 4'-hydroxyisoflavone + S-adenosyl-L-methionine = a 4'-methoxyisoflavone + H(+) + S-adenosyl-L-homocysteine			isoflavonoid phytoalexin biosynthetic process [GO:0009701]; methylation [GO:0032259]	2,7,4'-trihydroxyisoflavanone-4'-O-methyltransferase activity [GO:0102670]; isoflavone 4'-O-methyltransferase activity	2-hydroxyisoflavanone 4'-O-methyltransferase involved in the biosynthesis of formononetin. Can use 2,7,4'-trihydroxyisoflavanone as substrate, but not daidzein.

Group	Protein families	Entry	Organism	Catalytic activity	Pathway	Tissue specificity	Gene Ontology (biological process)	Gene Ontology (molecular function)	Function
								[GO:0030746]; O-methyltransferase activity [GO:0008171]; protein dimerization activity [GO:0046983]	
I	COMT	Q84KK5	*Glycyrrhiza echinata* (Licorice)	a 7-hydroxyisoflavone + S-adenosyl-L-methionine = a 7-methoxyisoflavone + H(+) + S-adenosyl-L-homocysteine			isoflavonoid biosynthetic process [GO:0009717]; methylation [GO:0032259]	isoflavone 7-O-methyltransferase activity [GO:0033800]; protein dimerization activity [GO:0046983]	7-O-methyltransferase involved in the biosynthesis of isoformononetin. Can use daidzein as substrate, but not medicarpin or 2,7,4'-trihydroxyisoflavanone.
I	COMT	Q84KK6	*Glycyrrhiza echinata* (Licorice)	a 4'-hydroxyisoflavone + S-adenosyl-L-methionine = a 4'-methoxyisoflavone + H(+) + S-adenosyl-L-homocysteine			isoflavonoid phytoalexin biosynthetic process [GO:0009701]; methylation [GO:0032259]	2,7,4'-trihydroxyisoflavanone-4'-O-methyltransferase activity [GO:0102670]; isoflavone 4'-O-methyltransferase activity [GO:0030746]; O-methyltransferase activity [GO:0008171]; protein dimerization activity [GO:0046983]	2-hydroxyisoflavanone 4'-O-methyltransferase involved in the biosynthesis of formononetin. Can use 2,7,4'-trihydroxyisoflavanone, (+)-6a-hydroxymaackiain or medicarpin as substrate, but not daidzein or (-)-6a-hydroxymaackiain.
II	COMT	B0EXJ8	*Catharanthus roseus* (Madagascar periwinkle) (*Vinca rosea*)	16-hydroxytabersonine + S-adenosyl-L-methionine = 16-methoxytabersonine + H(+) + S-adenosyl-L-homocysteine	Alkaloid biosynthesis; Vindoline biosynthesis.	Expressed in leaves and flowers. Detected in stems and roots. In leaves, expressed in epidermal cells.	alkaloid biosynthetic process [GO:0009821]; methylation [GO:0032259]	11-O-demethyl-17-O-deacetylvindoline O-methyltransferase activity [GO:0030766]; O-methyltransferase activity [GO:0008171]; protein homodimerization activity [GO:0042803]	16-O-methyltransferase involved in the biosynthesis of vindoline. Highly specific for 16-hydroxytabersonine. No activity with tabersonine, 3-hydroxytyramine, 4-hydroxytyramine, 5-hydroxytryptamine (5HT), 2,3-dihydro-3-hydroxytabersonine, lochnericine, hoerhammericine, 16-hydroxy-2,3-dihydro-3-hydroxytabersonine, 16-hydroxylochnericine, 16-hydroxyhoerhammericine, quercetin, kaempferol and caffeic acid as substrates.
II	COMT	Q8GSN1	*Catharanthus roseus* (Madagascar periwinkle) (*Vinca rosea*)	S-adenosyl-L-methionine + a 3'-hydroxyflavonoid = S-adenosyl-L-homocysteine + a 3'-methoxyflavonoid			methylation [GO:0032259]	laricitrin 5'-O-methyltransferase activity [GO:0070448]; myricetin 3'-O-methyltransferase activity [GO:0033799]; protein dimerization activity [GO:0046983]	Methylates myricetin and dihydromyricetin at 2 sites. Inactive towards 16-hydroxytabersonine, the phenylpropanoids 5-hydroxyferulate, caffeate and their CoA-esters, flavones and flavanones possessing 2 or 3 B-ring hydroxyl groups.

Group	Protein families	Entry	Organism	Catalytic activity	Pathway	Tissue specificity	Gene Ontology (biological process)	Gene Ontology (molecular function)	Function
III	COMT	B6VJS4	*Vitis vinifera* (Grape)	2 S-adenosyl-L-methionine + trans-resveratrol = 2 H(+) + pterostilbene + 2 S-adenosyl-L-homocysteine			aromatic compound biosynthetic process [GO:0019438]; methylation [GO:0032259]	O-methyltransferase activity [GO:0008171]; protein dimerization activity [GO:0046983]; resveratrol 3,5-O-dimethyltransferase activity [GO:0102303]; S-adenosylmethionine-dependent methyltransferase activity [GO:0008757]	Catalyzes the biosynthesis of pterostilbene from resveratrol. Pterostilbene has both antifungal and pharmacological properties. Also has activity toward resveratrol monomethyl ether (RME).
III	COMT	Q6VMW0	*Mentha piperita* (Peppermint) (*Mentha aquatica* x *Mentha spicata*)	3,3',4',5,7,8-hexahydroxyflavone + S-adenosyl-L-methionine = 3,3',4',5,7-pentahydroxy-8-methoxyflavone + H(+) + S-adenosyl-L-homocysteine	Flavonoid metabolism.		flavonoid metabolic process [GO:0009812]; methylation [GO:0032259]	8-hydroxyquercitin 8-O-methyltransferase activity [GO:0030761]; O-methyltransferase activity [GO:0008171]; protein dimerization activity [GO:0046983]	Flavonoid 8-O-methyltransferase involved in the biosynthesis of polymethoxylated flavonoids natural products such as pebrellin, aroma compounds which contribute to the flavor of peppermint, and exhibit pharmacological activities such as anti-allergic, anti-oxidant, antibacterial, anti-proliferative, and anti-inflammatory effects. Catalyzes S-adenosylmethionine-dependent regioselective 8-O-methylation of flavonoids; active on various hydroxylated flavonoid substrates, including 7,8,3'4'-tetrahydroxy-flavone, 7,8,4'-trihydroxy-flavone and 8-hydroxy-flavone 7-methyl ether.
III	COMT	Q93WU2	*Ocimum basilicum* (Sweet basil)	S-adenosyl-L-methionine + trans-isoeugenol = H(+) + S-adenosyl-L-homocysteine + trans-isomethyleugenol	Aromatic compound metabolism; Phenylpropanoid biosynthesis.	Specifically expressed in the peltate glandular trichomes on the surface of the young basil leaves.	methylation [GO:0032259]; phenylpropanoid biosynthetic process [GO:0009699]	(iso)eugenol O-methyltransferase activity [GO:0050630]; protein dimerization activity [GO:0046983]; S-adenosyl-L-methionine:eugenol-O-methyltransferase activity [GO:0102719]	Phenylpropene O-methyltransferase that catalyzes the methylation of the para-4-hydroxyl of eugenol to methyleugenol. Can also convert chavicol to methylchavicol but with less affinity.
III	COMT	Q93WU3	*Ocimum basilicum* (Sweet basil)	S-adenosyl-L-methionine + trans-isoeugenol = H(+) + S-adenosyl-L-homocysteine + trans-isomethyleugenol	Aromatic compound metabolism; Phenylpropanoid biosynthesis.	Specifically expressed in the peltate glandular trichomes on the surface of the young basil leaves.	methylation [GO:0032259]; phenylpropanoid biosynthetic process [GO:0009699]	(iso)eugenol O-methyltransferase activity [GO:0050630]; protein dimerization activity [GO:0046983]; S-adenosyl-L-methionine:eugenol-	Phenylpropene O-methyltransferase that catalyzes the methylation of the para-4-hydroxyl of chavicol to methylchavicol. Can also convert eugenol to methyleugenol but with less affinity.

Group	Protein families	Entry	Organism	Catalytic activity	Pathway	Tissue specificity	Gene Ontology (biological process)	Gene Ontology (molecular function)	Function
V	COMT	A0A0N9HTA1	*Sinopodophyllum hexandrum* (Himalayan may apple) (*Podophyllum hexandrum*)	(-)-5'-demethylyatein + S-adenosyl-L-methionine = (-)-yatein + H(+) + S-adenosyl-L-homocysteine	Aromatic compound metabolism; Phenylpropanoid biosynthesis.	Mostly expressed in stems, and, to a lower extent, in leaves.	aromatic compound biosynthetic process [GO:0019438]; methylation [GO:0032259]; phenylpropanoid biosynthetic process [GO:0009699]; response to wounding [GO:0009611]	O-methyltransferase activity [GO:0102719] O-methyltransferase activity [GO:0008171]; protein dimerization activity [GO:0046983]; S-adenosylmethionine-dependent methyltransferase activity [GO:0008757]	O-methyltransferase involved in the biosynthesis of etoposide, a chemotherapeutic compound of the topoisomerase inhibitor family. Catalyzes the methylation of (-)-5'-demethylyatein to produce (-)-yatein.
V	COMT	A0A166U5H3	*Kitagawia praeruptora* (*Peucedanum praeruptorum*)	bergaptol + S-adenosyl-L-methionine = bergapten + S-adenosyl-L-homocysteine	Aromatic compound metabolism; Secondary metabolite biosynthesis.	Mostly expressed in roots and, to a lower extent, in stems and leaves.	aromatic compound biosynthetic process [GO:0019438]; coumarin biosynthetic process [GO:0009805]; methylation [GO:0032259]; response to jasmonic acid [GO:0009753]	5-hydroxyfuranocoumarin 5-O-methyltransferase activity [GO:0030752]; O-methyltransferase activity [GO:0008171]; protein dimerization activity [GO:0046983]; S-adenosylmethionine-dependent methyltransferase activity [GO:0008757]	O-methyltransferase involved in the biosynthesis of furocoumarins natural products such as bergapten, a photosensitizer used for medical purpose such as treating psoriasis and vitiligo or facilitating resistance to microbial infection and other stresses. Catalyzes specifically the methylation of bergaptol. Not active on xanthotol, isoscopoletin, scopoletin and esculetin.
V	COMT	A0A3Q7I9E2	*Solanum lycopersicum* (Tomato) (*Lycopersicon esculentum*)	catechol + S-adenosyl-L-methionine = guaiacol + H(+) + S-adenosyl-L-homocysteine			aromatic compound biosynthetic process [GO:0019438]; methylation [GO:0032259]	O-methyltransferase activity [GO:0008171]; protein dimerization activity [GO:0046983]; S-adenosylmethionine-dependent methyltransferase activity [GO:0008757]	O-methyltransferase that catalyzes the conversion of catechol to guaiacol. Involved in the production of guaiacol in fruits.
V	COMT	A0A4P8DY91	*Kitagawia praeruptora* (*Peucedanum praeruptorum*)	bergaptol + S-adenosyl-L-methionine = bergapten + S-adenosyl-L-homocysteine	Aromatic compound metabolism; Secondary metabolite biosynthesis.	Expressed ubiquitously.	aromatic compound biosynthetic process [GO:0019438]; coumarin biosynthetic process [GO:0009805]; methylation [GO:0032259]; response to hydrogen peroxide [GO:0042542]; response to UV [GO:0009411]	catechol O-methyltransferase activity [GO:0016206]; L-dopa O-methyltransferase activity [GO:0102084]; O-methyltransferase activity [GO:0008171]; orcinol O-methyltransferase	O-methyltransferase involved in the biosynthesis of methoxylated coumarins natural products such as isoscopoletin, scopoletin, xanthotoxin and bergapten, photosensitizers used for medical purpose such as treating psoriasis and vitiligo or facilitating resistance to microbial infection and other stresses. Catalyzes the methylation of esculetin, bergaptol and xanthotoxol, but seems inactive on scopoletin and isoscopoletin.

Group	Protein families	Entry	Organism	Catalytic activity	Pathway	Tissue specificity	Gene Ontology (biological process)	Gene Ontology (molecular function)	Function
								activity [GO:0102938]; protein dimerization activity [GO:0046983]; S-adenosylmethionine-dependent methyltransferase activity [GO:0008757]	
V	COMT	A8J6X1	*Glehnia littoralis* (Beach silvertop) (*Phellopterus littoralis*)	a 5-hydroxyfurocoumarin + S-adenosyl-L-methionine = a 5-methoxyfurocoumarin + H(+) + S-adenosyl-L-homocysteine			methylation [GO:0032259]	5-hydroxyfuranocoumarin 5-O-methyltransferase activity [GO:0030752]; O-methyltransferase activity [GO:0008171]; protein dimerization activity [GO:0046983]	
V	COMT	A8QW52	*Sorghum bicolor* (Sorghum) (*Sorghum vulgare*)	S-adenosyl-L-methionine + trans-isoeugenol = H(+) + S-adenosyl-L-homocysteine + trans-isomethyleugenol		Expressed predominantly in root hairs.	aromatic compound biosynthetic process [GO:0019438]; methylation [GO:0032259]	(iso)eugenol O-methyltransferase activity [GO:0050630]; O-methyltransferase activity [GO:0008171]; protein dimerization activity [GO:0046983]; S-adenosyl-L-methionine:eugenol-O-methyltransferase activity [GO:0102719]; S-adenosylmethionine-dependent methyltransferase activity [GO:0008757]	O-methyltransferase. Substrate preference is eugenol >> orcinol monomethyl ether > resorcinol monomethyl ether.
V	COMT	A9X7L0	*Ruta graveolens* (Common rue)	anthranilate + S-adenosyl-L-methionine = H(+) + N-methylanthranilate + S-adenosyl-L-homocysteine		Expressed in leaves, flowers, stems and roots. Detected in the vascular tissues in stems, in the rhizodermis or the endodermis of roots, in the	methylation [GO:0032259]	anthranilate N-methyltransferase activity [GO:0030774]; O-methyltransferase activity [GO:0008171]; protein dimerization activity [GO:0046983]	Involved in the biosynthesis of acridine alkaloids. N-methyltransferase with a strict substrate specificity for anthranilate. No activity with anthranilic acid methyl ester, anthraniloyl CoA, 3- or 4-amino-benzoic acid, salicylic acid, catechol, eugenol, caffeic acid, quercetin, theobromin, theophyllin, putrescine and nicotinic acid among others.

Group	Protein families	Entry	Organism	Catalytic activity	Pathway	Tissue specificity	Gene Ontology (biological process)	Gene Ontology (molecular function)	Function
						inside of carpels, in the central vascular bundles of the syncarp ovary and in the secretory oil glands located around the outer ovary wall.			
V	COMT	B8RCD3	*Pimpinella anisum* (Anise) (*Anisum vulgare*)	S-adenosyl-L-methionine + trans-anol = H(+) + S-adenosyl-L-homocysteine + trans-anethole	Aromatic compound metabolism; Phenylpropanoid biosynthesis.	Highly expressed in developing fruits. Expressed at low levels in roots, young leaves, buds and flowers.	methylation [GO:0032259]; phenylpropanoid biosynthetic process [GO:0009699]	(iso)eugenol O-methyltransferase activity [GO:0050630]; protein dimerization activity [GO:0046983]	Phenylpropene O-methyltransferase that catalyzes the conversion of trans-anol to trans-anethole and isoeugenol to isomethyleugenol. Phenylpropenes are the primary constituents of various essential plant oils. They are produced as antimicrobial and antianimal compounds, or as floral attractants of pollinators.
V	COMT	I3V6A7	*Papaver somniferum* (Opium poppy)	(S)-scoulerine + S-adenosyl-L-methionine = (S)-tetrahydrocolumbamine + H(+) + S-adenosyl-L-homocysteine		Highly expressed in capsules. Expressed is stems. Expressed at low levels in roots.	benzyl isoquinoline alkaloid biosynthetic process [GO:0009708]; methylation [GO:0032259]	(S)-scoulerine 9-O-methyltransferase activity [GO:0030777]; methyltransferase activity [GO:0008168]; O-methyltransferase activity [GO:0008171]; protein dimerization activity [GO:0046983]; tetrahydrocolumbamin e 2-O-methyltransferase activity [GO:0030762]	Methyltransferase involved in the biosynthesis of the benzylisoquinoline alkaloid noscapine. Catalyzes the conversion of (S)-scoulerine to (S)-tetrahydrocolumbamine. Can convert (S)-tetrahydrocolumbamine to tetrahydropalmatine. Can convert (S)-norreticuline to (S)-norcodamine (PubMed:22535422). Can convert (S)-reticuline to (S)-codamine. Substrate preference is (S)-scoulerine > (S)-tetrahydrocolumbamine > (S)-norreticuline > (S)-reticuline.
V	COMT	O04385	*Clarkia breweri* (Fairy fans) (*Eucharidium breweri*)	S-adenosyl-L-methionine + trans-isoeugenol = H(+) + S-adenosyl-L-homocysteine + trans-isomethyleugenol		Expressed in petals, style and stamens, but not in stigma, sepals, leaves or stem tissues.	methylation [GO:0032259]	(iso)eugenol O-methyltransferase activity [GO:0050630]; protein dimerization activity [GO:0046983]; S-adenosyl-L-methionine:eugenol-O-methyltransferase activity [GO:0102719]	Catalyzes the methylation of the para-4-hydroxyl of both eugenol and (iso)eugenol to methyleugenol and isomethyleugenol, respectively. The resulting products are part of a complex mixture of low-molecular-weight volatile compounds emitted by the flowers to attract pollinators.
V	COMT	O23760	*Clarkia breweri* (Fairy fans)	(E)-caffeate + S-adenosyl-L-methionine	Aromatic compound		lignin biosynthetic process	caffeate O-methyltransferase	Catalyzes the conversion of caffeic acid to ferulic acid and of 5-hydroxyferulic

Group	Protein families	Entry	Organism	Catalytic activity	Pathway	Tissue specificity	Gene Ontology (biological process)	Gene Ontology (molecular function)	Function
			(*Eucharidium breweri*)	= (E)-ferulate + H(+) + S-adenosyl-L-homocysteine	metabolism; Phenylpropanoid biosynthesis.		[GO:0009809]; methylation [GO:0032259]	activity [GO:0047763]; protein dimerization activity [GO:0046983]	acid to sinapic acid. The resulting products may subsequently be converted to the corresponding alcohols that are incorporated into lignins.
V	COMT	O81646	*Capsicum chinense* (Scotch bonnet) (Bonnet pepper)	(E)-caffeate + S-adenosyl-L-methionine = (E)-ferulate + H(+) + S-adenosyl-L-homocysteine	Aromatic compound metabolism; Phenylpropanoid biosynthesis.		lignin biosynthetic process [GO:0009809]; methylation [GO:0032259]	caffeate O-methyltransferase activity [GO:0047763]; protein dimerization activity [GO:0046983]	Catalyzes the conversion of caffeic acid to ferulic acid and of 5-hydroxyferulic acid to sinapic acid. The resulting products may subsequently be converted to the corresponding alcohols that are incorporated into lignins.
V	COMT	O82054	*Saccharum officinarum* (Sugarcane)	(E)-caffeate + S-adenosyl-L-methionine = (E)-ferulate + H(+) + S-adenosyl-L-homocysteine	Aromatic compound metabolism; Phenylpropanoid biosynthesis.		lignin biosynthetic process [GO:0009809]; methylation [GO:0032259]	caffeate O-methyltransferase activity [GO:0047763]; protein dimerization activity [GO:0046983]	Catalyzes the conversion of caffeic acid to ferulic acid and of 5-hydroxyferulic acid to sinapic acid. The resulting products may subsequently be converted to the corresponding alcohols that are incorporated into lignins.
V	COMT	P28002	*Medicago sativa* (Alfalfa)	(E)-caffeate + S-adenosyl-L-methionine = (E)-ferulate + H(+) + S-adenosyl-L-homocysteine	Aromatic compound metabolism; Phenylpropanoid biosynthesis.	More abundant in roots and stems.	lignin biosynthetic process [GO:0009809]; methylation [GO:0032259]; phenylpropanoid biosynthetic process [GO:0009699]	caffeate O-methyltransferase activity [GO:0047763]; protein dimerization activity [GO:0046983]; S-adenosylmethionine-dependent methyltransferase activity [GO:0008757]	Catalyzes the conversion of caffeic acid to ferulic acid and of 5-hydroxyferulic acid to sinapic acid (Probable). The resulting products may subsequently be converted to the corresponding alcohols that are incorporated into lignins (Probable).
V	COMT	P46484	*Eucalyptus gunnii* (Cider gum)	(E)-caffeate + S-adenosyl-L-methionine = (E)-ferulate + H(+) + S-adenosyl-L-homocysteine	Aromatic compound metabolism; Phenylpropanoid biosynthesis.		lignin biosynthetic process [GO:0009809]; methylation [GO:0032259]	caffeate O-methyltransferase activity [GO:0047763]; protein dimerization activity [GO:0046983]	Catalyzes the conversion of caffeic acid to ferulic acid and of 5-hydroxyferulic acid to sinapic acid. The resulting products may subsequently be converted to the corresponding alcohols that are incorporated into lignins.
V	COMT	P59049	*Chrysosplenium americanum* (American golden saxifrage)	(E)-5-hydroxyferulate + S-adenosyl-L-methionine = (E)-sinapate + H(+) + S-adenosyl-L-homocysteine	Flavonoid metabolism.		flavonoid metabolic process [GO:0009812]; methylation [GO:0032259]	caffeate O-methyltransferase activity [GO:0047763]; luteolin O-methyltransferase activity [GO:0030744]; protein dimerization activity [GO:0046983]; quercetin 3'-O-methyltransferase activity [GO:0102822]; quercetin 3-O-methyltransferase activity [GO:0030755]	Catalyzes the 3'-O-methylation of the flavonoids luteolin and quercetin. Catalyzes the 3- of 5-O-methylation of the phenylpropanoids caffeate and 5-hydroxyferulate. Substrate preference is 5-hydroxyferulate > luteolin > quercetin > caffeate. Apigenin, kempferol and 3,4-dimethylquercetin do not seem to be substrates for methylation.

Group	Protein families	Entry	Organism	Catalytic activity	Pathway	Tissue specificity	Gene Ontology (biological process)	Gene Ontology (molecular function)	Function
V	COMT	P93324	*Medicago sativa* (Alfalfa)	isoliquiritigenin + S-adenosyl-L-methionine = 2'-O-methylisoliquiritigenin + S-adenosyl-L-homocysteine		Roots (at protein level). Expressed mainly in roots, and to a lesser extent in root nodules. In the roots, expression is not detected in the root tip or the cells immediately behind the tip, but is detected in tissues starting 1.5-2.0 mm distal to the root tip. Detected in the epidermal and cortical cells of 2 day old roots, with lower levels in vascular tissue.	methylation [GO:0032259]	isoliquiritigenin 2'-O-methyltransferase activity [GO:0033802]; licodione 2'-O-methyltransferase activity [GO:0030751]; protein dimerization activity [GO:0046983]	Methylates the 2'-hydroxyl of isoliquiritigenin and licodione. Does not methylate narigenin chalcone, caffeic acid or daidzein. Involved in the root nodulation initiation by promoting the biosynthesis of nod-inducing molecules.
V	COMT	Q00763	*Populus tremuloides* (Quaking aspen)	(E)-caffeate + S-adenosyl-L-methionine = (E)-ferulate + H(+) + S-adenosyl-L-homocysteine	Aromatic compound metabolism; Phenylpropanoid biosynthesis.	Xylem.	lignin biosynthetic process [GO:0009809]; methylation [GO:0032259]	caffeate O-methyltransferase activity [GO:0047763]; protein dimerization activity [GO:0046983]	Catalyzes the conversion of caffeic acid to ferulic acid and of 5-hydroxyferulic acid to sinapic acid. The resulting products may subsequently be converted to the corresponding alcohols that are incorporated into lignins.
V	COMT	Q06509	*Zea mays* (Maize)	(E)-caffeate + S-adenosyl-L-methionine = (E)-ferulate + H(+) + S-adenosyl-L-homocysteine	Aromatic compound metabolism; Phenylpropanoid biosynthesis.	Confined to the vascular tissues of organs undergoing lignification such as stems and roots.	aromatic compound biosynthetic process [GO:0019438]; flavonol biosynthetic process [GO:0051555]; lignin biosynthetic process [GO:0009809]; melatonin biosynthetic process [GO:0030187]; methylation [GO:0032259]; regulation of lignin biosynthetic process [GO:1901141];	acetylserotonin O-methyltransferase activity [GO:0017096]; caffeate O-methyltransferase activity [GO:0047763]; luteolin O-methyltransferase activity [GO:0030744]; O-methyltransferase activity [GO:0008171]; protein dimerization activity	Catalyzes the conversion of caffeic acid to ferulic acid and of 5-hydroxyferulic acid to sinapic acid (By similarity). The resulting products may subsequently be converted to the corresponding alcohols that are incorporated into lignins. Can use the flavone tricetin (5,7,3',4',5'-pentahydroxyflavone) as the preferred substrate and give rise to its 3',5'-dimethyl derivative, tricin (3',5'-dimethoxy-5,7,4'-trihydroxyflavone), as the major product, and selgin to a lower extent. Tricin exhibits potential benefits for human health including relaxant effect on smooth muscle of intestinal tissues, antioxidant effect, antihistaminic activity, and growth

Group	Protein families	Entry	Organism	Catalytic activity	Pathway	Tissue specificity	Gene Ontology (biological process)	Gene Ontology (molecular function)	Function
							response to wounding [GO:0009611]	[GO:0046983]; S-adenosylmethionine-dependent methyltransferase activity [GO:0008757]; tricetin O-methytransferase activity [GO:0102146]	inhibition of human malignant breast tumor cells and colon cancer cells. Can also use luteolin, quercetin and 5-hydroxyferulic acid (5HF) as substrates.
V	COMT	Q0IP69	*Oryza sativa* subsp. *japonica* (Rice)	(2S)-naringenin + S-adenosyl-L-methionine = (2S)-sakuranetin + H(+) + S-adenosyl-L-homocysteine			aromatic compound biosynthetic process [GO:0019438]; methylation [GO:0032259]	methyltransferase activity [GO:0008168]; naringenin 7-O-methyltransferase activity [GO:0102766]; O-methyltransferase activity [GO:0008171]; protein dimerization activity [GO:0046983]; S-adenosylmethionine-dependent methyltransferase activity [GO:0008757]	S-adenosyl-L-methionine-dependent methyltransferase involved in the biosynthesis of the sakuranetin, an inducible defense mechanism of O.sativa against pathogen attack.
V	COMT	Q38J50	*Triticum aestivum* (Wheat)	3 S-adenosyl-L-methionine + tricetin = 3',4',5'-O-trimethyltricetin + 3 H(+) + 3 S-adenosyl-L-homocysteine		Expressed in roots, stems and leaves.	aromatic compound biosynthetic process [GO:0019438]; flavonoid biosynthetic process [GO:0009813]; lignin biosynthetic process [GO:0009809]; methylation [GO:0032259]	O-methyltransferase activity [GO:0008171]; protein dimerization activity [GO:0046983]; S-adenosylmethionine-dependent methyltransferase activity [GO:0008757]	Flavonoid B-ring-specific O-methyltransferase with a preference for flavones > dihydroflavones > flavonols that possess at least two B-ring hydroxyl groups. Active with tricetin, 5-hydroxyferulic acid, luteolin, quercitin, eriodictyol, quercetagetin, taxifolin, gossypetin and myricetin. No activity with naringenin, apigenin, kaempferol, 7,8-dihydroxy- or 5,7,8-trihydroxy flavones, chlorogenic acid, gallic acid or daphnetin. Catalyzes the sequential O-methylation of tricetin via 3'-O-methyltricetin, 3',5'-O-methyltricetin to 3',4',5'-O-trimethyltricetin. May also be involved in S lignin biosynthesis.
V	COMT	Q39522	*Coptis japonica* (Japanese goldthread)	(S)-scoulerine + S-adenosyl-L-methionine = (S)-tetrahydrocolumbamine + H(+) + S-adenosyl-L-homocysteine			methylation [GO:0032259]	(S)-scoulerine 9-O-methyltransferase activity [GO:0030777]; O-methyltransferase activity [GO:0008171]; protein dimerization activity [GO:0046983]	Produces a precursor of protoberberine alkaloids.

Group	Protein families	Entry	Organism	Catalytic activity	Pathway	Tissue specificity	Gene Ontology (biological process)	Gene Ontology (molecular function)	Function
V	COMT	Q41086	*Populus tremuloides* (Quaking aspen)	(E)-caffeate + S-adenosyl-L-methionine = (E)-ferulate + H(+) + S-adenosyl-L-homocysteine	Aromatic compound metabolism; Phenylpropanoid biosynthesis.		lignin biosynthetic process [GO:0009809]; methylation [GO:0032259]	caffeate O-methyltransferase activity [GO:0047763]; protein dimerization activity [GO:0046983]	Catalyzes the conversion of caffeic acid to ferulic acid and of 5-hydroxyferulic acid to sinapic acid. The resulting products may subsequently be converted to the corresponding alcohols that are incorporated into lignins.
V	COMT	Q42653	*Chrysosplenium americanum* (American golden saxifrage)	(E)-5-hydroxyferulate + S-adenosyl-L-methionine = (E)-sinapate + H(+) + S-adenosyl-L-homocysteine	Flavonoid metabolism.		flavonoid metabolic process [GO:0009812]; methylation [GO:0032259]	caffeate O-methyltransferase activity [GO:0047763]; luteolin O-methyltransferase activity [GO:0030744]; protein dimerization activity [GO:0046983]; quercetin 3'-O-methyltransferase activity [GO:0102822]; quercetin 3-O-methyltransferase activity [GO:0030755]	Catalyzes the 3'-O-methylation of the flavonoids luteolin and quercetin (PubMed:9514654). Catalyzes the 3- of 5-O-methylation of the phenylpropanoids caffeate and 5-hydroxyferulate (PubMed:9514654). Substrate preference is 5-hydroxyferulate > luteolin > quercetin > caffeate (PubMed:9514654). Apigenin, kempferol and 3,4-dimethylquercetin do not seem to be substrates for methylation (PubMed:9514654). {ECO:0000269|PubMed:9514654}.
V	COMT	Q43046	*Populus kitakamiensis* (Aspen) (*Populus sieboldii* x *Populus grandidentata*)	(E)-caffeate + S-adenosyl-L-methionine = (E)-ferulate + H(+) + S-adenosyl-L-homocysteine	Aromatic compound metabolism; Phenylpropanoid biosynthesis.		lignin biosynthetic process [GO:0009809]; methylation [GO:0032259]	caffeate O-methyltransferase activity [GO:0047763]; protein dimerization activity [GO:0046983]	Catalyzes the conversion of caffeic acid to ferulic acid and of 5-hydroxyferulic acid to sinapic acid. The resulting products may subsequently be converted to the corresponding alcohols that are incorporated into lignins.
V	COMT	Q43047	*Populus kitakamiensis* (Aspen) (*Populus sieboldii* x *Populus grandidentata*)	(E)-caffeate + S-adenosyl-L-methionine = (E)-ferulate + H(+) + S-adenosyl-L-homocysteine	Aromatic compound metabolism; Phenylpropanoid biosynthesis.		lignin biosynthetic process [GO:0009809]; methylation [GO:0032259]	caffeate O-methyltransferase activity [GO:0047763]; protein dimerization activity [GO:0046983]	Catalyzes the conversion of caffeic acid to ferulic acid and of 5-hydroxyferulic acid to sinapic acid. The resulting products may subsequently be converted to the corresponding alcohols that are incorporated into lignins.
V	COMT	Q43239	*Zinnia violacea* (Garden zinnia) (*Zinnia elegans*)	(E)-caffeate + S-adenosyl-L-methionine = (E)-ferulate + H(+) + S-adenosyl-L-homocysteine	Aromatic compound metabolism; Phenylpropanoid biosynthesis.		lignin biosynthetic process [GO:0009809]; methylation [GO:0032259]	caffeate O-methyltransferase activity [GO:0047763]; protein dimerization activity [GO:0046983]	Catalyzes the conversion of caffeic acid to ferulic acid and of 5-hydroxyferulic acid to sinapic acid. The resulting products may subsequently be converted to the corresponding alcohols that are incorporated into lignins.
V	COMT	Q43609	*Prunus dulcis* (Almond) (*Amygdalus dulcis*)	(E)-caffeate + S-adenosyl-L-methionine = (E)-ferulate + H(+) + S-adenosyl-L-homocysteine	Aromatic compound metabolism; Phenylpropanoid biosynthesis.		lignin biosynthetic process [GO:0009809]; methylation [GO:0032259]	caffeate O-methyltransferase activity [GO:0047763]; protein dimerization activity [GO:0046983]	Catalyzes the conversion of caffeic acid to ferulic acid and of 5-hydroxyferulic acid to sinapic acid. The resulting products may subsequently be converted to the corresponding alcohols that are incorporated into lignins.
V	COMT	Q6T1F6	*Ammi majus* (Bishop's weed)	a 5-hydroxyfurocoumarin + S-adenosyl-L-			methylation [GO:0032259]	5-hydroxyfuranocoumarin 5-O-	

Group	Protein families	Entry	Organism	Catalytic activity	Pathway	Tissue specificity	Gene Ontology (biological process)	Gene Ontology (molecular function)	Function
				methionine = a 5-methoxyfurocoumarin + H(+) + S-adenosyl-L-homocysteine				methyltransferase activity [GO:0030752]; O-methyltransferase activity [GO:0008171]; protein dimerization activity [GO:0046983]	
V	COMT	Q6ZD89	*Oryza sativa* subsp. *japonica* (Rice)	a 3'-hydroxyflavone + S-adenosyl-L-methionine = a 3'-methoxyflavone + H(+) + S-adenosyl-L-homocysteine	Flavonoid metabolism; Quercetin degradation.	Expressed in roots and stems, and at lower levels in leaves.	aromatic compound biosynthetic process [GO:0019438]; flavonol biosynthetic process [GO:0051555]; lignin biosynthetic process [GO:0009809]; melatonin biosynthetic process [GO:0030187]; methylation [GO:0032259]	acetylserotonin O-methyltransferase activity [GO:0017096]; caffeate O-methyltransferase activity [GO:0047763]; luteolin O-methyltransferase activity [GO:0030744]; O-methyltransferase activity [GO:0008171]; protein dimerization activity [GO:0046983]; quercetin 3'-O-methyltransferase activity [GO:0102822]; S-adenosylmethionine-dependent methyltransferase activity [GO:0008757]	Methylates OH residues of flavonoid compounds. Can methylate eriodictyol, luteolin, quercetin and taxifolin. Methylates caffeate to produce ferrulate. Catalyzes the methylation of monolignols, the lignin precursors. Functions cooperatively with CAD2 in the culm internodes for the biosynthesis of monolignols. May be involved in lignin biosynthesis in leaves and roots. Involved in syringyl lignin biosynthesis. Can function as 5-hydroxyconiferaldehyde O-methyltransferase in the biosynthetic pathway to syringyl lignin. Involved in melatonin biosynthesis. Can function as acetylserotonin O-methyltransferase. Catalyzes the transfer of a methyl group onto N-acetylserotonin, producing melatonin (N-acetyl-5-methoxytryptamine).
V	COMT	Q7XXD4	*Oryza sativa* subsp. *japonica* (Rice)				aromatic compound biosynthetic process [GO:0019438]; methylation [GO:0032259]	O-methyltransferase activity [GO:0008171]; protein dimerization activity [GO:0046983]; S-adenosylmethionine-dependent methyltransferase activity [GO:0008757]	
V	COMT	Q7XXI9	*Oryza sativa* subsp. *japonica* (Rice)				aromatic compound biosynthetic process [GO:0019438]; methylation [GO:0032259]	O-methyltransferase activity [GO:0008171]; protein dimerization activity [GO:0046983]; S-adenosylmethionine-	

Group	Protein families	Entry	Organism	Catalytic activity	Pathway	Tissue specificity	Gene Ontology (biological process)	Gene Ontology (molecular function)	Function
								dependent methyltransferase activity [GO:0008757]	
V	COMT	Q84N28	*Triticum aestivum* (Wheat)				aromatic compound biosynthetic process [GO:0019438]; flavonoid biosynthetic process [GO:0009813]; methylation [GO:0032259]; response to ethylene [GO:0009723]; response to hydrogen peroxide [GO:0042542]; response to salicylic acid [GO:0009751]; response to wounding [GO:0009611]	O-methyltransferase activity [GO:0008171]; protein dimerization activity [GO:0046983]; S-adenosylmethionine-dependent methyltransferase activity [GO:0008757]	Flavone-specific O-methyltransferase with a preference for flavones > flavonols. Active with tricetin, luteolin, quercitin and eriodictyol. Very low activity with phenylpropanoids (5-hydroxyferulic acid and caffeic acid). Catalyzes the sequential O-methylation of tricetin via 3'-O-methyltricetin, 3',5'-O-methyltricetin to 3',4',5'-O-trimethyltricetin.
V	COMT	Q8GU25	*Rosa chinensis* (China rose)	(E)-caffeate + S-adenosyl-L-methionine = (E)-ferulate + H(+) + S-adenosyl-L-homocysteine	Aromatic compound metabolism; Phenylpropanoid biosynthesis.		lignin biosynthetic process [GO:0009809]; methylation [GO:0032259]	caffeate O-methyltransferase activity [GO:0047763]; protein dimerization activity [GO:0046983]	Catalyzes the conversion of caffeic acid to ferulic acid and of 5-hydroxyferulic acid to sinapic acid. The resulting products may subsequently be converted to the corresponding alcohols that are incorporated into lignins.
V	COMT	Q8LL87	*Coffea canephora* (Robusta coffee)	(E)-caffeate + S-adenosyl-L-methionine = (E)-ferulate + H(+) + S-adenosyl-L-homocysteine	Aromatic compound metabolism; Phenylpropanoid biosynthesis.		lignin biosynthetic process [GO:0009809]; methylation [GO:0032259]	caffeate O-methyltransferase activity [GO:0047763]; protein dimerization activity [GO:0046983]	Catalyzes the conversion of caffeic acid to ferulic acid and of 5-hydroxyferulic acid to sinapic acid. The resulting products may subsequently be converted to the corresponding alcohols that are incorporated into lignins.
V	COMT	Q8W013	*Catharanthus roseus* (Madagascar periwinkle) (*Vinca rosea*)	(E)-caffeate + S-adenosyl-L-methionine = (E)-ferulate + H(+) + S-adenosyl-L-homocysteine	Aromatic compound metabolism; Phenylpropanoid biosynthesis.		lignin biosynthetic process [GO:0009809]; methylation [GO:0032259]	caffeate O-methyltransferase activity [GO:0047763]; protein dimerization activity [GO:0046983]	Catalyzes the conversion of caffeic acid to ferulic acid and of 5-hydroxyferulic acid to sinapic acid. The resulting products may subsequently be converted to the corresponding alcohols that are incorporated into lignins.
V	COMT	Q9FK25	*Arabidopsis thaliana* (Mouse-ear cress)	a 3'-hydroxyflavone + S-adenosyl-L-methionine = a 3'-methoxyflavone + H(+) + S-adenosyl-L-homocysteine	Flavonoid metabolism; Quercetin degradation.; Aromatic compound metabolism; Melatonin biosynthesis; Melatonin from serotonin: step 1/2.	Expressed in seedlings, leaves, stems, flowers and siliques, mostly in vascular tissues. Mostly expressed in the apical part of the stems and in roots.	aromatic compound biosynthetic process [GO:0019438]; flavonol biosynthetic process [GO:0051555]; lignin biosynthetic process [GO:0009809]; melatonin biosynthetic process [GO:0030187]; methylation [GO:0032259]	acetylserotonin O-methyltransferase activity [GO:0017096]; caffeate O-methyltransferase activity [GO:0047763]; luteolin O-methyltransferase activity [GO:0030744]; myricetin 3'-O-	Methylates OH residues of flavonoid compounds. Converts quercetin into isorhamnetin. Dihydroquercetin is not a substrate. Catalyzes the methylation of monolignols, the lignin precursors. Does not contribute to the phenylpropanoid pattern of the pollen tryphine, but is probably confined to isorhamnetin glycoside biosynthesis. Involved in melatonin biosynthesis. Can function as acetylserotonin O-methyltransferase. Catalyzes the transfer of a methyl group onto N-

Group	Protein families	Entry	Organism	Catalytic activity	Pathway	Tissue specificity	Gene Ontology (biological process)	Gene Ontology (molecular function)	Function
						Expressed in the endothecium and the epidermal anther, but not in the tapetum. Also detected in all epidermal tissues of flower organs, including petals, sepals and the tip of the stigma.		methyltransferase activity [GO:0033799]; O-methyltransferase activity [GO:0008171]; protein dimerization activity [GO:0046983]; quercetin 3'-O-methyltransferase activity [GO:0102822]; quercetin 3-O-methyltransferase activity [GO:0030755]; S-adenosylmethionine-dependent methyltransferase activity [GO:0008757]	acetylserotonin, producing melatonin (N-acetyl-5-methoxytryptamine).
V	COMT	Q9FQY8	*Capsicum annuum* (Capsicum pepper)	(E)-caffeate + S-adenosyl-L-methionine = (E)-ferulate + H(+) + S-adenosyl-L-homocysteine	Aromatic compound metabolism; Phenylpropanoid biosynthesis.	Fruit. Not expressed in leaf.	lignin biosynthetic process [GO:0009809]; methylation [GO:0032259]	caffeate O-methyltransferase activity [GO:0047763]; protein dimerization activity [GO:0046983]	Catalyzes the conversion of caffeic acid to ferulic acid and of 5-hydroxyferulic acid to sinapic acid. The resulting products may subsequently be converted to the corresponding alcohols that are incorporated into lignins.
V	COMT	Q9SWC2	*Eucalyptus globulus* (Tasmanian blue gum)	(E)-caffeate + S-adenosyl-L-methionine = (E)-ferulate + H(+) + S-adenosyl-L-homocysteine	Aromatic compound metabolism; Phenylpropanoid biosynthesis.		lignin biosynthetic process [GO:0009809]; methylation [GO:0032259]	caffeate O-methyltransferase activity [GO:0047763]; protein dimerization activity [GO:0046983]	Catalyzes the conversion of caffeic acid to ferulic acid and of 5-hydroxyferulic acid to sinapic acid. The resulting products may subsequently be converted to the corresponding alcohols that are incorporated into lignins.
V	COMT	Q9XGV9	*Ocimum basilicum* (Sweet basil)	(E)-caffeate + S-adenosyl-L-methionine = (E)-ferulate + H(+) + S-adenosyl-L-homocysteine	Aromatic compound metabolism; Phenylpropanoid biosynthesis.		lignin biosynthetic process [GO:0009809]; methylation [GO:0032259]	caffeate O-methyltransferase activity [GO:0047763]; protein dimerization activity [GO:0046983]	Catalyzes the conversion of caffeic acid to ferulic acid and of 5-hydroxyferulic acid to sinapic acid. The resulting products may subsequently be converted to the corresponding alcohols that are incorporated into lignins.
V	COMT	Q9XGW0	*Ocimum basilicum* (Sweet basil)	(E)-caffeate + S-adenosyl-L-methionine = (E)-ferulate + H(+) + S-adenosyl-L-homocysteine	Aromatic compound metabolism; Phenylpropanoid biosynthesis.		lignin biosynthetic process [GO:0009809]; methylation [GO:0032259]	caffeate O-methyltransferase activity [GO:0047763]; protein dimerization activity [GO:0046983]	Catalyzes the conversion of caffeic acid to ferulic acid and of 5-hydroxyferulic acid to sinapic acid. The resulting products may subsequently be converted to the corresponding alcohols that are incorporated into lignins.
Outgroup I + II + III	COMT	A8QW51	*Sorghum bicolor* (Sorghum) (*Sorghum vulgare*)			Expressed predominantly in root hairs.	aromatic compound biosynthetic process [GO:0019438]; methylation [GO:0032259]	O-methyltransferase activity [GO:0008171]; protein dimerization activity [GO:0046983]; S-	O-methyltransferase of unknown substrate specificity. Not active on resorcinol, orcinol, guaiacol, eugenol, ferulic acid, p-coumaric acid, catechol, caffeic acid or monomethyl ethers of resorcinol or orcinol.

Group	Protein families	Entry	Organism	Catalytic activity	Pathway	Tissue specificity	Gene Ontology (biological process)	Gene Ontology (molecular function)	Function
								adenosylmethionine-dependent methyltransferase activity [GO:0008757]	
Outgroup I + II + III	COMT	A8QW53	*Sorghum bicolor* (Sorghum) (*Sorghum vulgare*)	(8Z,11Z)-5-(pentadeca-8,11,14-trien-1-yl)resorcinol + S-adenosyl-L-methionine = (8Z,11Z)-5-(pentadeca- 8,11,14-trien-1-yl)resorcinol-3-methyl ether + H(+) + S-adenosyl-L-homocysteine		Expressed predominantly in root hairs.	aromatic compound biosynthetic process [GO:0019438]; methylation [GO:0032259]	5-n-alk(en)ylresorcinol O-methyltransferase activity [GO:0102990]; O-methyltransferase activity [GO:0008171]; protein dimerization activity [GO:0046983]; S-adenosylmethionine-dependent methyltransferase activity [GO:0008757]	O-methyltransferase involved in the biosynthetic pathway of the phytotoxin sorgoleone, a potent broad-spectrum inhibitor active against many agronomically important monocot and dicot weed species. Substrate specificity for alkylresorcinols. Strong preference for a five carbons alkyl side chain.
Outgroup I + II + III	COMT	B1P123	*Zea mays* (Maize)	S-adenosyl-L-methionine + TRIBOA beta-D-glucoside = DIMBOA beta-D-glucoside + H(+) + S-adenosyl-L-homocysteine		Expressed in seedlings and newly formed crown roots. Highest expression in the scutellar node. Low to non detectable levels in cob, tassel and mature organs like husk or leaves.	aromatic compound biosynthetic process [GO:0019438]; methylation [GO:0032259]	O-methyltransferase activity [GO:0008171]; protein dimerization activity [GO:0046983]; S-adenosylmethionine-dependent methyltransferase activity [GO:0008757]; TRIBOA-glucoside methyltransferase activity [GO:0102718]	O-methyltransferase involved in the benzoxazinoid glucoside biosynthesis. Can use 2,4,7-trihydroxy-2H-1,4-benzoxazin-3(4H)-one 2-D-glucoside (TRIBOA-glucoside) as substrate, but not aglucone TRIBOA, caffeic acid, ferulic acid, apigenin or quercetin.
Outgroup I + II + III	COMT	P47917	*Zea mays* (Maize)			Accumulates preferentially in the roots and is located predominantly in the region of the endodermis, low levels are seen in the leaves, stems and other shoot organs.	aromatic compound biosynthetic process [GO:0019438]; methylation [GO:0032259]	O-methyltransferase activity [GO:0008171]; protein dimerization activity [GO:0046983]; S-adenosylmethionine-dependent methyltransferase activity [GO:0008757]	May be involved in the O-methylation of suberin phenylpropanoid precursors.
Outgroup I + II + III	COMT	Q53QK0	*Oryza sativa* subsp. *japonica* (Rice)				aromatic compound biosynthetic process [GO:0019438]; methylation [GO:0032259]	O-methyltransferase activity [GO:0008171]; protein dimerization activity	

Group	Protein families	Entry	Organism	Catalytic activity	Pathway	Tissue specificity	Gene Ontology (biological process)	Gene Ontology (molecular function)	Function
								[GO:0046983]; S-adenosylmethionine-dependent methyltransferase activity [GO:0008757]	
Outgroup I + II + III	COMT	Q5C9L7	*Thalictrum flavum* subsp. *glaucum* (Yellow meadow rue)	(S)-norcoclaurine + S-adenosyl-L-methionine = (S)-coclaurine + H(+) + S-adenosyl-L-homocysteine		Expressed in leaf primordia of rhizomes and root endodermis.	alkaloid metabolic process [GO:0009820]; methylation [GO:0032259]	(RS)-norcoclaurine 6-O-methyltransferase activity [GO:0030786]; O-methyltransferase activity [GO:0008171]; protein dimerization activity [GO:0046983]	Involved in the biosynthesis of coclaurine, a precursor of benzylisoquinoline alkaloids. Catalyzes the transfer of the S-methyl group of S-adenosyl-L-methionine (AdoMet) to the 6-hydroxyl group of norcoclaurine to form coclaurine.
Outgroup I + II + III	COMT	Q84XW5	*Secale cereale* (Rye)	7,8-dihydroxycoumarin + S-adenosyl-L-methionine = 7-hydroxy-8-methoxycoumarin + H(+) + S-adenosyl-L-homocysteine	Aromatic compound metabolism; Secondary metabolite biosynthesis.		aromatic compound biosynthetic process [GO:0019438]; methylation [GO:0032259]; response to cold [GO:0009409]; response to high light intensity [GO:0009644]; secondary metabolite biosynthetic process [GO:0044550]	daphnetin-8-O-methyltransferase activity [GO:0102358]; O-methyltransferase activity [GO:0008171]; protein dimerization activity [GO:0046983]; S-adenosylmethionine-dependent methyltransferase activity [GO:0008757]	O-methyltransferase involved in the biosynthesis of coumarins natural products such as daphnetin derivatives. Catalyzes specifically the methylation of daphnetin (7,8-dihydroxycoumarin) to produce hydrangetin (7-hydroxy-8-methoxycoumarin). Probably involved in acclimation to low temperature conditions.
Outgroup IV + V	COMT	Q8H9A8	*Coptis japonica* (Japanese goldthread)	columbamine + S-adenosyl-L-methionine = H(+) + palmatine + S-adenosyl-L-homocysteine	Alkaloid biosynthesis; Palmatine biosynthesis; Palmatine from columbamine: step 1/1.		alkaloid metabolic process [GO:0009820]; methylation [GO:0032259]	columbamine O-methyltransferase activity [GO:0030778]; O-methyltransferase activity [GO:0008171]; protein dimerization activity [GO:0046983]; tetrahydrocolumbamine 2-O-methyltransferase activity [GO:0030762]	Catalyzes the conversion of tetrahydrocolumbamine to (S)-tetrahydropalmatine and of columbamine to palmatine, an isoquinoline alkaloid.
Outgroup IV + V	COMT	A0A2S1WBY6	*Papaver somniferum* (Opium poppy)	(S)-scoulerine + S-adenosyl-L-methionine = (S)-tetrahydrocolumbamine + H(+) + S-adenosyl-L-homocysteine	Alkaloid biosynthesis.		alkaloid metabolic process [GO:0009820]; methylation [GO:0032259]	(S)-scoulerine 9-O-methyltransferase activity [GO:0030777]; O-methyltransferase activity [GO:0008171]; protein dimerization activity [GO:0046983]	Methyltransferase involved in the biosynthesis of the benzylisoquinoline alkaloid noscapine (By similarity). Catalyzes the conversion of (S)-scoulerine to (S)-tetrahydrocolumbamine (By similarity). The heterodimers OMT2B-SOMT3 and OMT2B-6OMT do not possess 3-O-

Group	Protein families	Entry	Organism	Catalytic activity	Pathway	Tissue specificity	Gene Ontology (biological process)	Gene Ontology (molecular function)	Function
									acetyl-4'-O-demethylpapaveroxine 4'-O-methyltransferase activity.
Outgroup IV + V	COMT	A0A2S1WC15	*Papaver somniferum* (Opium poppy)	(S)-scoulerine + S-adenosyl-L-methionine = (S)-tetrahydrocolumbamine + H(+) + S-adenosyl-L-homocysteine	Alkaloid biosynthesis.		alkaloid metabolic process [GO:0009820]; methylation [GO:0032259]	(S)-scoulerine 9-O-methyltransferase activity [GO:0030777]; O-methyltransferase activity [GO:0008171]; protein dimerization activity [GO:0046983]	Methyltransferase involved in the biosynthesis of the benzylisoquinoline alkaloid noscapine (By similarity). Catalyzes the conversion of (S)-scoulerine to (S)-tetrahydrocolumbamine (By similarity).
Outgroup IV + V	COMT	I3PLQ6	*Papaver somniferum* (Opium poppy)	(S)-scoulerine + S-adenosyl-L-methionine = (S)-tetrahydrocolumbamine + H(+) + S-adenosyl-L-homocysteine	Alkaloid biosynthesis.	Highly expressed in capsules. Expressed is stems. Expressed at low levels in roots.	alkaloid metabolic process [GO:0009820]; methylation [GO:0032259]	(S)-scoulerine 9-O-methyltransferase activity [GO:0030777]; O-methyltransferase activity [GO:0008171]; protein dimerization activity [GO:0046983]	Methyltransferase involved in the biosynthesis of the benzylisoquinoline alkaloid noscapine. Catalyzes the conversion of (S)-scoulerine to (S)-tetrahydrocolumbamine. The heterodimers SOMT2-SOMT3 and SOMT2-6OMT convert 3-O-acetyl-4'-O-demethylpapaveroxine to 3-O-acetylpapaveroxine, where SOMT2 is the catalytic subunit.
Outgroup IV + V	COMT	I3PLQ7	*Papaver somniferum* (Opium poppy)	(S)-scoulerine + S-adenosyl-L-methionine = (S)-tetrahydrocolumbamine + H(+) + S-adenosyl-L-homocysteine	Alkaloid biosynthesis.	Highly expressed in capsules. Expressed is stems. Expressed at low levels in roots.	alkaloid metabolic process [GO:0009820]; methylation [GO:0032259]	(S)-scoulerine 9-O-methyltransferase activity [GO:0030777]; O-methyltransferase activity [GO:0008171]; protein dimerization activity [GO:0046983]	Methyltransferase involved in the biosynthesis of the benzylisoquinoline alkaloid noscapine. Catalyzes the conversion of (S)-scoulerine to (S)-tetrahydrocolumbamine.
Outgroup IV + V	COMT	Q6T1F5	*Ammi majus* (Bishop's weed)	(E)-caffeate + S-adenosyl-L-methionine = (E)-ferulate + H(+) + S-adenosyl-L-homocysteine	Aromatic compound metabolism; Phenylpropanoid biosynthesis.		lignin biosynthetic process [GO:0009809]; methylation [GO:0032259]	caffeate O-methyltransferase activity [GO:0047763]; protein dimerization activity [GO:0046983]	Catalyzes the conversion of caffeic acid to ferulic acid and of 5-hydroxyferulic acid to sinapic acid. The resulting products may subsequently be converted to the corresponding alcohols that are incorporated into lignins.
Outgroup IV + V	COMT	Q7XB10	*Papaver somniferum* (Opium poppy)	(S)-3'-hydroxy-N-methylcoclaurine + S-adenosyl-L-methionine = (S)-reticuline + H(+) + S-adenosyl-L-homocysteine	Alkaloid biosynthesis; (S)-reticuline biosynthesis; (S)-reticuline from (S)-norcoclaurine: step 4/4.	Expressed in roots, stems, leaves and flowers.	alkaloid metabolic process [GO:0009820]; methylation [GO:0032259]	3'-hydroxy-N-methyl-(S)-coclaurine 4'-O-methyltransferase activity [GO:0030784]; O-methyltransferase activity [GO:0008171]; protein dimerization activity [GO:0046983]	Involved in the biosynthesis of benzylisoquinoline alkaloids. Catalyzes the transfer of the methyl group to the 4'-hydroxyl group of 3'-hydroxy-N-methylcoclaurine to form reticuline. Can also use laudanosoline and, with a lower activity, 6-O-methylnorlaudanosoline and norlaudanosoline as substrates. Also involved in the papaverine biosynthesis.
Outgroup IV + V	COMT	Q7XB11	*Papaver somniferum* (Opium poppy)	(S)-3'-hydroxy-N-methylcoclaurine + S-adenosyl-L-methionine = (S)-reticuline + H(+) +	Alkaloid biosynthesis; (S)-reticuline biosynthesis; (S)-reticuline	Expressed in roots, stems, leaves and flowers. Restricted to	alkaloid metabolic process [GO:0009820]; methylation [GO:0032259]	3'-hydroxy-N-methyl-(S)-coclaurine 4'-O-methyltransferase activity [GO:0030784]; O-	Involved in the biosynthesis of benzylisoquinoline alkaloids. Catalyzes the transfer of the methyl group to the 4'-hydroxyl group of 3'-hydroxy-N-methylcoclaurine to form reticuline.

Group	Protein families	Entry	Organism	Catalytic activity	Pathway	Tissue specificity	Gene Ontology (biological process)	Gene Ontology (molecular function)	Function
				S-adenosyl-L-homocysteine	from (S)-norcoclaurine: step 4/4.	sieve elements of the phloem adjacent or proximal to laticifers.		methyltransferase activity [GO:0008171]; protein dimerization activity [GO:0046983]	Also involved in the papaverine biosynthesis.
Outgroup IV + V	COMT	Q9LEL5	*Coptis japonica* (Japanese goldthread)	(S)-3'-hydroxy-N-methylcoclaurine + S-adenosyl-L-methionine = (S)-reticuline + H(+) + S-adenosyl-L-homocysteine	Alkaloid biosynthesis; (S)-reticuline biosynthesis; (S)-reticuline from (S)-norcoclaurine: step 4/4.		methylation [GO:0032259]	3'-hydroxy-N-methyl-(S)-coclaurine 4'-O-methyltransferase activity [GO:0030784]; O-methyltransferase activity [GO:0008171]; protein dimerization activity [GO:0046983]	Catalyzes the transfer of the methyl group to the 4'-hydroxyl group of 3'-hydroxy-N-methylcoclaurine to form reticuline.
Outgroup IV + V	COMT	Q9LEL6	*Coptis japonica* (Japanese goldthread)	norcoclaurine + S-adenosyl-L-methionine = coclaurine + H(+) + S-adenosyl-L-homocysteine	Alkaloid biosynthesis; (S)-reticuline biosynthesis; (S)-reticuline from (S)-norcoclaurine: step 1/4.		methylation [GO:0032259]	(RS)-norcoclaurine 6-O-methyltransferase activity [GO:0030786]; O-methyltransferase activity [GO:0008171]; protein dimerization activity [GO:0046983]	Catalyzes the transfer of the S-methyl group of S-adenosyl-L-methionine (AdoMet) to the 6-hydroxyl group of norcoclaurine to form coclaurine.
