# Supplementary material for: Evolution and diversification of the O-methyltransferase (OMT) gene family in Solanaceae
Source: Genet Mol Biol. 2023 Nov 10;46(3 Suppl 1):e20230121. doi: 10.1590/1678-4685-GMB-2023-0121 (PMC10637433; doi:10.1590/1678-4685-GMB-2023-0121)
Supplement: Table S3 - [file 1415-4757-GMB-46-3-s1-e20230121-s3.pdf]

Genetics and Molecular Biology
https://doi.org/10.1590/1678-4685-GMB-2023-0121

Supplementary Material to "Evolution and diversification of the O-methyltransferase (OMT) gene family in Solanaceae"

Table S3 - Structural and molecular characteristics of OMT proteins of six Solanaceae representative species.

Species	Subfamily	Original Gene ID	Putative Name	Protein length	Intron numbers	Mw (kDa)	pI	Chromosomal location
Capsicum annuum	CCoAOMT	CA02g14460	Can_CCoAOMT3_c2	242	3	27.23	5.30	2
		CA00g52190	Can_CCoAOMT8_c0	247	4	27.82	5.30	0
		CA02g14450	Can_CCoAOMT2_c2	242	3	27.25	5.29	2
		CA02g14470	Can_CCoAOMT4_c2	242	3	27.23	5.28	2
		CA04g12060	Can_CCoAOMT6_c4	234	1	26.42	5.07	4
		CA04g05360	Can_CCoAOMT5_c4	235	4	26.53	5.45	4
		CA01g32140	Can_CCoAOMT1_c1	288	8	32.26	8.42	1
		CA04g05330	-	143	-	-	-	4
		CA04g05310	-	134	-	-	-	4
		CA00g18340	-	117	-	-	-	0
		CA08g08550	Can_CCoAOMT7_c8	250	1	27.52	8.72	8
	COMT	CA03g21160	Can_COMT13_c3	329	3	35.87	5.40	3
		CA03g21170	Can_COMT14_c3	204	2	22.56	5.97	3
		CA10g05120	Can_COMT31_c10	362	3	40.23	5.58	10
		CA09g13730	Can_COMT27_c9	355	2	39.86	5.43	9
		CA06g11300	Can_COMT24_c6	366	3	40.58	5.71	6
		CA03g22390	Can_COMT15_c3	354	1	39.04	5.12	3
		CA10g02820	Can_COMT30_c10	361	1	40.60	5.59	10
		CA06g28400	Can_COMT26_c6	374	3	41.66	5.61	6

Species	Subfamily	Original Gene ID	Putative Name	Protein length	Intron numbers	Mw (kDa)	pI	Chromosomal location
		CA03g22470	Can_COMT17_c3	282	1	31.39	5.27	3
		CA02g04190	Can_COMT8_c2	353	1	39.66	5.48	2
		CA03g21100	Can_COMT12_c3	205	3	22.18	6.59	3
		CA10g13670	Can_COMT34_c10	372	1	42.00	5.11	10
		CA06g28390	Can_COMT25_c6	364	3	40.77	5.76	6
		CA00g52690	Can_COMT38_c0	359	2	39.38	5.65	0
		CA00g52700	Can_COMT39_c0	363	2	40.35	5.59	0
		CA06g05520	Can_COMT23_c6	366	1	41.29	5.72	6
		CA01g32000	Can_COMT7_c1	351	1	39.49	5.48	1
		CA10g13680	Can_COMT35_c10	346	1	38.89	5.69	10
		CA10g20040	Can_COMT36_c10	359	1	40.94	5.26	10
		CA01g17250	Can_COMT3_c1	282	1	31.73	6.25	1
		CA01g22090	Can_COMT4_c1	352	1	39.55	5.47	1
		CA02g04210	Can_COMT9_c2	342	1	38.31	5.82	2
		CA09g17130	Can_COMT28_c9	375	2	41.26	5.44	9
		CA03g22510	-	180	-	-	-	3
		CA03g22410	-	179	-	-	-	3
		CA02g28620	-	185	-	-	-	2
		CA03g22550	-	188	-	-	-	3
		CA03g22570	-	166	-	-	-	3
		CA04g17400	Can_COMT21_c4	372	1	42.70	5.26	4
		CA01g22160	Can_COMT6_c1	290	1	32.00	6.49	1
		CA10g13660	Can_COMT33_c10	318	2	35.85	5.76	10
		CA03g21120	-	124	-	-	-	3
		CA03g18440	-	135	-	-	-	3
		CA03g22430	Can_COMT16_c3	276	0	30.73	5.33	3
		CA03g22450	-	183	-	-	-	3
		CA01g17240	Can_COMT2_c1	243	1	27.35	4.98	1
		CA03g22560	-	129	-	-	-	3
		CA03g21110	-	101	-	-	-	3

Species	Subfamily	Original Gene ID	Putative Name	Protein length	Intron numbers	Mw (kDa)	pI	Chromosomal location
		CA02g09700	-	140	-	-	-	2
		CA02g13190	-	114	-	-	-	2
		CA03g22440	-	105	-	-	-	3
		CA06g11270	-	101	-	-	-	6
		CA03g18560	Can_COMT11_c3	249	0	27.64	5.05	3
		CA04g03650	-	132	-	-	-	4
		CA03g18570	-	119	-	-	-	3
		CA10g12170	Can_COMT32_c10	287	1	31.85	6.25	10
		CA10g12160	-	102	-	-	-	10
		CA00g13690	-	182	-	-	-	0
		CA03g22540	-	97	-	-	-	3
		CA01g22100	Can_COMT5_c1	217	0	24.45	5.58	1
		CA03g22500	Can_COMT18_c3	207	0	23.50	5.60	3
		CA06g05760	-	60	-	-	-	6
		CA05g14270	-	93	-	-	-	5
		CA06g10890	-	70	-	-	-	6
		CA03g32710	Can_COMT19_c3	486	10	55.02	5.79	3
		CA03g22490	-	71	-	-	-	3
		CA12g11540	Can_COMT37_c12	488	10	56.01	5.82	12
		CA10g02660	Can_COMT29_c10	214	4	24.16	6.52	10
		CA03g35560	Can_COMT20_c3	272	7	31.01	9.17	3
		CA03g22520	-	175	-	-	-	3
		CA06g01600	Can_COMT22_c6	434	4	48.82	6.08	6
		CA01g06010	Can_COMT1_c1	230	8	26.14	5.36	1
		CA03g09680	Can_COMT10_c3	339	2	38.11	6.40	3
		CA03g22530	-	62	-	-	-	3
Datura stramonium	CCoAOMT	MCE3215619	Dst_CCoAOMT1_c0	242	5	27.30	5.30	0
		MCD9645083	Dst_CCoAOMT2_c0	247	4	27.68	5.41	0
		MCD9558573	Dst_CCoAOMT3_c0	437	11	49.93	5.57	0
		MCE3050740	Dst_CCoAOMT4_c0	282	2	31.95	4.87	0

Species	Subfamily	Original Gene ID	Putative Name	Protein length	Intron numbers	Mw (kDa)	pI	Chromosomal location
		MCD7465610	Dst_CCoAOMT5_c0	288	8	32.34	8.18	0
		MCD7453179	-	175	-	-	-	-
		MCD7453181	-	176	-	-	-	-
		MCD7449240	Dst_CCoAOMT6_c0	185	3	20.87	6.30	0
		MCD7453177	-	154	-	-	-	-
		MCD7453182	-	87	-	-	-	-
		MCD7453180	-	142	-	-	-	-
		MCE3214678	Dst_CCoAOMT7_c0	251	1	27.60	8.81	0
	COMT	MCD7461403	Dst_COMT1_c0	361	3	39.61	5.63	0
		MCD9644131	Dst_COMT2_c0	366	3	40.36	5.63	0
		MCD7462215	Dst_COMT3_c0	356	2	39.98	5.49	0
		MCD7467906	Dst_COMT4_c0	365	1	41.16	5.24	0
		MCD7462214	Dst_COMT5_c0	356	2	39.78	5.82	0
		MCD7445861	Dst_COMT6_c0	359	1	40.14	5.67	0
		MCE5166507	Dst_COMT7_c0	366	1	40.91	5.45	0
		MCD7460042	Dst_COMT8_c0	370	1	41.46	6.06	0
		MCD9637567	Dst_COMT9_c0	361	1	40.62	5.64	0
		MCD7471929	Dst_COMT10_c0	360	1	40.57	5.23	0
		MCE3214919	Dst_COMT11_c0	423	3	47.44	6.47	0
		MCD7460043	Dst_COMT12_c0	369	1	41.40	5.58	0
		MCD7459910	Dst_COMT13_c0	352	1	39.35	5.44	0
		MCD7446510	Dst_COMT14_c0	372	1	41.75	6.23	0
		MCD7453195	Dst_COMT15_c0	353	1	39.29	5.72	0
		MCD7472334	Dst_COMT16_c0	358	1	40.87	4.70	0
		MCE3052455	Dst_COMT17_c0	361	3	40.36	5.76	0
		MCD7470164	Dst_COMT18_c0	310	1	34.12	5.31	0
		MCD7467907	Dst_COMT19_c0	322	1	36.24	5.88	0
		MCD7467905	Dst_COMT20_c0	311	3	34.93	5.03	0
		MCD7451351	Dst_COMT21_c0	353	2	38.91	5.85	0
		MCD7452308	Dst_COMT22_c0	303	2	34.37	5.77	0

Species	Subfamily	Original Gene ID	Putative Name	Protein length	Intron numbers	Mw (kDa)	pI	Chromosomal location
		MCD9560098	-	125	-	-	-	0
		MCE3214920	Dst_COMT23_c0	272	1	30.40	5.43	0
		MCD7463306	Dst_COMT24_c0	332	3	36.82	5.42	0
		MCD7460044	Dst_COMT25_c0	258	1	28.46	6.25	0
		MCD7460041	Dst_COMT26_c0	238	3	26.78	5.97	0
		MCD7449260	-	171	-	-	-	0
		MCD7471930	-	143	-	-	-	0
		MCD7455183	Dst_COMT28_c0	219	1	25.16	5.80	0
		MCE3049744	Dst_COMT29_c0	312	2	35.08	6.00	0
		MCE3216138	-	80	-	-	-	0
		MCD9646786	-	128	-	-	-	0
		MCD7462336	-	143	-	-	-	0
		MCD7462337	-	166	-	-	-	0
		MCD9643151	Dst_COMT30_c0	565	12	63.87	5.94	0
		MCD9560190	-	147	-	-	-	0
		MCE0481912	Dst_COMT31_c0	491	11	55.55	5.62	0
		MCD7448295	Dst_COMT32_c0	214	4	24.29	6.97	0
		MCD7446806	Dst_COMT33_c0	531	10	61.06	9.08	0
		MCD9639620	Dst_COMT27_c0	339	2	38.26	7.05	0
Iochroma cyaneum	CCoAOMT	IC12g008860	Icy_CCoAOMT9_c12	685	16	77.64	5.63	12
		IC10g022870	Icy_CCoAOMT7_c10	248	4	27.94	5.30	10
		IC02g033430	Icy_CCoAOMT1_c2	242	3	27.26	5.29	2
		IC02g033460	Icy_CCoAOMT4_c2	242	3	27.23	5.29	2
		IC02g033440	Icy_CCoAOMT2_c2	242	3	27.35	5.42	2
		IC02g033450	Icy_CCoAOMT3_c2	242	3	27.27	5.29	2
		scaffold20274s005110	Icy_CCoAOMT10_c0	247	4	27.85	5.31	0
		IC09g024120	Icy_CCoAOMT6_c9	236	5	26.25	6.10	9
		IC11g019030	Icy_CCoAOMT8_c11	270	1	30.51	4.90	11
		scaffold00612s005040	Icy_CCoAOMT11_c0	270	1	30.57	4.84	0
		scaffold00612s005020	Icy_CCoAOMT12_c0	284	1	31.91	4.91	0

Species	Subfamily	Original Gene ID	Putative Name	Protein length	Intron numbers	Mw (kDa)	pI	Chromosomal location
		scaffold20557s005040	Icy_CCoAOMT13_c0	327	10	36.84	6.37	0
		IC12g008870	-	159	-	-	-	12
		IC05g023050	Icy_CCoAOMT5_c5	190	3	21.26	7.83	5
		IC12g013510	-	139	-	-	-	12
		scaffold20310s005030	Icy_CCoAOMT14_c0	269	1	29.70	8.95	0
		scaffold20217s005160	Icy_COMT39_c0	361	3	39.36	5.75	0
		IC10g030960	Icy_COMT18_c10	362	3	40.06	5.48	10
		IC06g022690	Icy_COMT12_c6	373	4	40.88	5.42	6
		IC05g012690	Icy_COMT8_c5	355	2	39.84	5.52	5
		scaffold20189s005110	Icy_COMT38_c0	361	1	39.69	5.68	0
		IC02g022290	Icy_COMT5_c2	541	2	60.59	6.93	2
		scaffold20188s005040	Icy_COMT28_c0	356	1	39.71	5.67	0
		scaffold20189s005010	Icy_COMT33_c0	367	1	41.19	5.57	0
		IC02g022270	Icy_COMT3_c2	360	1	40.67	5.51	2
		scaffold20248s005580	Icy_COMT40_c0	360	2	39.64	5.78	0
		IC08g010850	Icy_COMT14_c8	357	1	40.23	5.81	8
		scaffold20248s005590.1	Icy_COMT41_c0	458	3	50.39	5.88	0
	COMT	scaffold20188s005050	Icy_COMT29_c0	360	1	40.57	5.81	0
		scaffold20188s005070	Icy_COMT31_c0	355	1	39.79	4.98	0
		IC12g009210	Icy_COMT21_c12	313	2	35.33	5.32	12
		scaffold20188s005090	Icy_COMT32_c0	376	1	41.75	5.12	0
		scaffold01852s005090	Icy_COMT24_c0	358	1	40.72	5.01	0
		IC12g009230	Icy_COMT22_c12	316	2	35.74	5.25	12
		IC01g024620	Icy_COMT2_c1	353	1	39.55	5.81	1
		IC01g024610	Icy_COMT1_c1	352	1	39.29	5.30	1
		IC06g018570	Icy_COMT11_c6	360	1	39.95	5.45	6
		IC05g006900	Icy_COMT6_c5	370	2	40.59	5.33	5
		IC10g034750	Icy_COMT20_c10	344	4	38.43	6.16	10
		scaffold20189s005030	Icy_COMT35_c0	327	2	36.80	5.15	0
		IC10g015090	Icy_COMT16_c10	360	3	40.09	5.52	10

Species	Subfamily	Original Gene ID	Putative Name	Protein length	Intron numbers	Mw (kDa)	pI	Chromosomal location
		IC06g040460	Icy_COMT13_c6	380	4	43.33	7.13	6
		scaffold20189s005100	Icy_COMT37_c0	361	1	39.81	5.27	0
		scaffold20189s005050	Icy_COMT36_c0	360	1	39.82	5.28	0
		scaffold20189s005020	Icy_COMT34_c0	327	2	36.72	5.08	0
		IC05g007100	Icy_COMT7_c5	421	6	47.44	6.70	5
		IC10g015100	Icy_COMT17_c10	360	3	40.04	5.73	10
		IC12g009200	-	184	-	-	-	12
		scaffold20188s005060	Icy_COMT30_c0	323	2	36.59	5.95	0
		IC02g022280	Icy_COMT4_c2	308	2	35.20	5.09	2
		scaffold20188s005030	-	154	-	-	-	-
		IC06g015000	Icy_COMT10_c6	267	2	30.26	5.43	6
		IC12g009240	-	194	-	-	-	-
		scaffold08911s005030	Icy_COMT25_c0	228	1	25.31	6.52	0
		IC05g012700	-	97	-	-	-	-
		scaffold20189s005070	-	123	-	-	-	-
		IC12g019070	Icy_COMT23_c12	499	11	56.70	5.79	12
		IC06g005920	Icy_COMT9_c6	492	11	56.21	5.29	6
		scaffold20106s005210	Icy_COMT27_c0	516	12	58.59	5.68	0
		scaffold20094s005280	Icy_COMT26_c0	279	8	31.78	8.82	0
		IC09g018990	Icy_COMT15_c9	339	2	38.02	7.05	9
		IC10g034590	Icy_COMT19_c10	392	7	43.90	5.96	10
Nicotiana attenuata	CCoAOMT	NIATv7_g57887	Nat_CCOAOMT6_c0	240	4	27.16	5.42	0
		NIATv7_g63611	Nat_CCOAOMT9_c0	247	4	27.77	5.30	0
		NIATv7_g04602	Nat_CCOAOMT1_c8	242	4	27.28	5.30	8
		NIATv7_g33225	Nat_CCOAOMT4_c0	235	4	26.48	5.33	0
		NIATv7_g26590	Nat_CCOAOMT3_c0	291	5	32.91	5.02	0
		NIATv7_g27763	Nat_CCOAOMT2_c9	230	4	25.48	5.40	9
		NIATv7_g41766	Nat_CCOAOMT5_c0	287	8	32.11	7.07	0
		NIATv7_g58236	Nat_CCOAOMT7_c0	287	8	32.11	7.07	0
		NIATv7_g61982	Nat_CCOAOMT8_c0	287	8	32.11	7.07	0

Species	Subfamily	Original Gene ID	Putative Name	Protein length	Intron numbers	Mw (kDa)	pI	Chromosomal location
		NIATv7_g59821	-	138	-	-	-	-
		NIATv7_g18253	Nat_COMT3_c2	461	3	52.19	5.06	2
		NIATv7_g31802	Nat_COMT7_c5	363	3	39.62	5.60	5
		NIATv7_g36761	Nat_COMT23_c0	363	3	40.15	5.43	0
		NIATv7_g63462	Nat_COMT27_c0	363	3	40.27	5.52	0
		NIATv7_g62877	Nat_COMT2_c1	273	4	30.83	6.75	1
		NIATv7_g38331	Nat_COMT25_c0	362	3	40.38	5.76	0
		NIATv7_g11825	Nat_COMT13_c0	388	2	42.99	5.06	0
		NIATv7_g03214	Nat_COMT5_c2	363	3	40.58	6.01	2
		NIATv7_g37802	Nat_COMT24_c0	363	1	40.78	5.56	0
		NIATv7_g32470	Nat_COMT18_c0	303	1	34.2	5.73	0
		NIATv7_g41648	Nat_COMT9_c7	353	1	39.51	6.15	7
		NIATv7_g20232	Nat_COMT14_c0	361	2	39.6	5.53	0
		NIATv7_g21941	Nat_COMT11_c8	356	3	39.32	6.26	8
		NIATv7_g60131	Nat_COMT10_c7	351	1	39.07	5.87	7
	COMT	NIATv7_g32756	Nat_COMT19_c0	351	1	39.57	5.65	0
		NIATv7_g21123	Nat_COMT8_c6	361	1	40.44	5.65	6
		NIATv7_g23365	Nat_COMT15_c0	363	3	40.25	5.96	0
		NIATv7_g02388	Nat_COMT12_c0	311	1	34.96	6.19	0
		NIATv7_g40724	Nat_COMT1_c1	295	3	32.33	5.51	1
		NIATv7_g34187	Nat_COMT21_c0	230	0	25.54	5.44	0
		NIATv7_g18251	Nat_COMT4_c2	553	2	61.17	6.02	2
		NIATv7_g18252	-	184	-	-	-	-
		NIATv7_g27322	Nat_COMT17_c0	362	4	39.33	4.83	0
		NIATv7_g57019	-	102	-	-	-	-
		NIATv7_g36103	Nat_COMT22_c0	496	11	56.32	5.11	0
		NIATv7_g55561	Nat_COMT26_c0	501	11	56.79	5.44	0
		NIATv7_g24204	Nat_COMT16_c0	279	8	31.66	9.25	0
		NIATv7_g10935	Nat_COMT6_c3	491	11	55.59	5.53	3
		NIATv7_g32988	Nat_COMT20_c0	339	2	38.15	8.21	0

Species	Subfamily	Original Gene ID	Putative Name	Protein length	Intron numbers	Mw (kDa)	pI	Chromosomal location
Petunia axillaris	CCoAOMT	Peaxi162Scf00450g00032	Pax_CCoAOMT1_c0	484	9	54.46	5.37	0
		Peaxi162Scf00003g02440	Pax_CCoAOMT2_c0	351	6	39.93	5.39	0
		Peaxi162Scf00016g02023	Pax_CCoAOMT3_c0	248	4	27.86	5.30	0
		Peaxi162Scf00518g00430	Pax_CCoAOMT4_c0	264	5	29.58	5.36	0
		Peaxi162Scf01363g00001	Pax_CCoAOMT5_c0	288	0	32.64	5.60	0
		Peaxi162Scf00045g02123	Pax_CCoAOMT6_c0	342	9	38.55	6.86	0
		Peaxi162Scf00316g00055	Pax_CCoAOMT7_c0	229	4	25.46	5.02	0
		Peaxi162Scf00461g00810	-	145	-	-	-	0
		Peaxi162Scf00089g00427	-	90	-	-	-	0
		Peaxi162Scf00461g00091	-	94	-	-	-	0
		Peaxi162Scf00786g00014	Pax_CCoAOMT8_c0	247	1	27.28	8.74	0
	COMT	Peaxi162Scf00078g00836	Pax_COMT1_c0	888	4	99.34	5.91	0
		Peaxi162Scf00092g00172	Pax_COMT2_c0	568	6	63.00	5.85	0
		Peaxi162Scf00078g00930	Pax_COMT3_c0	708	4	79.49	5.95	0
		Peaxi162Scf01698g00013	Pax_COMT4_c0	431	5	47.63	5.46	0
		Peaxi162Scf00912g00111	Pax_COMT5_c0	359	3	39.36	5.52	0
		Peaxi162Scf00401g00617	Pax_COMT6_c0	361	3	39.92	5.29	0
		Peaxi162Scf00313g00016	Pax_COMT7_c0	363	3	40.03	5.36	0
		Peaxi162Scf01122g00018	Pax_COMT8_c0	357	1	39.79	5.54	0
		Peaxi162Scf00517g00518	Pax_COMT9_c0	342	4	37.52	5.66	0
		Peaxi162Scf00241g00135	Pax_COMT10_c0	359	1	40.30	5.75	0
		Peaxi162Scf00517g00634	Pax_COMT11_c0	339	3	37.92	5.66	0
		Peaxi162Scf00240g00028	Pax_COMT12_c0	361	1	40.24	5.45	0
		Peaxi162Scf00078g00926	Pax_COMT13_c0	370	1	41.44	5.75	0
		Peaxi162Scf00657g00004	Pax_COMT14_c0	357	1	40.56	5.31	0
		Peaxi162Scf00034g00003	Pax_COMT15_c0	357	1	40.53	5.31	0
		Peaxi162Scf00034g00193	Pax_COMT16_c0	370	1	41.86	5.74	0
		Peaxi162Scf00362g00342	Pax_COMT17_c0	362	2	40.02	5.97	0
		Peaxi162Scf00401g00526	Pax_COMT18_c0	353	2	39.12	5.94	0
		Peaxi162Scf00010g00018	Pax_COMT19_c0	366	1	41.23	6.14	0
		Peaxi162Scf00034g02114	Pax_COMT20_c0	369	1	41.68	6.04	0

Species	Subfamily	Original Gene ID	Putative Name	Protein length	Intron numbers	Mw (kDa)	pI	Chromosomal location
		Peaxi162Scf00078g00838	Pax_COMT21_c0	351	3	39.29	5.94	0
		Peaxi162Scf00791g00421	Pax_COMT22_c0	350	1	39.37	5.71	0
		Peaxi162Scf00229g00076	Pax_COMT23_c0	368	2	40.16	5.25	0
		Peaxi162Scf00010g00005	Pax_COMT24_c0	350	2	39.70	5.85	0
		Peaxi162Scf01122g00019	Pax_COMT25_c0	340	1	37.79	5.58	0
		Peaxi162Scf00833g00056	Pax_COMT26_c0	263	0	28.66	5.50	0
		Peaxi162Scf65040g00001	-	184	-	-	-	0
		Peaxi162Scf00017g00118	Pax_COMT27_c0	291	2	32.55	6.26	0
		Peaxi162Scf00004g00465	Pax_COMT28_c0	561	1	63.58	6.99	0
		Peaxi162Scf59115g00001	-	100	-	-	-	0
		Peaxi162Scf00791g00430	-	107	-	-	-	0
		Peaxi162Scf00791g00417	-	111	-	-	-	0
		Peaxi162Scf61343g00001	-	100	-	-	-	0
		Peaxi162Scf00010g00028	Pax_COMT29_c0	220	2	24.87	6.29	0
		Peaxi162Scf00571g00523	Pax_COMT30_c0	479	11	54.50	5.72	0
		Peaxi162Scf00241g00812	Pax_COMT31_c0	365	7	40.79	6.62	0
		Peaxi162Scf00683g00459	Pax_COMT32_c0	471	11	53.51	6.41	0
		Peaxi162Scf55153g00001	-	40	-	-	-	0
		Peaxi162Scf00078g00911	Pax_COMT33_c0	208	2	23.42	4.98	0
Solanum lycopersicum	CCoAOMT	Solyc10g050160	Sly_CCoAOMT10_c10	245	4	27.83	5.14	10
		Solyc02g093250	Sly_CCoAOMT3_c2	242	4	27.25	5.17	2
		Solyc02g093270	Sly_CCoAOMT4_c2	304	4	34.31	5.55	2
		Solyc02g093230	Sly_CCoAOMT2_c2	242	4	27.33	5.17	2
		Solyc09g082660	Sly_CCoAOMT9_c9	235	5	26.36	5.52	9
		Solyc03g032220	Sly_CCoAOMT5_c3	234	4	26.33	5.08	3
		Solyc04g063210	Sly_CCoAOMT6_c4	282	3	31.79	5.41	4
		Solyc08g006830	Sly_CCoAOMT8_c6	288	9	32.28	8.51	6
		Solyc05g026330	Sly_CCoAOMT7_c5	187	2	20.95	5.39	5
		Solyc05g041620	Sly_CCoAOMT8_c5	233	2	26.39	5.41	5
		Solyc05g041950	-	164	-	-	-	5

Species	Subfamily	Original Gene ID	Putative Name	Protein length	Intron numbers	Mw (kDa)	pI	Chromosomal location
		Solyc05g041640	-	178	-	-	-	5
		Solyc05g041650	-	136	-	-	-	5
		Solyc05g041610	-	116	-	-	-	5
		Solyc05g026350	-	158	-	-	-	5
		Solyc05g041670	-	88	-	-	-	5
		Solyc05g041690	-	156	-	-	-	5
		Solyc02g093240	-	86	-	-	-	2
		Solyc05g041320	-	89	-	-	-	5
		Solyc05g026040	-	100	-	-	-	5
		Solyc05g041270	-	70	-	-	-	5
		Solyc05g025950	-	51	-	-	-	5
		Solyc05g041300	-	129	-	-	-	5
		Solyc05g161510	-	104	-	-	-	5
		Solyc05g041660	-	58	-	-	-	5
		Solyc05g026000	-	56	-	-	-	5
		Solyc05g025990	-	50	-	-	-	5
		Solyc05g041260	-	44	-	-	-	5
		Solyc01g095920	Sly_CCoAOMT1_c1	227	0	24.84	7.66	1
	COMT	Solyc03g080180	Sly_COMT6_c3	357	3	38.95	5.63	3
		Solyc10g005060	Sly_COMT15_c10	364	3	40.34	5.47	10
		Solyc03g097700	Sly_COMT7_c3	445	1	49.99	6.73	3
		Solyc02g077530	Sly_COMT5_c2	360	1	40.71	5.56	2
		Solyc10g008120	Sly_COMT17_c10	362	1	40.29	5.61	10
		Solyc06g007960	Sly_COMT9_c6	361	3	40.72	5.69	6
		Solyc02g077520	Sly_COMT4_c2	357	1	40.20	5.46	2
		Solyc12g041960	Sly_COMT23_c12	362	1	40.84	6.27	12
		Solyc01g111900	Sly_COMT2_c1	357	2	38.71	5.92	1
		Solyc06g064500	Sly_COMT10_c6	356	1	39.49	5.20	6
		Solyc10g079540	Sly_COMT18_c10	355	1	40.30	4.73	10
		Solyc01g068550	Sly_COMT1_c1	352	1	39.06	5.13	1

Species	Subfamily	Original Gene ID	Putative Name	Protein length	Intron numbers	Mw (kDa)	pI	Chromosomal location
		Solyc06g064510	Sly_COMT11_c6	355	1	39.46	5.07	6
		Solyc06g083450	-	193	-	-	-	6
		Solyc12g009110	Sly_COMT20_c12	369	2	40.57	5.44	12
		Solyc10g085830	Sly_COMT19_c10	361	3	40.20	5.58	10
		Solyc06g060200	-	125	-	-	-	6
		Solyc06g060215	-	130	-	-	-	6
		Solyc12g041940	Sly_COMT22_c12	249	0	27.98	5.69	12
		Solyc12g041950	-	103	-	-	-	12
		Solyc09g056230	-	138	-	-	-	9
		Solyc10g047520	-	101	-	-	-	10
		Solyc12g160770	-	102	-	-	-	12
		Solyc00g005285	-	71	-	-	-	0
		Solyc12g040790	Sly_COMT21_c12	500	11	56.81	5.70	12
		Solyc03g118750	Sly_COMT8_c3	491	11	55.95	6.12	3
		Solyc10g161210	-	107	-	-	-	10
		Solyc02g077510	Sly_COMT3_c2	216	2	24.09	5.24	2
		Solyc10g008020	Sly_COMT16_c10	381	7	42.60	6.42	10
		Solyc09g065730	Sly_COMT14_c9	339	2	37.99	6.40	9
		Solyc06g068950	Sly_COMT12_c6	362	11	41.83	5.44	6
		Solyc08g062770	Sly_COMT13_c8	554	10	62.30	5.37	8
