# Supplementary material for: Evolution and diversification of the O-methyltransferase (OMT) gene family in Solanaceae
Source: Genet Mol Biol. 2023 Nov 10;46(3 Suppl 1):e20230121. doi: 10.1590/1678-4685-GMB-2023-0121 (PMC10637433; doi:10.1590/1678-4685-GMB-2023-0121)
Supplement: Table S4 - [file 1415-4757-GMB-46-3-s1-e20230121-s4.pdf]

Genetics and Molecular Biology
https://doi.org/10.1590/1678-4685-GMB-2023-0121

Supplementary Material to "Evolution and diversification of the O-methyltransferase (OMT) gene family in Solanaceae"

Table S4 - Putative substrate, biological and molecular processes associated with the phylogenetic groups of the OMT family of Solanaceae species. GO terms and catalytic activity in bold indicate processes present in the outgroup.

Subfamily	Group	Biological Function	Molecular Function	Catalytic activity
CCoAOMT	I	-	-	-
	II	circadian rhythm [GO:0007623] coumarin biosynthetic process [GO:0009805] green leaf volatile biosynthetic process [GO:0010597] **lignin biosynthetic process [GO:0009809]** **methylation [GO:0032259]** phenylpropanoid metabolic process [GO:0009698]	caffeoyl CoA:S-adenosyl-L-methionine O-methyltransferase activity [GO:0080076] **caffeoyl-CoA O-methyltransferase activity [GO:0042409]** **metal ion binding [GO:0046872]** **S-adenosylmethionine-dependent methyltransferase activity [GO:0008757]**	**(E)-caffeoyl-CoA + S-adenosyl-L-methionine = (E)-feruloyl-CoA + H(+) + S-adenosyl-L-homocysteine**
	III	circadian rhythm [GO:0007623] green leaf volatile biosynthetic process [GO:0010597] **lignin biosynthetic process [GO:0009809]** **methylation [GO:0032259]** phenylpropanoid metabolic process [GO:0009698]	caffeoyl CoA:S-adenosyl-L-methionine O-methyltransferase activity [GO:0080076] **caffeoyl-CoA O-methyltransferase activity [GO:0042409]** **metal ion binding [GO:0046872]** **S-adenosylmethionine-dependent methyltransferase activity [GO:0008757]**	**(E)-caffeoyl-CoA + S-adenosyl-L-methionine = (E)-feruloyl-CoA + H(+) + S-adenosyl-L-homocysteine**
	IV	**lignin biosynthetic process [GO:0009809]** **methylation [GO:0032259]**	**caffeoyl-CoA O-methyltransferase activity [GO:0042409]** **metal ion binding [GO:0046872]** **S-adenosylmethionine-dependent methyltransferase activity [GO:0008757]**	**(E)-caffeoyl-CoA + S-adenosyl-L-methionine = (E)-feruloyl-CoA + H(+) + S-adenosyl-L-homocysteine**

Subfamily	Group	Biological Function	Molecular Function	Catalytic activity
	V	**lignin biosynthetic process [GO:0009809]**<br>**methylation [GO:0032259]**	**caffeoyl-CoA O-methyltransferase activity [GO:0042409]**<br>**metal ion binding [GO:0046872]**<br>**S-adenosylmethionine-dependent methyltransferase activity [GO:0008757]**	**(E)-caffeoyl-CoA + S-adenosyl-L-methionine = (E)-feruloyl-CoA + H(+) + S-adenosyl-L-homocysteine**
	VI	anthocyanin-containing compound biosynthetic process [GO:0009718]<br>cyanidin 3-O-glucoside biosynthetic process [GO:0033485]<br>delphinidin 3-O-glucoside biosynthetic process [GO:0033486]<br>**lignin biosynthetic process [GO:0009809]**<br>**methylation [GO:0032259]**<br>pigmentation [GO:0043473]<br>seed development [GO:0048316]<br>spermidine hydroxycinnamate conjugate biosynthetic process [GO:0080088]	caffeoyl CoA:S-adenosyl-L-methionine O-methyltransferase activity [GO:0080076]<br>caffeoyl-CoA O-methyltransferase activity [GO:0042409]<br>laricitrin 5'-O-methyltransferase activity [GO:0070448]<br>metal ion binding [GO:0046872]<br>myricetin 3'-O-methyltransferase activity [GO:0033799]<br>O-methyltransferase activity [GO:0008171]<br>S-adenosylmethionine-dependent methyltransferase activity [GO:0008757]<br>tricaffeoyl spermidine:S-adenosyl-L-methionine O-methyltransferase activity [GO:0080078]<br>trihydroxyferuloyl spermidine O-methyltransferase activity [GO:0080012]<br>trihydroxyferuloyl spermidine:S-adenosyl-L-methionine O-methyltransferase activity [GO:0080077]	S-adenosyl-L-methionine + (E)-caffeoyl-CoA = (E)-feruloyl-CoA + H(+) + S-adenosyl-L-homocysteine<br>S-adenosyl-L-methionine + a 3'-hydroxyflavonoid = S-adenosyl-L-homocysteine + a 3'-methoxyflavonoid
	VII	-	-	
COMT	I	**alkaloid metabolic process [GO:0009820]**<br>aromatic compound biosynthetic process [GO:0019438]<br>isoflavonoid biosynthetic process [GO:0009717]<br>isoflavonoid phytoalexin biosynthetic process [GO:0009701]<br>methylation [GO:0032259]	2,7,4'-trihydroxyisoflavanone-4'-O-methyltransferase activity [GO:0102670]<br>6a-hydroxymaackiain-3-O-methyltransferase activity [GO:0102671]<br>O-methyltransferase activity [GO:0008171]<br>S-adenosylmethionine-dependent methyltransferase activity [GO:0008757]<br>flavanone 4'-O-methyltransferase activity [GO:0102767]<br>isoflavone 4'-O-methyltransferase activity	(+)-6a-hydroxymaackiain + S-adenosyl-L-methionine = (+)-pisatin + H(+) + S-adenosyl-L-homocysteine<br>(-)-pluviatolide + S-adenosyl-L-methionine = (-)-bursehernin + H(+) + S-adenosyl-L-homocysteine<br>a 4'-hydroxyflavanone + S-adenosyl-L-methionine = a 4'-methoxyflavanone + H(+) + S-adenosyl-L-homocysteine<br>a 7-hydroxyisoflavone + S-adenosyl-L-methionine = a 7-methoxyisoflavone + H(+) + S-adenosyl-L-homocysteine

Subfamily	Group	Biological Function	Molecular Function	Catalytic activity
		phenylpropanoid biosynthetic process [GO:0009699]<br>**response to cold [GO:0009409]**<br>**response to high light intensity [GO:0009644]**<br>response to wounding [GO:0009611]<br>**secondary metabolite biosynthetic process [GO:0044550]**	[GO:0030746]<br>isoflavone 7-O-methyltransferase activity [GO:0033800]<br>protein dimerization activity [GO:0046983]	
	II	alkaloid biosynthetic process [GO:0009821]<br>**aromatic compound biosynthetic process [GO:0019438]**<br>methylation [GO:0032259]<br>**response to cold [GO:0009409]**<br>**response to high light intensity [GO:0009644]**<br>**secondary metabolite biosynthetic process [GO:0044550]**	11-O-demethyl-17-O-deacetylvindoline O-methyltransferase activity [GO:0030766]<br>O-methyltransferase activity [GO:0008171]<br>laricitrin 5'-O-methyltransferase activity [GO:0070448]<br>myricetin 3'-O-methyltransferase activity [GO:0033799]<br>protein dimerization activity [GO:0046983]<br>protein homodimerization activity [GO:0042803]	16-hydroxytabersonine + S-adenosyl-L-methionine = 16-methoxytabersonine + H(+) + S-adenosyl-L-homocysteine<br>S-adenosyl-L-methionine + a 3'-hydroxyflavonoid = S-adenosyl-L-homocysteine + a 3'-methoxyflavonoid<br>**(8Z,11Z)-5-(pentadeca-8,11,14-trien-1-yl)resorcinol + S-adenosyl-L-methionine = (8Z,11Z)-5-(pentadeca- 8,11,14-trien-1-yl)resorcinol-3-methyl ether + H(+) + S-adenosyl-L-homocysteine**<br>**(S)-norcoclaurine + S-adenosyl-L-methionine = (S)-coclaurine + H(+) + S-adenosyl-L-homocysteine**<br>**7,8-dihydroxycoumarin + S-adenosyl-L-methionine = 7-hydroxy-8-methoxycoumarin + H(+) + S-adenosyl-L-homocysteine**<br>**S-adenosyl-L-methionine + TRIBOA beta-D-glucoside = DIMBOA beta-D-glucoside + H(+) + S-adenosyl-L-homocysteine**
	III	**alkaloid metabolic process [GO:0009820]**<br>aromatic compound biosynthetic process [GO:0019438]<br>flavonoid metabolic process [GO:0009812]<br>methylation [GO:0032259]<br>phenylpropanoid biosynthetic process [GO:0009699]<br>**response to cold [GO:0009409]**<br>**response to high light**	(iso)eugenol O-methyltransferase activity [GO:0050630]<br>8-hydroxyquercitin 8-O-methyltransferase activity [GO:0030761]<br>O-methyltransferase activity [GO:0008171]<br>S-adenosyl-L-methionine:eugenol-O-methyltransferase activity [GO:0102719]<br>S-adenosylmethionine-dependent methyltransferase activity [GO:0008757]<br>protein dimerization activity [GO:0046983]	2 S-adenosyl-L-methionine + trans-resveratrol = 2 H(+) + pterostilbene + 2 S-adenosyl-L-homocysteine<br>3,3',4',5,7,8-hexahydroxyflavone + S-adenosyl-L-methionine = 3,3',4',5,7-pentahydroxy-8-methoxyflavone + H(+) + S-adenosyl-L-homocysteine<br>S-adenosyl-L-methionine + trans-isoeugenol = H(+) + S-adenosyl-L-homocysteine + trans-isomethyleugenol<br>**(8Z,11Z)-5-(pentadeca-8,11,14-trien-1-yl)resorcinol + S-adenosyl-L-methionine = (8Z,11Z)-5-(pentadeca- 8,11,14-trien-1-yl)resorcinol-3-methyl ether + H(+) + S-adenosyl-L-homocysteine**<br>**(S)-norcoclaurine + S-adenosyl-L-methionine = (S)-coclaurine + H(+) + S-adenosyl-L-homocysteine**

Subfamily	Group	Biological Function	Molecular Function	Catalytic activity
		**intensity [GO:0009644]**<br>**secondary metabolite biosynthetic process [GO:0044550]**	resveratrol 3,5-O-dimethyltransferase activity [GO:0102303]	**7,8-dihydroxycoumarin + S-adenosyl-L-methionine = 7-hydroxy-8-methoxycoumarin + H(+) + S-adenosyl-L-homocysteine**<br>**S-adenosyl-L-methionine + TRIBOA beta-D-glucoside = DIMBOA beta-D-glucoside + H(+) + S-adenosyl-L-homocysteine**
	IV	**alkaloid metabolic process [GO:0009820]**<br>**lignin biosynthetic process [GO:0009809]**<br>**methylation [GO:0032259]**	**(RS)-norcoclaurine 6-O-methyltransferase activity [GO:0030786]**<br>**(S)-scoulerine 9-O-methyltransferase activity [GO:0030777]**<br>**3'-hydroxy-N-methyl-(S)-coclaurine 4'-O-methyltransferase activity [GO:0030784]**<br>**O-methyltransferase activity [GO:0008171]**<br>**caffeate O-methyltransferase activity [GO:0047763]**<br>**columbamine O-methyltransferase activity [GO:0030778]**<br>**protein dimerization activity [GO:0046983]**<br>**tetrahydrocolumbamine 2-O-methyltransferase activity [GO:0030762]**	(E)-caffeate + S-adenosyl-L-methionine = (E)-ferulate + H(+) + S-adenosyl-L-homocysteine<br>(S)-3'-hydroxy-N-methylcoclaurine + S-adenosyl-L-methionine = (S)-reticuline + H(+) + S-adenosyl-L-homocysteine<br>(S)-scoulerine + S-adenosyl-L-methionine = (S)-tetrahydrocolumbamine + H(+) + S-adenosyl-L-homocysteine<br>columbamine + S-adenosyl-L-methionine = H(+) + palmatine + S-adenosyl-L-homocysteine<br>norcoclaurine + S-adenosyl-L-methionine = coclaurine + H(+) + S-adenosyl-L-homocysteine
	V	**alkaloid metabolic process [GO:0009820]**<br>aromatic compound biosynthetic process [GO:0019438]<br>benzyl isoquinoline alkaloid biosynthetic process [GO:0009708]<br>coumarin biosynthetic process [GO:0009805]<br>flavonoid biosynthetic process [GO:0009813]<br>flavonoid metabolic process [GO:0009812]<br>flavonol biosynthetic process	**(RS)-norcoclaurine 6-O-methyltransferase activity [GO:0030786]**<br>(S)-scoulerine 9-O-methyltransferase activity [GO:0030777]<br>(iso)eugenol O-methyltransferase activity [GO:0050630]<br>**3'-hydroxy-N-methyl-(S)-coclaurine 4'-O-methyltransferase activity [GO:0030784]**<br>5-hydroxyfuranocoumarin 5-O-methyltransferase activity [GO:0030752]<br>L-dopa O-methyltransferase activity [GO:0102084]<br>O-methyltransferase activity	(-)-5'-demethylyatein + S-adenosyl-L-methionine = (-)-yatein + H(+) + S-adenosyl-L-homocysteine<br>(2S)-naringenin + S-adenosyl-L-methionine = (2S)-sakuranetin + H(+) + S-adenosyl-L-homocysteine<br>(E)-5-hydroxyferulate + S-adenosyl-L-methionine = (E)-sinapate + H(+) + S-adenosyl-L-homocysteine<br>(E)-caffeate + S-adenosyl-L-methionine = (E)-ferulate + H(+) + S-adenosyl-L-homocysteine<br>**(S)-3'-hydroxy-N-methylcoclaurine + S-adenosyl-L-methionine = (S)-reticuline + H(+) + S-adenosyl-L-homocysteine**<br>(S)-scoulerine + S-adenosyl-L-methionine = (S)-tetrahydrocolumbamine + H(+) + S-adenosyl-L-homocysteine<br>3 S-adenosyl-L-methionine + tricetin = 3',4',5'-O-trimethyltricetin + 3 H(+) + 3 S-adenosyl-L-homocysteine

Subfamily	Group	Biological Function	Molecular Function	Catalytic activity
		[GO:0051555]<br>lignin biosynthetic process [GO:0009809]<br>melatonin biosynthetic process [GO:0030187]<br>methylation [GO:0032259]<br>phenylpropanoid biosynthetic process [GO:0009699]<br>regulation of lignin biosynthetic process [GO:1901141]<br>response to UV [GO:0009411]<br>response to ethylene [GO:0009723]<br>response to hydrogen peroxide [GO:0042542]<br>response to jasmonic acid [GO:0009753]<br>response to salicylic acid [GO:0009751]<br>response to wounding [GO:0009611]	[GO:0008171]<br>S-adenosyl-L-methionine:eugenol-O-methyltransferase activity [GO:0102719]<br>S-adenosylmethionine-dependent methyltransferase activity [GO:0008757]<br>acetylserotonin O-methyltransferase activity [GO:0017096]<br>anthranilate N-methyltransferase activity [GO:0030774]<br>caffeate O-methyltransferase activity [GO:0047763]<br>catechol O-methyltransferase activity [GO:0016206]<br>**columbamine O-methyltransferase activity [GO:0030778]**<br>isoliquiritigenin 2'-O-methyltransferase activity [GO:0033802]<br>licodione 2'-O-methyltransferase activity [GO:0030751]<br>luteolin O-methyltransferase activity [GO:0030744]<br>methyltransferase activity [GO:0008168]<br>myricetin 3'-O-methyltransferase activity [GO:0033799]<br>naringenin 7-O-methyltransferase activity [GO:0102766]<br>orcinol O-methyltransferase activity [GO:0102938]<br>protein dimerization activity [GO:0046983]<br>quercetin 3'-O-methyltransferase activity [GO:0102822]<br>quercetin 3-O-methyltransferase activity [GO:0030755]<br>tetrahydrocolumbamine 2-O-methyltransferase activity [GO:0030762]<br>tricetin O-methytransferase activity [GO:0102146]	S-adenosyl-L-methionine + trans-anol = H(+) + S-adenosyl-L-homocysteine + trans-anethole<br>S-adenosyl-L-methionine + trans-isoeugenol = H(+) + S-adenosyl-L-homocysteine + trans-isomethyleugenol<br>a 3'-hydroxyflavone + S-adenosyl-L-methionine = a 3'-methoxyflavone + H(+) + S-adenosyl-L-homocysteine<br>a 5-hydroxyfurocoumarin + S-adenosyl-L-methionine = a 5-methoxyfurocoumarin + H(+) + S-adenosyl-L-homocysteine<br>anthranilate + S-adenosyl-L-methionine = H(+) + N-methylanthranilate + S-adenosyl-L-homocysteine<br>bergaptol + S-adenosyl-L-methionine = bergapten + S-adenosyl-L-homocysteine<br>catechol + S-adenosyl-L-methionine = guaiacol + H(+) + S-adenosyl-L-homocysteine<br>**columbamine + S-adenosyl-L-methionine = H(+) + palmatine + S-adenosyl-L-homocysteine**<br>isoliquiritigenin + S-adenosyl-L-methionine = 2'-O-methylisoliquiritigenin + S-adenosyl-L-homocysteine<br>**norcoclaurine + S-adenosyl-L-methionine = coclaurine + H(+) + S-adenosyl-L-homocysteine**
