# Supplementary material for: Evolution and diversification of the O-methyltransferase (OMT) gene family in Solanaceae
Source: Genet Mol Biol. 2023 Nov 10;46(3 Suppl 1):e20230121. doi: 10.1590/1678-4685-GMB-2023-0121 (PMC10637433; doi:10.1590/1678-4685-GMB-2023-0121)
Supplement: Figure S1 - [file 1415-4757-GMB-46-3-s1-e20230121-s5.zip › gmb-2023-0121_suppl5_FigS1.pdf]

Genetics and Molecular Biology
https://doi.org/10.1590/1678-4685-GMB-2023-0121

Supplementary Material to "Evolution and diversification of the O-methyltransferase (OMT) gene family in Solanaceae"
