# Supplementary figures and images for: Evolution and diversification of the O-methyltransferase (OMT) gene family in Solanaceae
Source: Genet Mol Biol. 2023 Nov 10;46(3 Suppl 1):e20230121. doi: 10.1590/1678-4685-GMB-2023-0121 (PMC10637433; doi:10.1590/1678-4685-GMB-2023-0121)

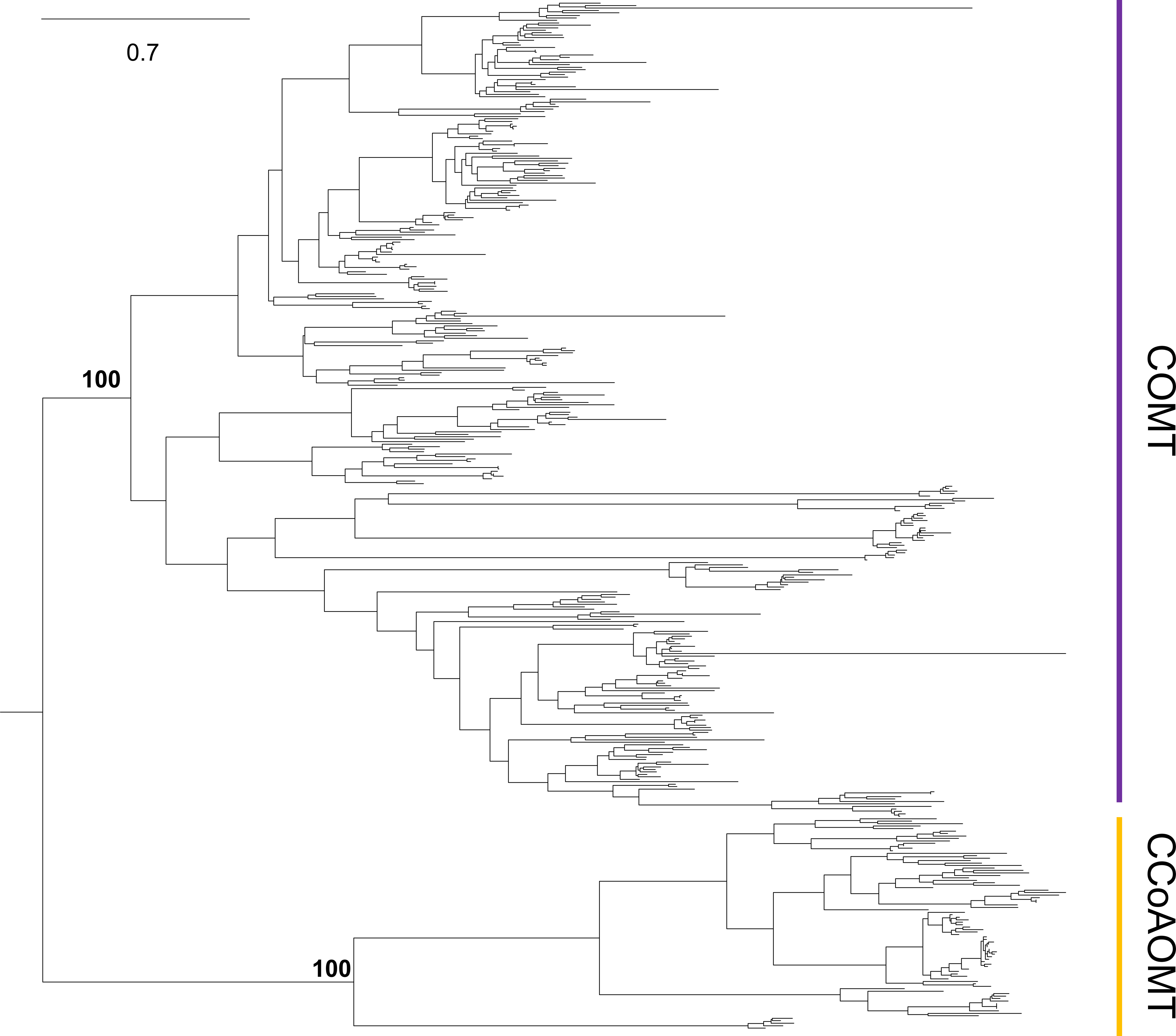

Supplement: Figure S1 - [file 1415-4757-GMB-46-3-s1-e20230121-s5.zip › FigureS1_R1.tiff]

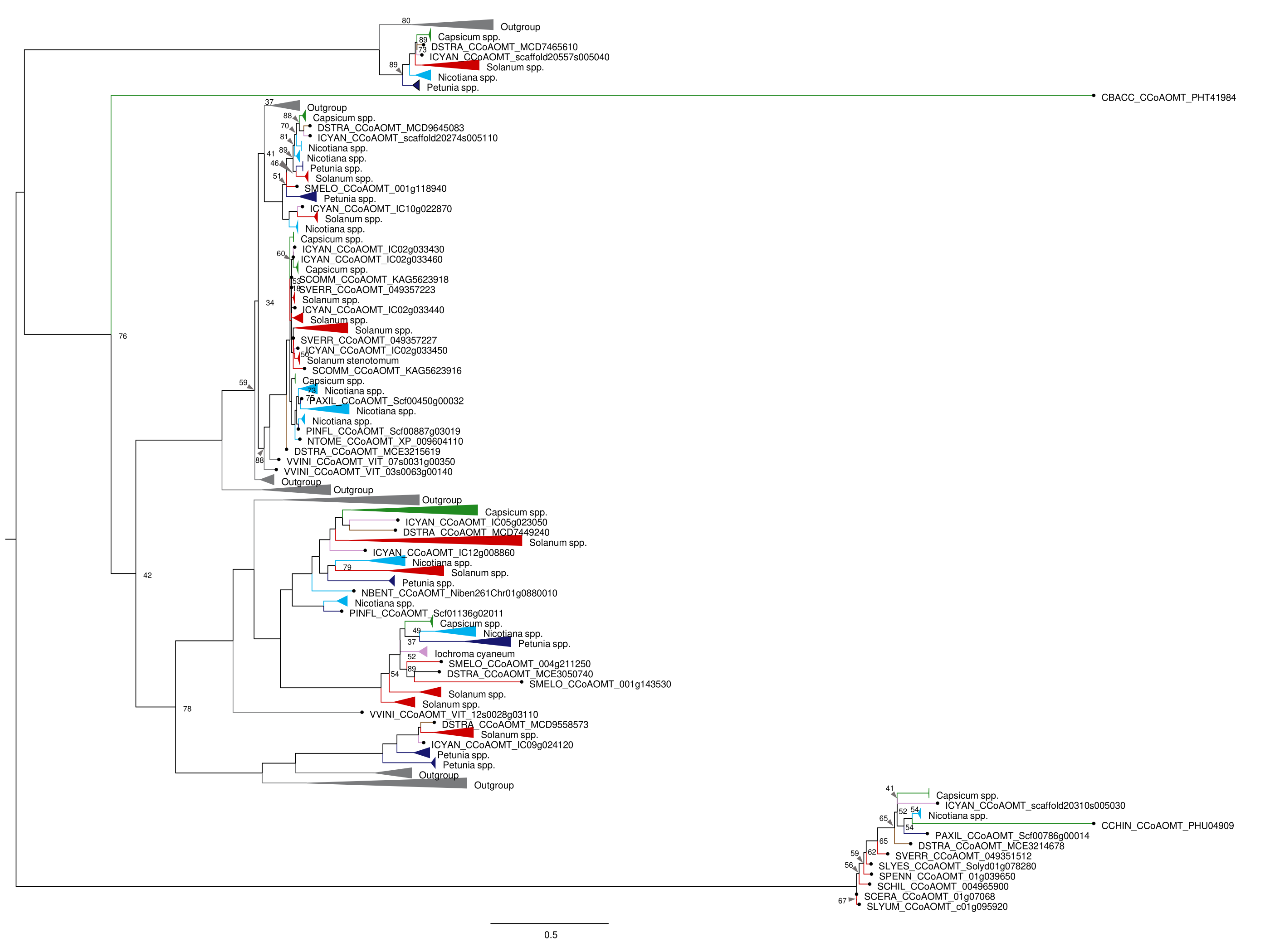

Supplement: Figure S2 - [file 1415-4757-GMB-46-3-s1-e20230121-s6.zip › FigureS2_R1.tiff]

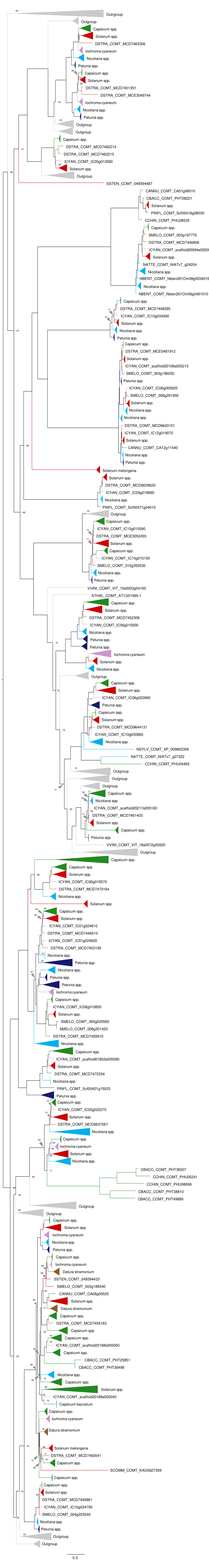

Supplement: Figure S3 - [file 1415-4757-GMB-46-3-s1-e20230121-s7.zip › FigureS3_R1.tiff]

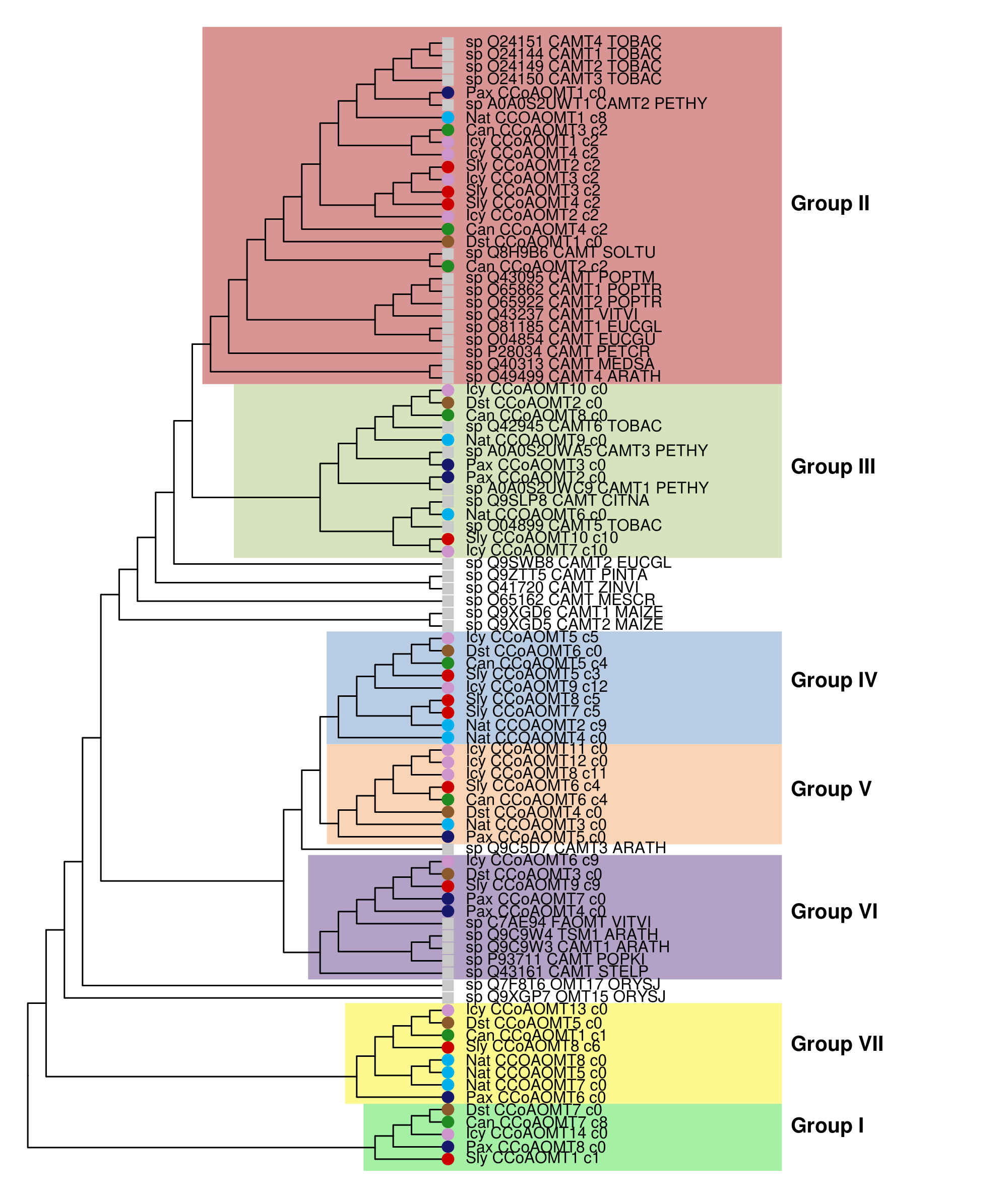

Supplement: Figure S4 - [file 1415-4757-GMB-46-3-s1-e20230121-s8.zip › FigureS4_R1.tiff]

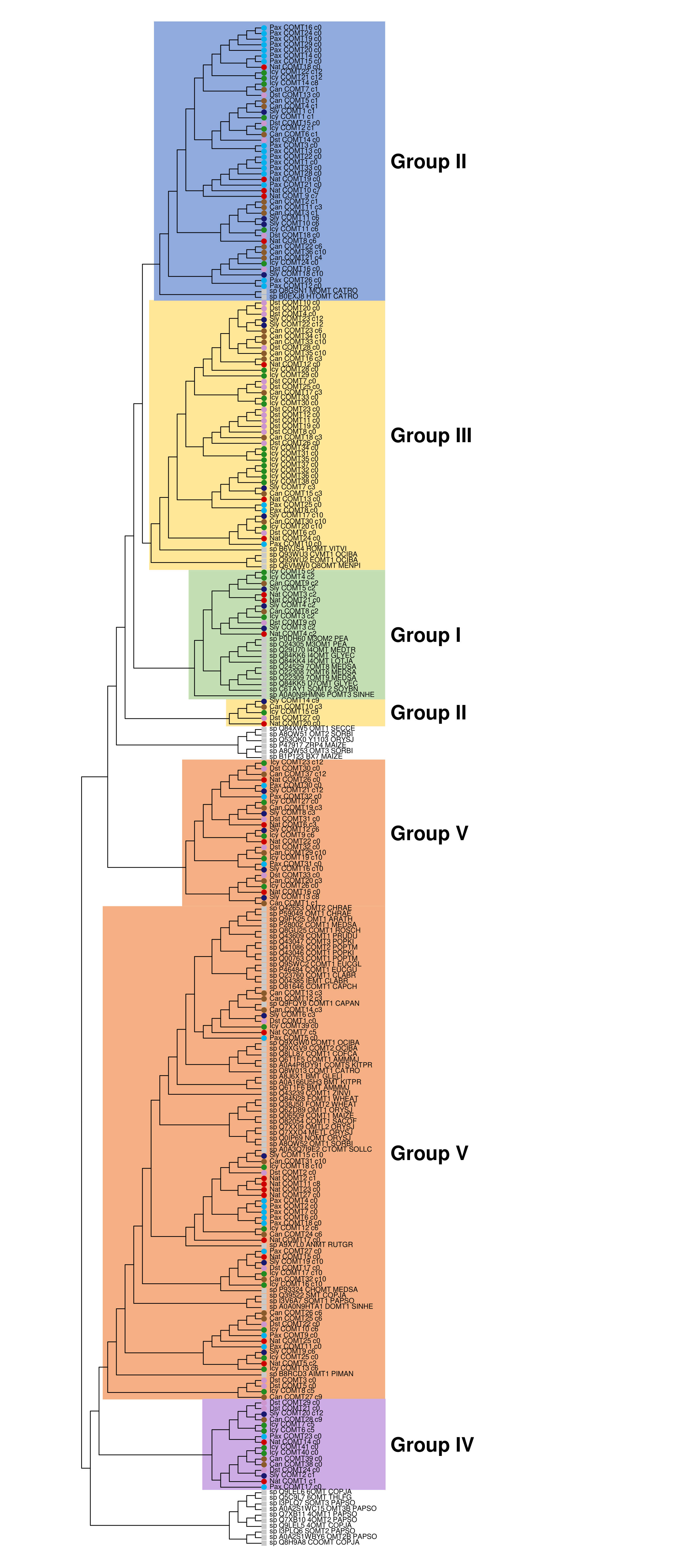

Supplement: Figure S5 - [file 1415-4757-GMB-46-3-s1-e20230121-s9.zip › FigureS5_R1.tiff]

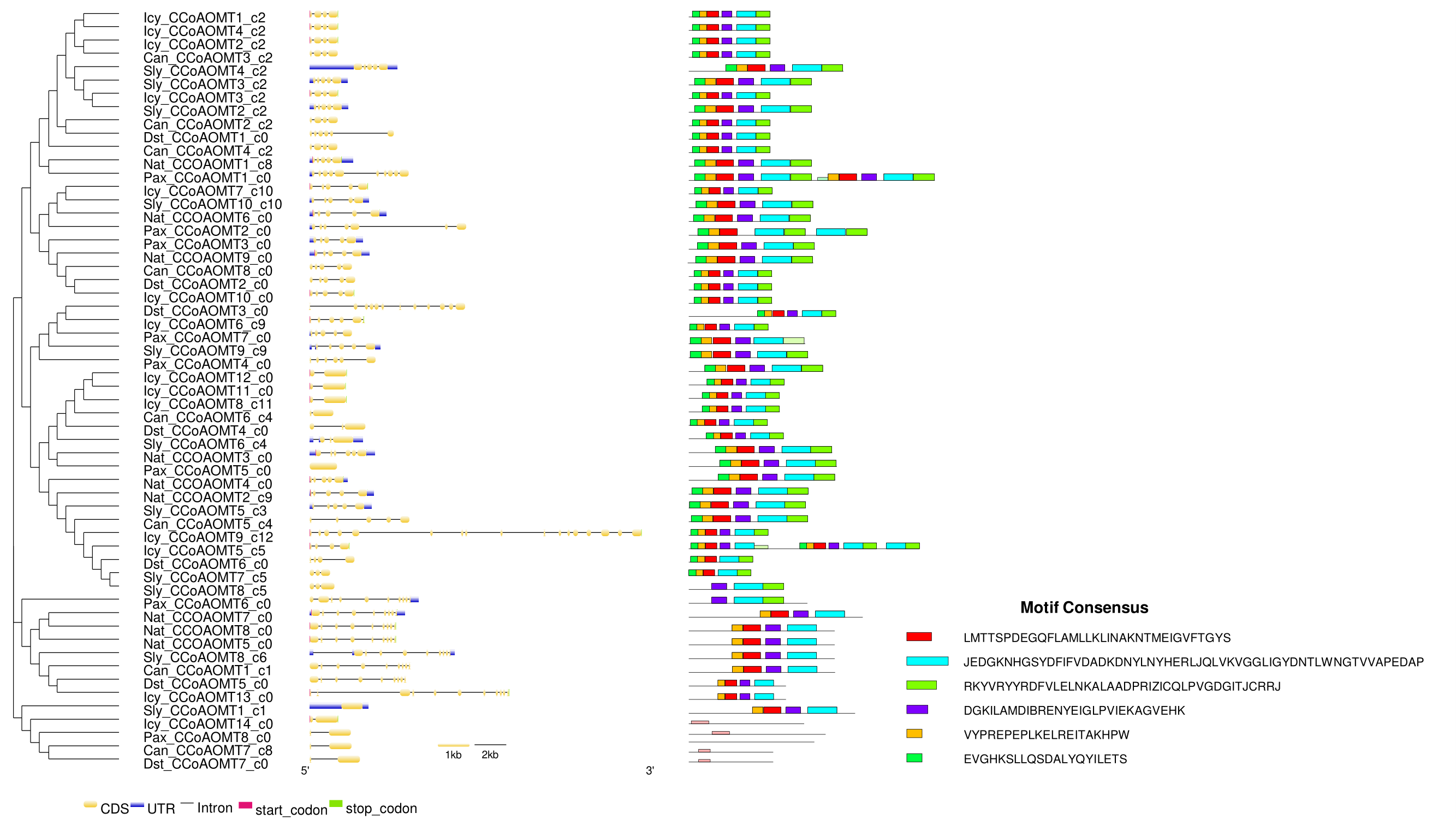

Supplement: Figure S6 - [file 1415-4757-GMB-46-3-s1-e20230121-s10.zip › FigureS6_R1.tiff]

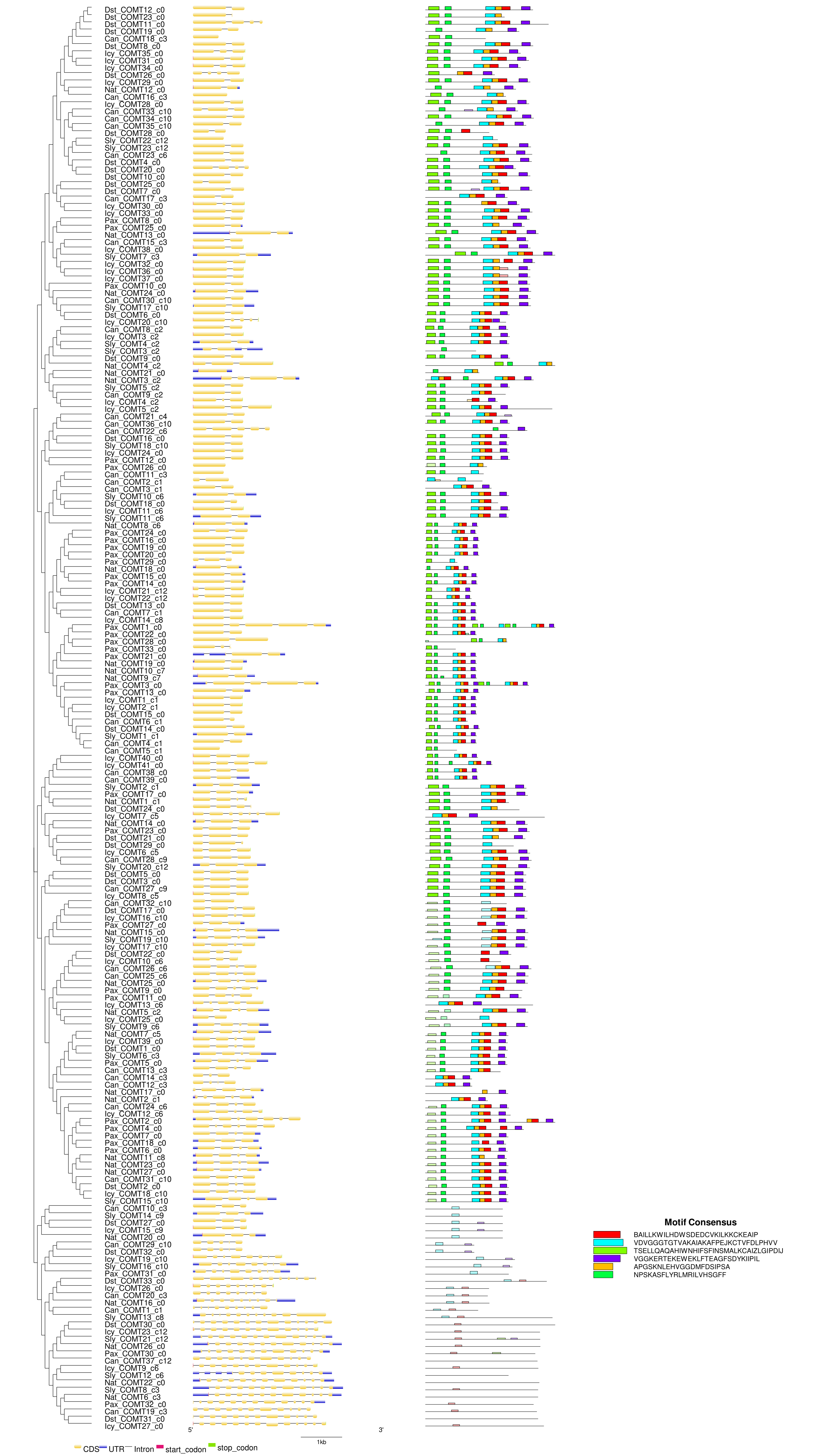

Supplement: Figure S7 - [file 1415-4757-GMB-46-3-s1-e20230121-s11.zip › FigureS7_R1.tiff]
